# Pulsed Dynamic Water Electrolysis: Mass Transfer Enhancement, Microenvironment Regulation, and Hydrogen Production Optimization

**DOI:** 10.1007/s40820-025-01952-5

**Published:** 2026-01-07

**Authors:** Xuewei Zhang, Wei Zhou, Xiaoxiao Meng, Yuming Huang, Yang Yu, Haiqian Zhao, Lijie Wang, Fei Sun, Jihui Gao, Guangbo Zhao

**Affiliations:** 1 School of Energy Science and Engineering, Harbin Institute of Technology, Harbin, Heilongjiang, 150001 People’s Republic of China; 2 School of Environmental Science and Engineering, Suzhou University of Science and Technology, Suzhou, 215009 People’s Republic of China; 3 China Datang Technology Innovation Co., Ltd., Xiong’an, 071799 People’s Republic of China

**Keywords:** Pulsed dynamic electrolysis, Water electrolysis, Energy and mass transfer, Microenvironment, System stability

## Abstract

Pulsed dynamic electrolysis (PDE), driven by renewable energy, has emerged as an innovative electrocatalytic conversion method, demonstrating significant potential in addressing global energy challenges and promoting sustainable development. Despite significant progress in various electrochemical systems, the regulatory mechanisms of PDE in energy and mass transfer and the lifespan extension of electrolysis systems, particularly in water electrolysis (WE) for hydrogen production, remain insufficiently explored. Therefore, there is an urgent need for a deeper understanding of the unique contributions of PDE in mass transfer enhancement, microenvironment regulation, and hydrogen production optimization, aiming to achieve low-energy consumption, high catalytic activity, and long-term stability in the generation of target products. Here, this review critically examines the microenvironmental effects of PDE on energy and mass transfer, the electrode degradation mechanisms in the lifespan extension of electrolysis systems, and the key factors in enhancing WE for hydrogen production, providing a comprehensive summary of current research progress. The review focuses on the complex regulatory mechanisms of frequency, duty cycle, amplitude, and other factors in hydrogen evolution reaction (HER) performance within PDE strategies, revealing the interrelationships among them. Finally, the potential future directions and challenges for transitioning from laboratory studies to industrial applications are proposed.

## Highlights

- The mechanisms, key factors, and merits of pulsed dynamic electrolysis (PDE) in energy and mass transfer, extending system lifespan, and enhancing water electrolysis are covered.
- Synergies and parameter-performance relationships between PDE and hydrogen evolution reaction are emphasized.
- Future prospects and challenges for the development of PDE technology are outlined.

## Graphical abstract

**Figure:**
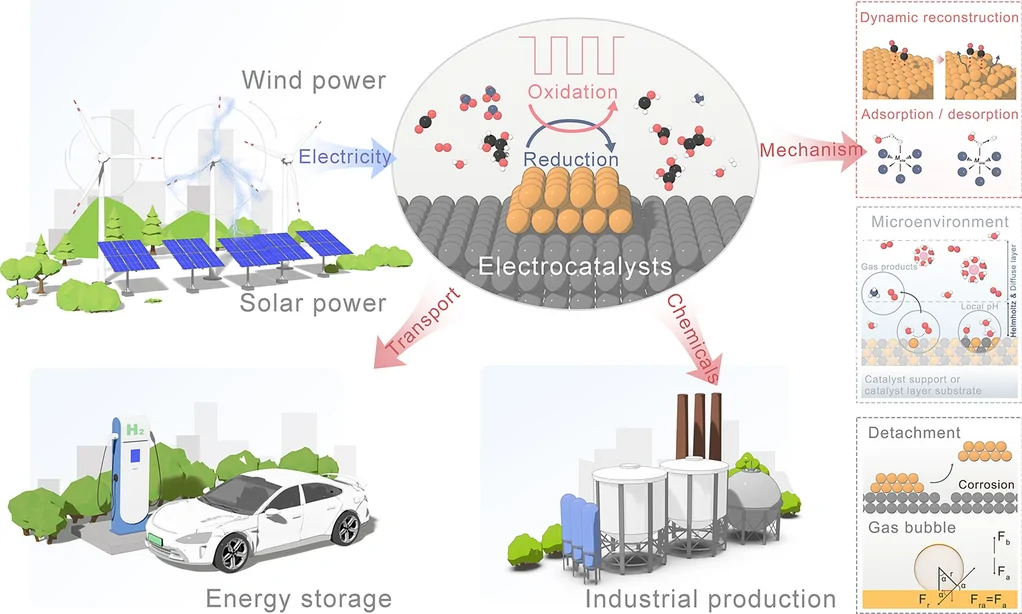

## Introduction

With the sharp rise in global energy demand and the increasingly severe climate change, the development of renewable energy conversion technologies aligned with the “dual carbon” goals has become particularly urgent [1, 2]. Electrocatalysis, a technology that promotes chemical reactions through electrochemical processes, has demonstrated immense potential in the utilization and storage of renewable energy, especially in fields such as hydrogen production [3], battery storage [4], and carbon dioxide reduction [5, 6]. As a result, renewable energy-driven electrocatalysis is regarded as a green, low-carbon solution and is widely considered an effective way to address the environmental pollution and energy shortages caused by excessive reliance on fossil fuels. Compared to traditional energy conversion technologies, renewable energy-driven electrocatalysis offers advantages such as low-energy consumption [7], low-carbon emissions [8], and high efficiency [9]. However, to realize the commercialization and industrialization of this technology, many challenges remain. To meet practical production demands, it is necessary to reduce overpotentials, increase current densities during electrolysis, improve product selectivity and Faradaic efficiency (FE), and enhance catalytic stability.

To enhance the performance of electrocatalysis, researchers have made significant efforts in catalyst design [10], microenvironmental regulation at the interface [11], and reactor design [12]. Meanwhile, in recent years, an increasing number of researchers have begun to focus on the effect of the power supply side on electrocatalytic performance, particularly the nature of the electrolysis process [13, 14]. Conventional electrochemical processes are usually conducted under steady-state conditions [15], with common operating modes including constant current and constant potential electrolysis (CE). These methods have been widely used and can provide stable operation in many cases. However, the dynamic characteristics of renewable energy, such as fluctuation and instability, present new challenges for electrochemical processes. Unlike conventional CE operation modes, dynamic operation can better cope with the fluctuation of renewable energy, making it especially important for energy transition [16, 17]. As a result, dynamic operation modes have gradually become a highly focused direction to address the challenges of energy transition.

**Fig. 1:**
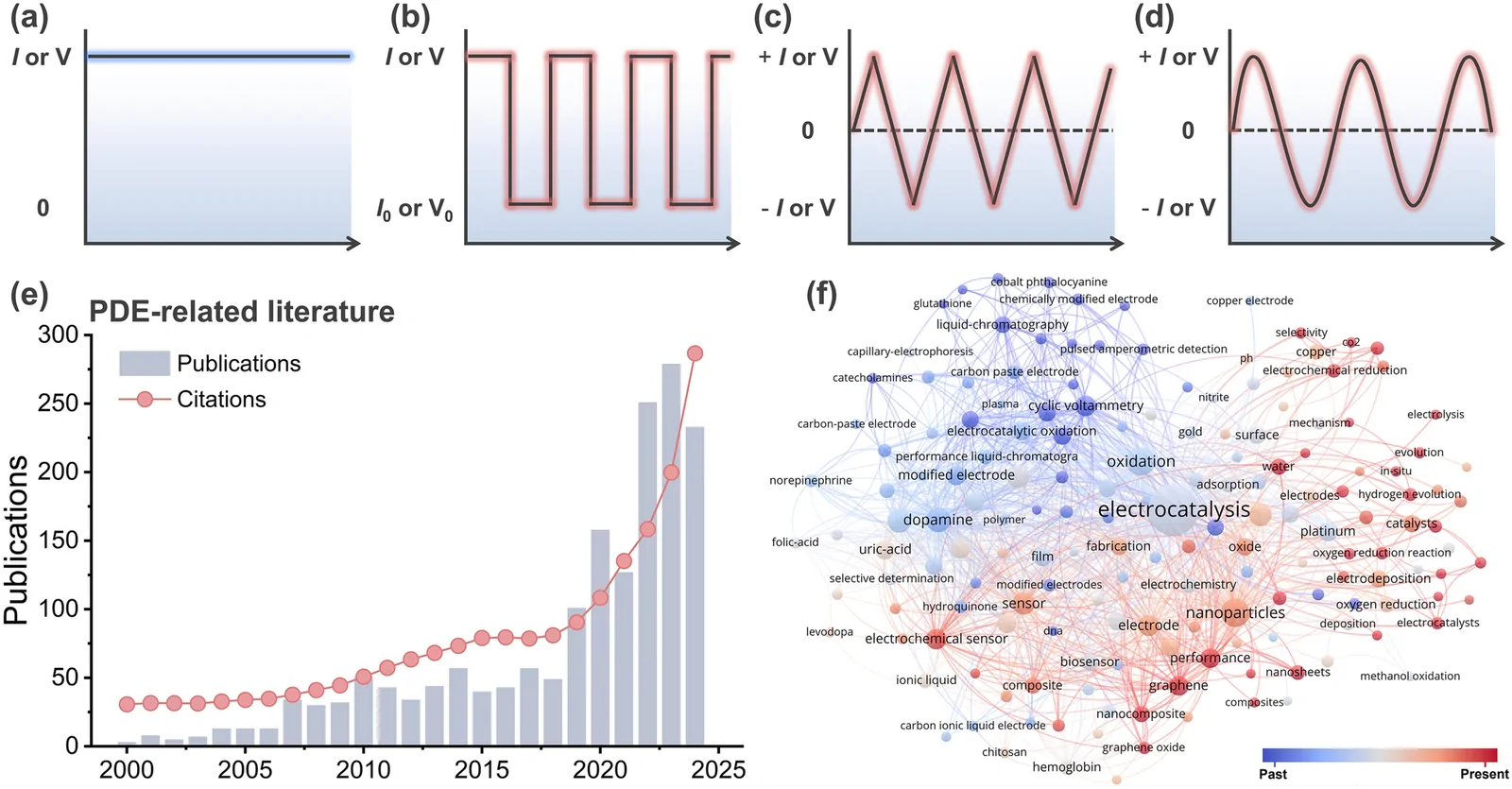
Common electrolysis waveforms used in PDE: **a** constant voltage or current, **b** square wave, **c** triangular wave, **d** sinusoidal wave. **e** Summary of the publication and citation distribution of PDE-related literature from 2000 to 2024. **f** Co-occurrence network of top keywords related to PDE analyzed using VOSviewer from 2014 to 2024. Data from Web of Science, May 2025

When electrochemical processes are operated under dynamic conditions, they are typically referred to as pulsed dynamic electrolysis (PDE). Pulsed electrochemical variables, such as voltage/current modulation, serve as effective experimental adjustment tools. By adjusting the relationship between interface factors (e.g., adsorption, desorption, surface reconstruction, and catalyst oxidation) and pulsed waveforms (e.g., pulsed duration and potential), PDE can affect the transient physicochemical equilibrium at the electrode/electrolyte interface, thereby significantly affecting the electrolysis results [18, 19]. Unlike conventional CE mode, PDE allows the current or voltage to vary with time, while CE electrolysis fixes the voltage value, which cannot be dynamically adjusted, as shown in Fig. 1a–d. In conventional CE electrolysis, electrochemical reactions at the electrode/solution interface proceed continuously, with reactants (such as H^+^ in WE) being consumed, and products (such as H_2_) being continuously generated. As a concentration gradient forms, reactants are replenished from the bulk phase of the solution to the electrode interface, while products detach from the catalytic sites and migrate toward the bulk phase [20, 21]. However, at higher current densities, the reactants at the electrode surface may be insufficiently supplied due to the rapid reaction rate, and the products may not detach from the surface in time [22]. This mismatch between the reaction and transport processes at the electrode/solution interface limits the efficiency of the electrochemical reaction.

**Fig. 2:**
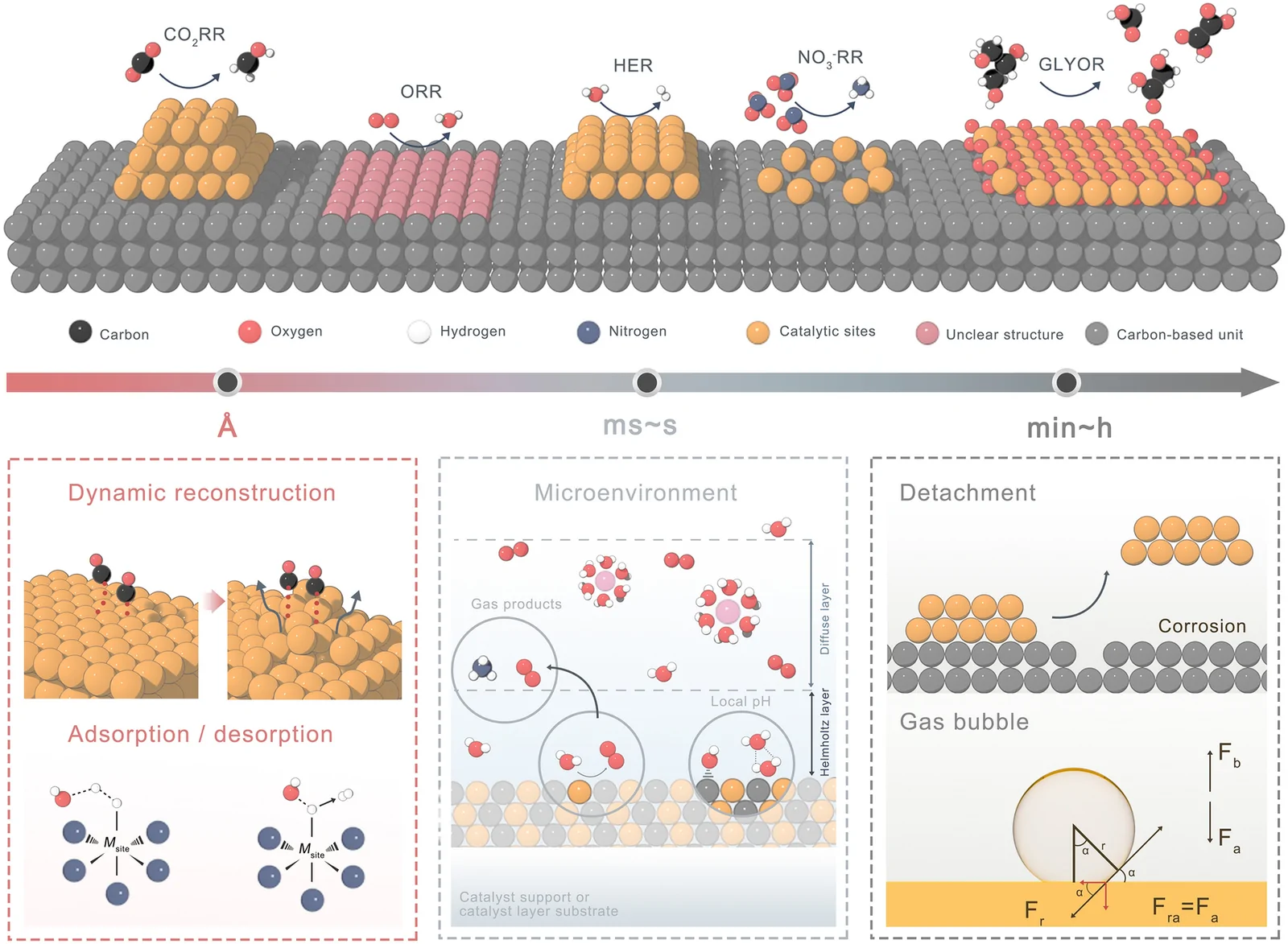
Dynamic physicochemical processes and multi-scale regulation mechanisms of PDE in different electrocatalytic reaction systems

PDE effectively alleviates this mismatch by adjusting multiple key parameters, including pulsed waveform (typically square waves), duty cycle, and frequency, thereby optimizing the reaction performance under different operating conditions [23]. The core principle and key aspect of PDE lie in the fact that during the “power-on” and “power-off” phases, the mass transfer process of reactants/products between the electrode interface and the solution is continuous, while the electrochemical reaction only occurs during the “power-on” phase. This creates ample space for synergistic effects and transport processes in the reaction, allowing for regulation across multiple time and length scales. Specifically, at the atomic scale, PDE affects the dynamic reconstruction of the electrode surface and the distribution of surface-adsorbed species. At the ms ~ s time scale, PDE affects the distribution of water, ions, and pH within the electric double layer (EDL). At the min ~ h time scale, PDE can effectively slow down the deactivation process of the electrode surface, thereby improving the durability of the electrode (Fig. 2). Therefore, as an efficient and cost-effective “plug-and-play” method, PDE can significantly affect the durability and selectivity of the reaction without changing the structure of the electrolysis cell or the electrode materials. This makes reaction systems in the laboratory more closely resemble the practical application of renewable energy fluctuations [24]. In view of the above advantages, there has been a growing interest in research on PDE in recent years, as shown in Fig. 1e, f. However, the large-scale application of PDE still faces challenges such as equipment compatibility, complexity in control system integration, and high cost investment, which require optimized strategies to achieve economically feasible “plug-and-play” deployment.

**Fig. 3:**
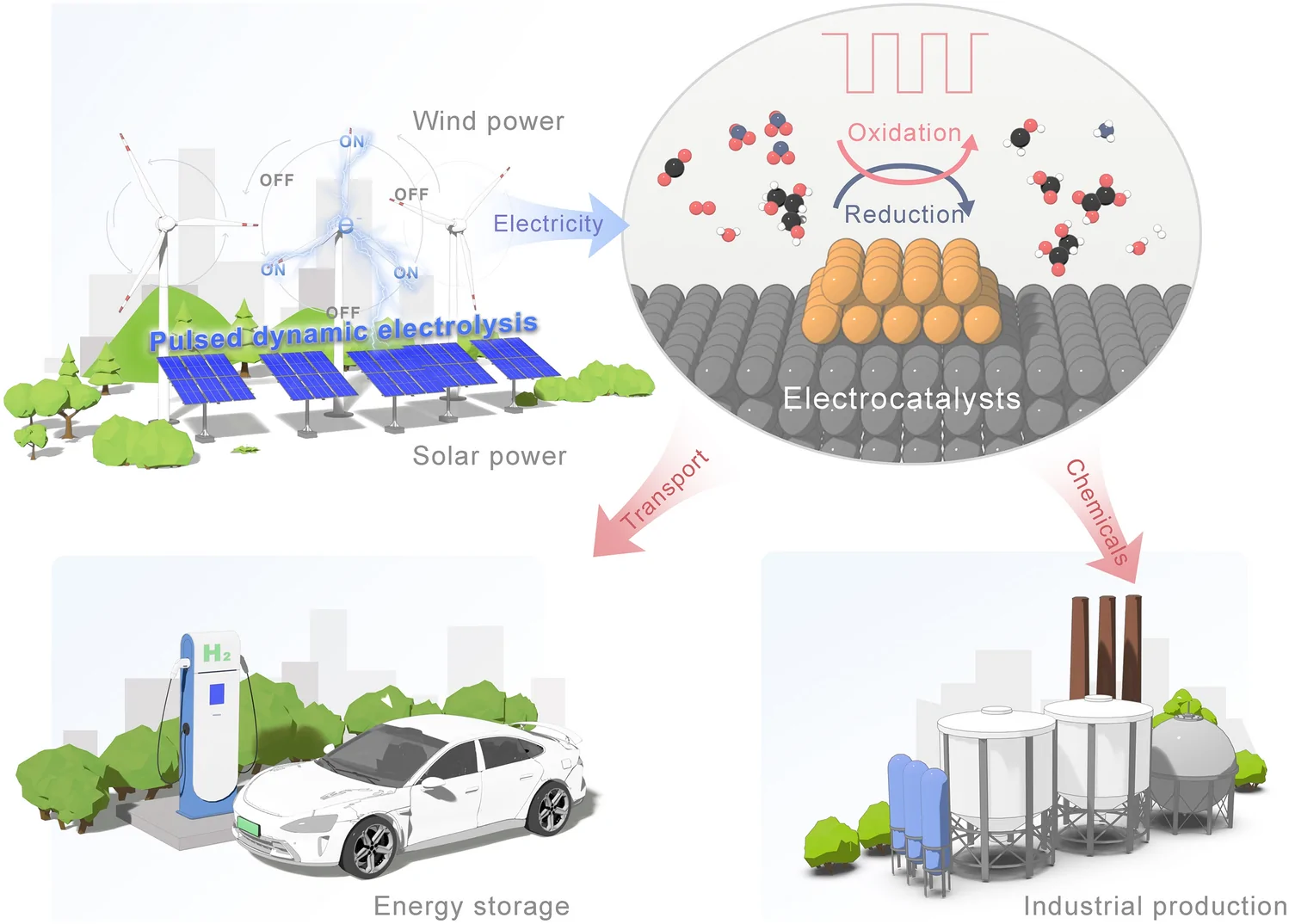
Prospects of renewable energy-driven PDE in energy conversion and low-carbon emissions. This includes the utilization of fluctuating wind-solar power for energy storage and industrial production

PDE, through cross-scale regulation of the kinetics of interface mass transfer and intermediate formation during reactions, is expected to profoundly affect the energy and mass transfer process, especially in terms of matching energy and mass flows. In recent years, although reviews have focused on the application of PDE in sustainable electrosynthesis [25], Cu-based catalysts for CO_2_ reduction reactions (CO_2_RR) [26], and wastewater treatment [27], most discussions have focused on the effect of PDE on catalysts and its application in electrochemical systems. However, the key regulatory mechanisms of PDE in energy and mass transfer and WE for hydrogen production have not received sufficient attention. Existing reviews have not comprehensively examined how pulsed parameters determine process outcomes, and have not fully clarified the unique contributions of PDE in energy and mass transfer, especially in terms of local microenvironment regulation, electrolysis system failure/passivation, and pulsed water electrolysis. This review comprehensively summarizes the latest efforts in understanding the mechanisms of PDE in energy and mass transfer, extending electrolysis system lifetime, and enhancing WE for hydrogen production. Special emphasis is placed on the role of rationally regulating PDE parameters to improve the energy efficiency of HER. Furthermore, this review uniquely integrates the latest research on hydrogen production from renewable energy with fluctuating power, providing new insights into the utilization of renewable energy generation and the industrial transformation of PDE (Fig. 3). The improvements in energy and mass transfer and hydrogen production performance regulated by PDE are mainly attributed to catalyst reconstruction and microenvironmental effects, including changes in catalyst valence and topological structure, intermediate adsorption/desorption, perturbation of the EDL, and local pH regulation, as well as effective inhibition of impurity deposition and optimization of diffusion layer and bubble escape processes. This review further elucidates how the frequency, duty cycle, and amplitude in PDE strategies affect key steps such as electron transfer, ion diffusion, and bubble detachment, thereby enhancing HER performance and revealing the complex interactions among these factors. Finally, the review summarizes the current challenges and opportunities in energy and mass transfer and enhanced hydrogen production, offering forward-looking perspectives to advance PDE from laboratory research to industrial applications, providing valuable insights for future research and practice.

## Effect on Local Microenvironment

### Intermediates Adsorption/Desorption

**Fig. 4:**
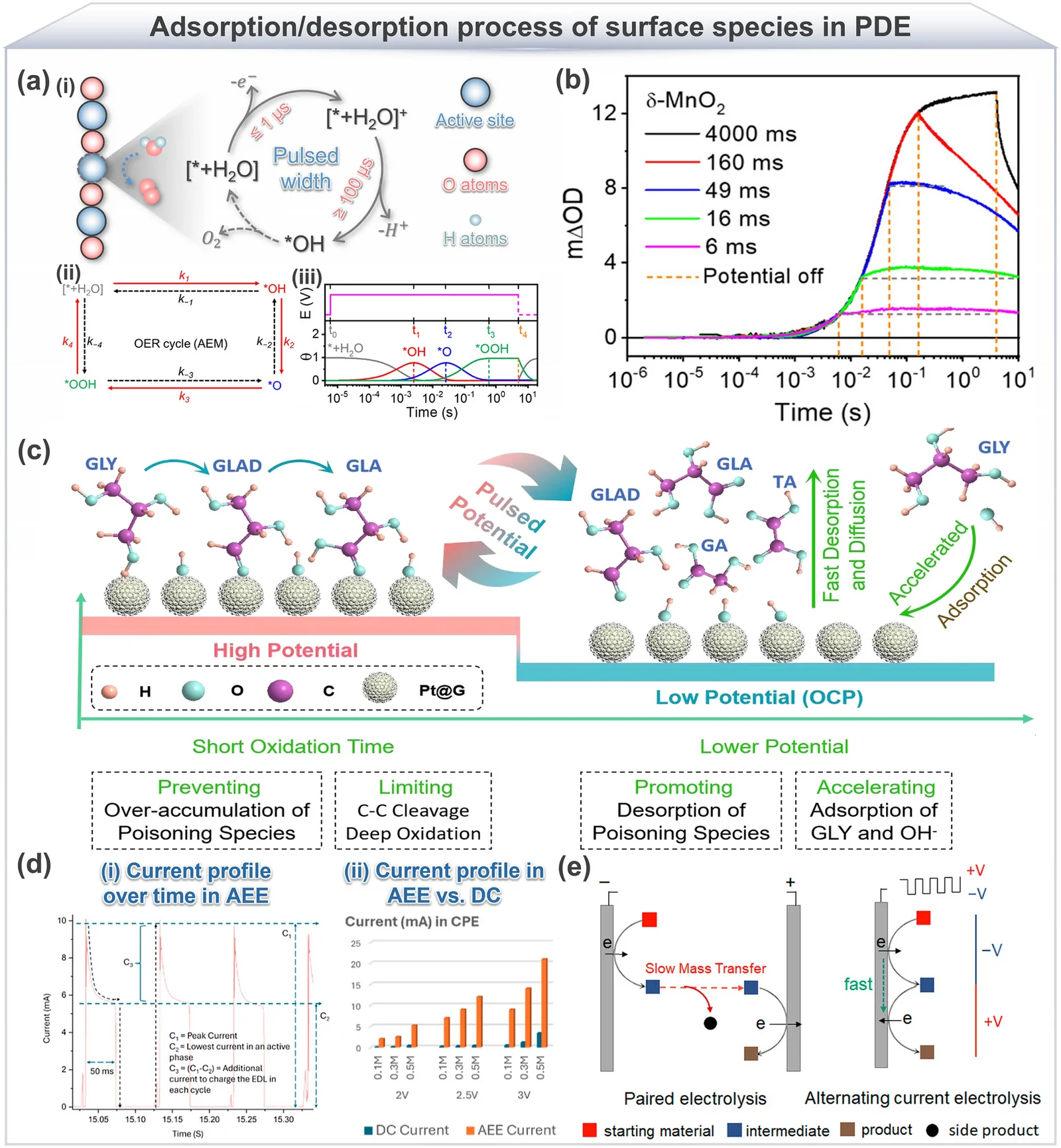
The adsorption/desorption process of surface species in PDE. **a** (i) Schematic diagram of the mechanism showing the effect of different pulsed widths on OER intermediates, (ii) OER cycle following the AEM mechanism, (iii) microkinetic simulation results of the time evolution of OER intermediate coverage at pulsed width of 5 s. **b** EC-TA curves of δ-MnO_2_ under different pulsed widths. Reproduced with permission from Ref. [30]. Copyright 2025, American Chemical Society. **c** Schematic diagram of the glycerol electro-oxidation reaction pathway based on PDE. Reproduced with permission from Ref. [37]. Copyright 2024, Springer Nature. **d** (i) Current–time curve in AEE, (ii) comparison of current–time curves between AEE and direct current (DC) electrolysis at different electrolyte concentrations. Reproduced with permission from Ref. [42]. Copyright 2024, American Chemical Society. **e** Sequential reactions achieved through electrode polarity switching for two opposite oxidation–reduction steps. Reproduced with permission from Ref. [43]. Copyright 2020, American Chemical Society

In the WE process, revealing the dynamic evolution of intermediates is crucial for establishing the structure–activity relationships between catalyst structure and reaction performance. However, the formation and transformation of intermediates typically occur on extremely short timescales (μs ~ s) and involve complex electron-proton-coupled mechanisms, a characteristic also present in both the oxygen evolution reaction (OER) and HER [28, 29]. Conventional steady-state electrochemical and spectroscopic techniques often struggle to achieve high temporal resolution and sensitivity for tracking such processes. Recently, Wei et al. developed an in situ electrochemical transient absorption spectroscopy technique, using pulsed potential as the excitation source, in which different pulsed widths (ms ~ s) are applied while monitoring absorbance changes in real time [30]. Based on the adsorbate evolution mechanism of MnO_x_ catalysts, this approach enabled dynamic analysis of the formation and consumption processes of key OER intermediates such as *OH. By combining these results with microkinetic simulations, the authors identified the reaction stages corresponding to different time windows. Short pulses (pulsed width < 2.5 ms) reflect the formation kinetics of *OH species, whereas long pulses (pulsed width > 500 ms) correspond to rate information for the rate-determining step of the OER (Fig. 4a). More importantly, experimental observations revealed that even after the potential was switched off (to open-circuit potential, OCP), the generation of *OH intermediates continued, uncovering the asynchrony between electron and proton transfer, where the electron transfer rate is faster than the proton transfer rate (Fig. 4b). Notably, the kinetics of the first OER intermediate *OH play a critical role throughout the entire OER cycle. This finding provides direct experimental evidence for the true formation pathway of OER intermediates.

PDE drives changes in the catalyst surface topology, causing surface atoms to interact with reaction intermediates, altering the catalyst's interface energy and promoting changes in the catalyst [31]. For example, Lee et al. demonstrated the structural evolution of the near-surface region of polycrystalline copper electrodes under in situ CO_2_ reduction conditions [32]. Due to the strong surface binding of *CO and *H intermediates, which is sufficient to move surface atoms, in situ changes were observed on the polycrystalline and nanocrystalline copper surfaces. Kimura et al. studied the changes in the electrode surface during electrochemical CO_2_RR under pulsed potentials [33]. The enhanced CO_2_ reduction selectivity under pulsed potential was not due to changes in electrode composition, but rather changes in surface adsorption species. Under pulsed potential, the adsorption of OH^−^ on the electrode surface was promoted, inhibiting the HER, while also promoting CO adsorption on the Cu top sites, preventing catalyst deactivation caused by CO adsorption on the Cu bridge sites.

PDE optimizes the adsorption of reaction intermediates such as *H or *CO in CO_2_RR, thereby altering the interface free energy of copper surfaces (111), (100), and (110). This optimization ultimately results in higher activity and selectivity for CO_2_RR under pulsed conditions [34]. Interestingly, the interactions between the optimized surface Cu atoms and CO_2_RR intermediates (e.g., *CO, *H, *OH, etc.) are thought to overcome the kinetic barriers of surface reconstruction. Furthermore, Hu et al. demonstrated by in situ electrochemical mass spectrometry and in situ infrared spectroscopy that PDE increases the local surface concentrations of *NH_2_ and *CO intermediates while inhibiting the HER, thus promoting C−N coupling and urea formation [35]. PDE can efficiently regulate the *CO reduction pathway by increasing the concentration of specific anions on the copper surface. Wang et al. found that by applying periodic positive pulsed potentials to the cathode and using KF, KCl, and KHCO_3_ as electrolytes, the concentration of specific anions such as F^−^, Cl^−^, and HCO_3_^−^ near the electrode was enhanced [36]. In this way, PDE periodically regenerates Cu(I) states in CO_2_RR, significantly improving the FE of CO (53% ± 2.5%), C_2+_ (76.6% ± 2.1%), and CH_4_ (42.6% ± 2.1%). Chen et al. used PDE strategy for selective electrocatalytic oxidation of glycerol to glyceric acid on Pt-based catalysts [37]. They found that the application of pulsed potentials prevented excessive oxidation of the catalyst, and by adjusting the adsorption and desorption behaviors of surface species, it prevented the overaccumulation of poisoning intermediates, allowing active sites to re-adsorb *OH and glycerol. Compared to conventional CE, this PDE strategy significantly improved the selectivity for glyceric acid (from 37.8% to 81.8%), as shown in Fig. 4c. Specifically, during the short pulsed width high-potential phase, the substrate GLY is oxidized to generate intermediates, whereas the low-potential phase facilitates the rapid desorption and diffusion of target intermediates (e.g., GLA), preventing their over-oxidation or conversion into byproducts. At the same time, the low potential helps refresh the active sites on the catalyst surface, promoting the re-adsorption of GLY and mitigating catalyst deactivation caused by the prolonged presence of poisoning intermediates (e.g., glyceraldehyde and GLAD). In summary, PDE precisely regulates the duty cycle and frequency between high and low potentials to control the residence time of intermediates on the catalyst surface, thereby regulating the target reaction pathway and product selectivity, while also extending catalyst’s lifespan.

Furthermore, Woldu et al. found that introducing sulfur vacancies into SnS_2_ (Vs-SnS_2_) significantly enhances the electrocatalytic activity for CO_2_RR [38]. However, during CE, Vs-SnS_2_ exhibited an extremely low-energy barrier for the formation of *H intermediates, which covered the active sites of the catalyst and prevented the formation of carbon-based intermediates, leading to H_2_ evolution with almost no CO production. In contrast, during PDE, SnS_2_ nanosheets dynamically reconstructed into partially oxidized SnS_2-*x*_, where the oxidized phase played a key role in enhancing the conversion of CO_2_RR to C_1_ products. The oxidized phase selectively generated formate, while the sulfur vacancies promoted H_2_ production. As a result, the selectivity for C_1_ products improved from ~ 60% under static potential to ~ 93% under pulsed potential electrolysis. PDE can dynamically stabilize the residual oxygen atoms in the oxide catalyst during the electrochemical CO_2_RR. Mao et al., through experiments and simulations, discovered that the residual oxygen atoms in oxide catalysts effectively regulate the adsorption of reaction intermediates, increasing the surface concentration of the key intermediate CO_2,ads_ from 5e^2^ to 1e^4^ and enhancing the current density for CO_2_ reduction. Moreover, during the PDE process, when applying the potential *E*_a_, a high concentration of electronegative oxygen (E − O) was maintained on the Ag electrode, which enhanced the coverage of CO_2,ads_ on the Ag surface [39]. When the potential switched to *E*_c_, the high coverage of CO_2,ads_ on the Ag electrode promoted the reduction of CO_2_ to CO, resulting in FE of 96.6% for CO production at −0.7 V vs. RHE. The catalyst maintained 90% stability after 100 h of testing.

PDE is not only applicable to Cu-based catalysts [40], it is an effective experimental method that provides new opportunities for regulating product selectivity, catalyst reconstruction into active species, and enhancing long-term catalyst stability. By adjusting crystal faces and the chemical environment, the effects of pulsed potentials on catalyst morphology and molecular adsorption/desorption can be explored in depth [41].

Another form of PDE, the periodic electrode polarity switching, shows benefits when applied to reactions involving short-lived intermediates. Bera et al. reported an alternating electrode electrolysis (AEE), which features the use of two pairs of cathode–anode for electrosynthesis, successfully applied to three-component reactions for the synthesis of ketone-functionalized 1,4-quinones from aryl diazonium salts, 1,4-quinones, and acetone [42] (Fig. 4d). Notably, compared to conventional electrolysis methods, AEE utilizes the advantages of PDE without the need for resting phases, thereby rapidly utilizing photo-activated short-lived intermediates. The pulsed method of alternating current electrolysis (ACE) can also effectively overcome the low yield issue caused by the slow transfer of short-lived intermediates between electrodes. Rodrigo et al. demonstrated in an ACE process, where applying an alternating voltage (± V) allowed substrates to undergo consecutive oxidation and reduction transformations on the same electrode [43] (Fig. 4e). Using the trifluoromethylation of (hetero)arenes as a model reaction, the results showed that the reaction yield significantly improved from 13% in traditional paired electrolysis to 84% in ACE.

### Local pH

The effect of PDE on local pH primarily arises from the electron transfer in electrochemical reactions and the subsequent ion exchange, particularly the generation or consumption of H^+^ and OH^−^. During the reduction and oxidation cycles, the gain or loss of electrons at the electrode promotes the reduction and oxidation of local species, accompanied by changes in the local microenvironment pH. By adjusting the pulsed amplitude, frequency, and duty cycle, the local pH of the target reaction can be controlled to create a favorable environment, thereby enhancing the FE and stability of the target product.

**Fig. 5:**
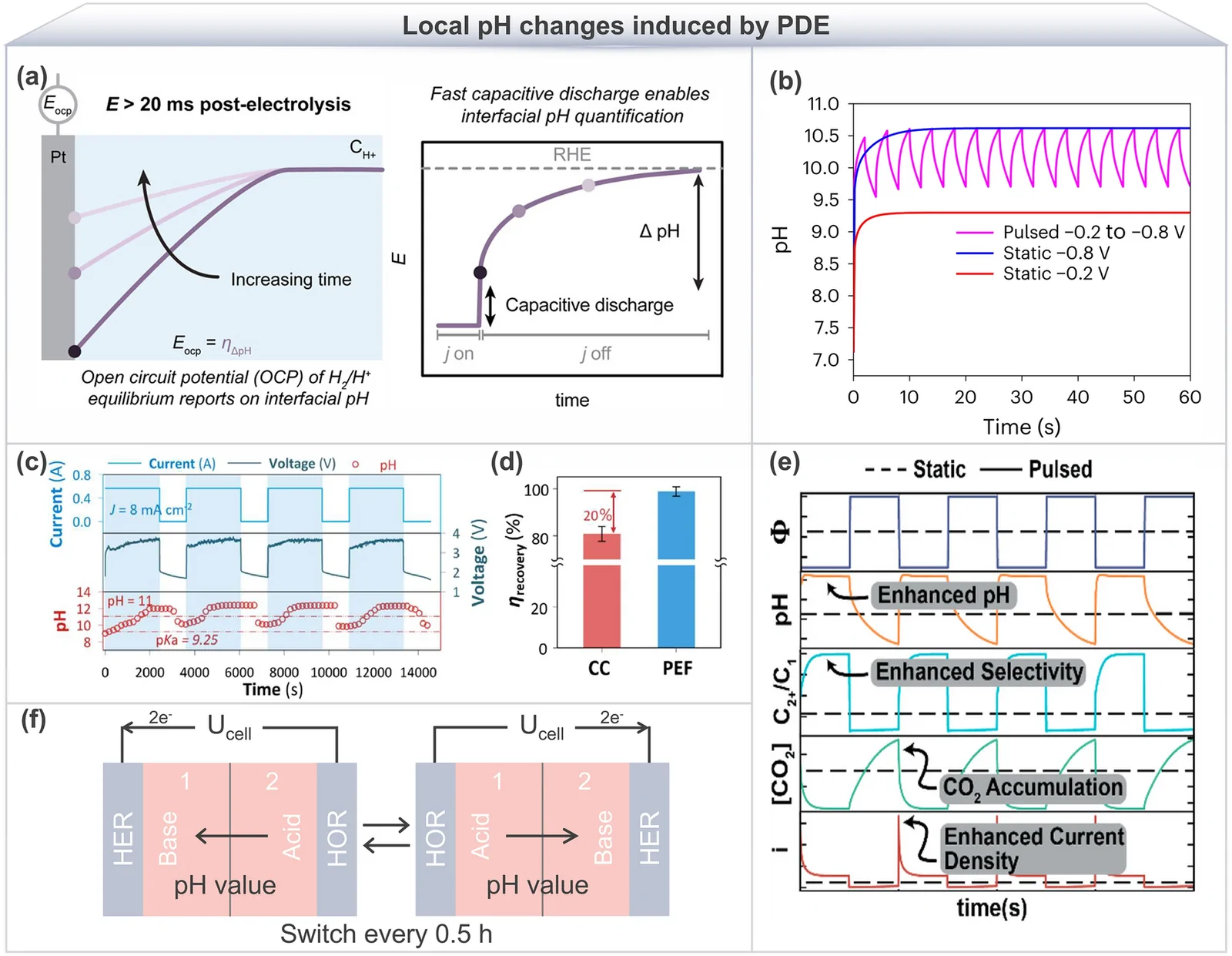
Local pH changes induced by PDE. **a** pH gradient determined by rapid capacitive discharge induced by PDE. Reproduced with permission from Ref. [44]. Copyright 2024, Elsevier. **b** pH distribution under PDE and CE conditions in the boundary layer at distance of 100 µm (*t*_a_ = *t*_c_ = 2 s). Reproduced with permission from Ref. [35]. Copyright 2024, Springer Nature. **c** Schematic diagram showing the changes in current, voltage, and cathode pH with time under the PDE strategy. **d** Ammonia recovery rate under CE and PDE strategies. Reproduced with permission from Ref. [46]. Copyright 2024, Elsevier. **e** PDE causes a transient state with higher pH and CO_2_ concentrations near the catalyst surface, enhancing C_2+_ product selectivity. Reproduced with permission from Ref. [47]. Copyright 2021, American Chemical Society. **f** Schematic diagram of seawater alkalization process under polarity reversal

During PDE, the pH at the electrode interface usually differs significantly from the bulk solution. Understanding the pH variations induced at the interface by PDE is crucial for gaining deeper insights into the mechanisms of pulsed regulation. Sauvé et al. quantified polarization-induced pH fluctuations at the interface by measuring the OCP based on the H_2_/H^+^ equilibrium on the platinum surface. The study found that, in strongly buffered solutions, pH fluctuations can exceed 2 units at a moderate current density of −30 mA cm^−2^ [44] (Fig. 5a). Further analysis revealed that the supporting electrolyte plays a key role in the pH changes during polarization. The addition of alkali metal cations led to pH fluctuations exceeding 13 units in the bulk acidic electrolyte, a phenomenon more pronounced than in electrolytes containing only H^+^. Moreover, when a Nafion ion exchange membrane layer was introduced, the pH fluctuations induced by polarization were further enhanced, causing pH fluctuations to exceed 12 units. This phenomenon was observed in both buffered electrolytes and electrolytes containing additional supporting electrolytes. In CO_2_RR, Jeon et al. found that PDE could effectively alter product selectivity. When the anode potential (*E*_an_) was 1.2 V, PDE exhibited a high CH_4_ selectivity of up to 48.3%, compared to only 0.1% under CE. When *E*_an_ was 0.9 V, PDE showed a higher selectivity for C_2_ products [45]. Their in situ spectroscopy (XAS, SERS) combined with ex situ electron microscopy (SEM, TEM) characterization indicated that when *E*_an_ was 1.2 V, PDE led to the consumption of OH^−^ near the catalyst surface, creating a low OH^−^ environment favorable for CH_4_ formation. The enhanced CH_4_ selectivity could be attributed to the pH effect. Interestingly, during the periodic application of pulsed potentials in CO_2_RR, the pulsed anodic potential disrupts the steric hindrance of cations in the outer Helmholtz plane and replaces H-adsorbates with OH-adsorbates, causing the accumulation of OH^−^adsorbates. This creates a higher pH surface environment, promoting CO adsorption and suppressing the HER [33].

PDE has shown significant advantages in regulating local reaction environments, making it widely used in enhancing FE and optimizing reaction performance. Hu et al. used PDE to significantly improve the FE of urea synthesis while effectively suppressing the increase in local pH. Further simulations revealed the relationship between local pH, distance, and time [35] (Fig. 5b). The study showed that under co-reduction conditions at -0.8 V vs. RHE, the local pH under PDE is lower than that under CE, validating the effectiveness of PDE in regulating local pH. Moreover, in wastewater treatment, the research by He et al. further expanded the potential of PDE in improving recovery efficiency. By comparing CE and PDE strategies, they found that under the same energy input conditions, PDE significantly improved the conversion and extraction conditions of ammonia (pH > 11) by adjusting pH. Although the η_removal_ under the operating condition of average removal rate of 37.5 g-N m^−2^ h^−1^ showed little difference between the two strategies, the ammonia η_recovery_ under PDE increased significantly from 80.9% to 98.8%, while the recovery rate under the CC mode remained at 80.9% [46] (Fig. 5c, d). This indicates that PDE reduces overpotential losses by adjusting the pH, thereby enhancing overall recovery efficiency. Additionally, the research by Bui et al., based on Cu catalysts and CsHCO_3_ electrolytes, further revealed the promoting effect of PDE on complex electrocatalytic reactions [47]. By establishing a time-dependent pulsed CO_2_ electrolysis model (Fig. 5e), they found that PDE induces dynamic changes in pH and CO_2_ concentration near the catalyst surface. The optimized pulsed waveform is maintained at a more negative potential (~ -1.55 V vs. RHE) and cycles through transient high pH and high CO_2_ concentration states, significantly improving the FE of C_2+_ products. More importantly, He et al. demonstrated a new application of PDE in organic synthesis, further enriching its potential uses [48]. They used PDE to reduce NO_2_^−^ to NH_3_ and subsequently couple NH_3_ with arylboronic acid to form arylamines. The study found that PDE slows down pH changes, promotes the concentration increase of key species near the electrode surface, and leads to the consumption of OH^−^ near the cathode surface, thereby suppressing the generation of side reactions, increasing the selectivity of arylamines, and accelerating C−N bond formation. Meanwhile, Guan et al. discovered that the electrochemical pH oscillation strategy induced by pulsed current is an efficient CO_2_ capture method [49]. They used a pulsed current strategy (Fig. 5f) that alternates current direction every 30 min to achieve periodic pH fluctuations. Seawater with an initial pH of 7.92 was transformed into either alkaline (pH ≈ 10) or acidic (pH ≈ 2) solutions, depending on the reaction type, with minimal time delay. This demonstrates that the PDE strategy enables rapid switching between acidic and alkaline environments in different chambers, making it possible to meet the reaction requirements in various environmental conditions. Furthermore, the pH regulation mechanism of PDE at the interface was further validated in electrocatalytic reactions. Ye et al. used a 30 μm diffusion layer model to simulate the pH variation at the cathode interface under PDE. They found that, compared to CE, applying pulsed cathodic potential (*E*_c_) induced a pH increase of approximately 0.1 units in the local region [50]. This slight pH change partially inhibited the HER in CO_2_RR, helping to improve the selectivity of the electrocatalytic reaction. This further demonstrates the unique role of PDE in dynamically regulating interface pH, thereby affecting charge transfer during the reaction process.

### Interfacial Species Concentration

PDE significantly affects the charge distribution and electric field intensity on the electrode surface. This change affects the adsorption and desorption behavior of ions on the electrode surface, as well as the migration rate and diffusion behavior of ions in the solution. Due to the periodic variation of the pulsed voltage, the dynamic disturbance of the EDL at the electrode/solution interface promotes the diffusion of ions and molecules, thereby reducing concentration polarization in electrochemical reactions. Consequently, PDE has the potential to enhance the reaction kinetics at the electrode/solution interface, increase the local concentration of reactants or intermediates, reduce concentration polarization at the electrode interface, and ultimately improve the efficiency and selectivity of electrochemical reactions.

**Fig. 6:**
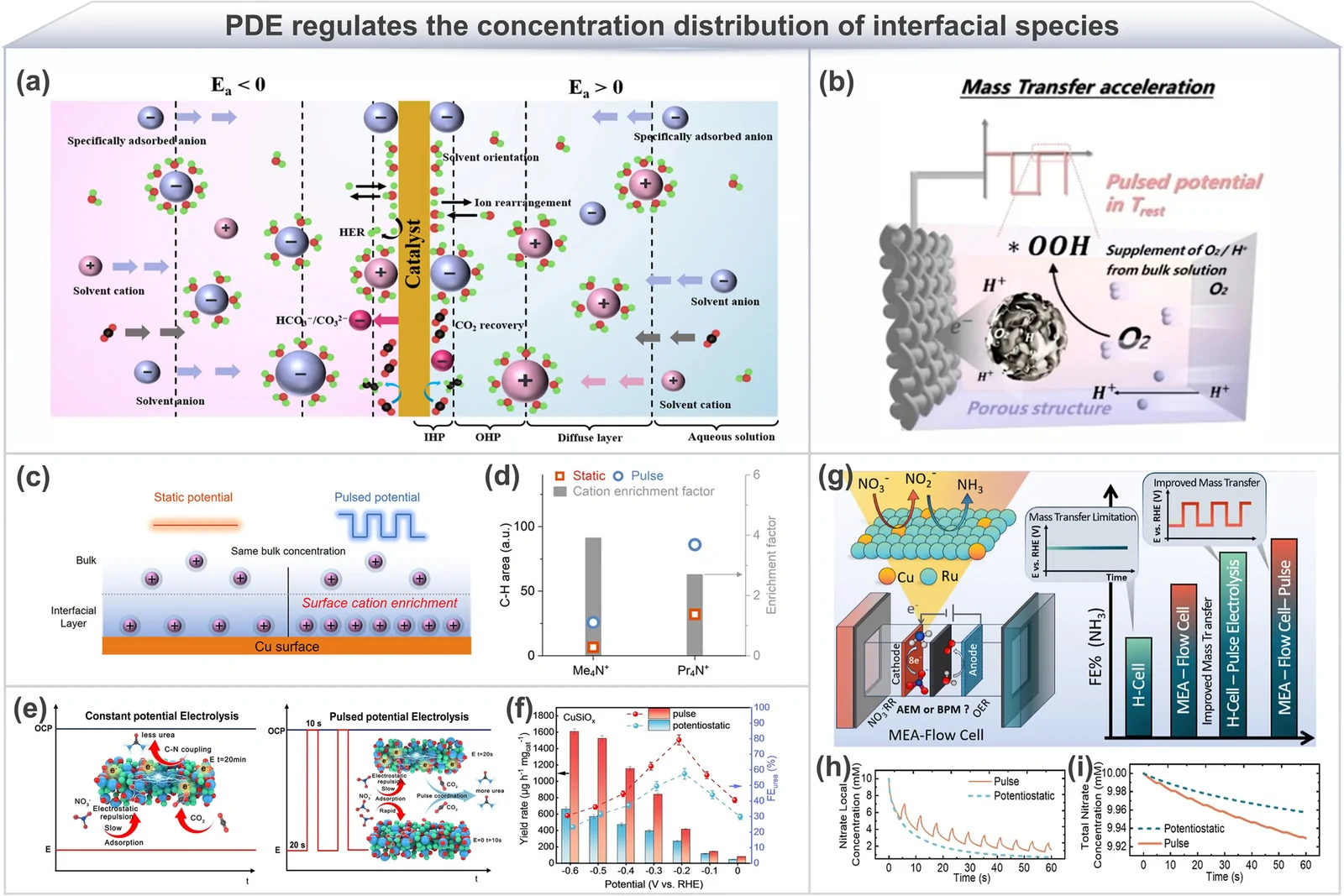
PDE regulates the concentration distribution of interfacial species. **a** Dynamic regulation of the EDL solid/liquid interface in CO_2_RR. Reproduced with permission from Ref. [26]. Copyright 2024, American Chemical Society. **b** Acceleration mechanism of mass transfer in the diffusion layer during the Toff period. Reproduced with permission from Ref. [53]. Copyright 2023, American Chemical Society. **c** Schematic diagram of cation enrichment induced by pulsed potential. **d** Raman peak area of CO_2_RR under CE and PDE conditions in Me_4_N^+^ and Pr_4_N^+^ electrolytes, and the ratio of their peak areas, representing the cation enrichment factor (gray bar chart). Reproduced with permission from Ref. [54]. Copyright 2024, American Chemical Society. **e** Schematic diagram of CE and PDE, with inset illustrating the reaction mechanism for increasing the concentration of active species. **f** FE and urea yield of CuSiO_x_ under different CE and PDE. Reproduced with permission from Ref. [58]. Copyright 2024, Wiley–VCH. **g** Schematic of PDE enhanced NO_3_^−^RR for ammonia production in MEA assembly structures. **h** Simulated local concentration of NO_3_^−^ and **i** total nitrate ion concentration in solution as a function of time during PDE and CE processes. Reproduced with permission from Ref. [59]. Copyright 2024, American Chemical Society

The EDL plays a critical role in determining reaction kinetics and the distribution of intermediates/products, with the dynamic regulation of the EDL structure by PDE being particularly essential. Xi et al. demonstrated that applying a pulsed potential in CO_2_RR can reduce the adsorption and periodic displacement of H^+^ in the inner Helmholtz plane (IHP), enrich anions such as OH^−^ and Cl^−^, and increase the local pH, thereby inhibiting HER and promoting the formation of C_2+_ products [26]. Moreover, PDE optimizes the charge transfer process by altering the surface charge density of the electrode and reorienting the dipoles of water molecules, thereby improving selectivity and efficiency (Fig. 6a). In addition, due to its ability to perturb the EDL structure and excite water molecule behavior, PDE has emerged as an important research direction for enhancing WE efficiency. Burton et al. employed a method based on detecting changes in the electrolyte capacitance to monitor the dynamic behavior of water molecules during WE [9]. They reported that pulsed electric fields can regulate energy flow, mitigate excessive accumulation in the EDL, and enhance the orientational polarization and excited-state energy of water molecules, thereby significantly influencing interfacial reaction processes. Water molecules in high-energy states are not only more likely to break hydrogen bonds with the electrode surface but can also disrupt the long-chain hydrogen bond network within the EDL, thereby promoting intermolecular migration and improving energy transfer efficiency. Particularly under high-frequency pulsed conditions, the system exhibits lower electrochemical impedance and higher interfacial activity, creating a micro-reaction environment superior to that of direct current (DC) power supply. Thus, the core of PDE’s efficiency enhancement lies in achieving synergistic regulation of energy transfer, molecular behavior, and electrochemical dynamics.

Further research has shown that the dynamic perturbation caused by pulsed voltage has a significant effect on the electrode/solution interface. Xin et al., using in situ laser scanning confocal microscopy, demonstrated that PDE promotes the diffusion of ions and species, reducing concentration polarization [51]. During the *E*_off_ period, H^+^ rapidly diffuses from the bulk solution to the interface, replenishing ions consumed in the reaction and regulating the consumption/replenishment of reactants and the generation/desorption of charged products, significantly enhancing reaction kinetics. The effect of PDE has been further validated in specific reaction systems. Huang et al. found that in nitrate reduction reactions (NO_3_^−^RR), PDE increased the local concentration of NO_3_^−^ at the cathode while promoting the diffusion of NH_4_^+^, thereby alleviating mass transfer limitations. The apparent rate constant calculated under CE conditions was 0.00197 min^−1^, while under the PDE strategy, it increased to 0.00491 min^−1^ [52]. Further indicating that PDE improves reaction kinetics by regulating interfacial ion diffusion. Additionally, Ding et al. showed that PDE accelerates interfacial mass transfer in 2e^−^ORR, especially for catalysts with hierarchical pore structures, where PDE exhibits significant synergistic catalytic effects, improving the transfer efficiency of oxygen and protons from the solution to the interface [53] (Fig. 6b). PDE precisely regulates activity and selectivity in the rate-determining steps of oxygen adsorption and protonation in ORR on carbon-based catalysts. The regulation of ion distribution and reactant enrichment by pulsed potential has also garnered attention. Li et al. used different alkali metal cations to show that the enrichment effect of PDE on cations is closely related to the hydrated ionic radius [54], and the smaller the ion, the stronger the enrichment effect (Fig. 6c, d).

In our previous work, we designed experiments to accelerate proton enrichment at the electrode/solution interface from the perspective of reaction kinetics, focusing on how PDE promotes the diffusion and mass transfer of reactant ions [55]. These studies further revealed the enhancement mechanism of PDE in proton exchange membrane water electrolysis (PEMWE) for hydrogen production. Under CE, H^+^ in the solution near the electrode surface is rapidly consumed. However, under PDE, H^+^ is replenished during the *E*_off_ phase. PDE accelerates proton enrichment at the electrode/solution interface, thereby enhancing PEMWE hydrogen production. In addition, during studies on PDE coupled anodic oxidation reactions to assist hydrogen production, we found that PDE increases the local concentration of SO_3_^2−^ anions at the anode, reducing concentration polarization effects at the electrode and altering the charge transfer behavior of the EDL [56]. Matching the pulsed potential and frequency induces changes in the charge transfer process, thereby enhancing the coupling between protons and transferred electrons on the electrode surface. Compared to CE, PDE increases the total charge transferred on the electrode surface by up to 12.87%, further boosting the Faradaic charge in the electrocatalytic process. The potential of PDE for enhancing complex reactions, such as C–N coupling between CO_2_ and NO_3_^−^ [57], has been significantly demonstrated. Qiu et al. achieved efficient urea synthesis at Cu–O–Si interface sites with anti-reconstruction properties by periodically renewing the concentration of active species in the nernst diffusion layer [58]. This overcame electrostatic interactions and increased the concentration of anionic reactants and active intermediates near the working electrode (Fig. 6e). As shown in Fig. 6f, under optimal pulsed operation conditions, the Cu–O–Si interface achieved an exceptional urea production rate of 1606.1 μg h^−1^ mg^−1^, with high selectivity (79.01%) and stability (FE remained at 80% even after 80 h of testing). In the wide application of PDE to regulate the concentration of ions at the interface and improve the performance of complex reactions, Boppella et al.’s research further revealed its potential in overcoming mass transfer limitations and increasing reaction rates [59]. From the perspective of reactor engineering and electrochemical analysis, they addressed the mass transfer bottleneck caused by low concentrations of NO_3_^−^ in H cells using PDE and Cu_x_Ru_y_ alloy catalysts (Fig. 6g). As shown in Fig. 6h, PDE alternately enhances nitrate ion enrichment at the cathode surface via anodic potential modulation, significantly increasing the local concentration and improving the mass transfer efficiency of the electrolytic cell. Despite the significant increase in local concentration, PDE also accelerates the removal of acylates in the solution (Fig. 6i). Consequently, PDE significantly reduces side reactions such as HER, increasing NH_3_ yield by up to 3.75 times and doubling the FE, showcasing remarkable application potential.

In summary, while PDE shows great potential in enhancing reaction kinetics, optimizing mass transfer, and regulating the interfacial environment, systematic theoretical guidance for optimizing pulsed parameters (e.g., frequency, duty cycle, and amplitude) is still lacking. Extensive experimental validation is required to identify optimal conditions for different reaction systems. Moreover, the potential of PDE when coupled with novel catalysts and membrane materials remains underexplored, and further investigation into related mechanisms is needed. In the future, advanced characterization techniques can be combined with multi-scale modeling to deeply analyze interfacial kinetics and mass transport processes, and to achieve efficient and tunable PDE.

## Effect on Electrode Degradation

### Electrocatalyst

Typically, surface reconstruction is driven by the thermodynamic requirement to minimize surface free energy [60], but under environmental conditions, significant kinetic barriers hinder this process. The rate of surface reconstruction depends on the mobility of surface atoms. As an inverse measure of surface atomic mobility, the cohesive energy of copper (336 kJ mol^−1^) is lower than that of platinum (546 kJ mol^−1^), making it a surmountable obstacle under operating potentials [61]. The surface topological reconstruction of the catalyst emphasizes that changes in surface nanostructure can occur even in the absence of redox processes. This includes the reconstruction of surface roughness, porosity, crystallinity, and crystal planes [62]. The interaction between surface atoms of the catalyst and reaction intermediates can alter the interfacial energy of the catalyst, driving structural changes.

The application of PDE has gradually revealed its unique advantages in catalyst surface reconstruction and reaction regulation. PDE offers the ability to achieve in situ reconstruction on catalyst surfaces, such as controlling oxidation states and disturbing the EDL, enabling highly efficient electrocatalysis. Under PDE conditions, the structural reconstruction of catalysts primarily involves two aspects: changes in oxidation states and surface topology. The oxidation state changes of catalysts include the reduction/oxidation reactions of electrocatalysts under applied potentials, leading to alterations in composition and phases. For example, during the CO_2_RR, copper oxides are reduced to metallic copper with a restructured nanostructure [63]. In alkaline HER, nickel is easily oxidized to hydroxides. These processes modify the chemical composition and crystal structure of the catalyst. Further exploration of the dynamic changes in catalyst oxidation states can help reveal the regulatory mechanisms of PDE. Jeon et al. proposed a pulsed CO_2_RR strategy involving short pulses at anodic oxidation potentials followed by short pulses at cathodic reduction potentials, repeated in cycles. By appropriately selecting the *E*_an_, different amounts of Cu(I) could be regenerated under the pulsed CO_2_RR conditions, significantly increasing the production of C_2+_ products [45]. Interestingly, the enhanced ethanol yield was associated with the coexistence of Cu(I) and Cu(0), while the ethylene yield depended on the length of Cu(100) steps. To further reveal the dynamic oxidation state changes of Cu during pulsed CO_2_ electrolysis, attention should be given to the mismatch between the spectral acquisition time scale and the pulsed conditions. It can be seen that the potential of PDE in regulating catalyst oxidation states and optimizing reaction pathways is continuously being explored.

When the active sites of a catalyst undergo chemical or physical changes, resulting in decreased product selectivity or production rates, the catalyst is considered to have deactivated. In flow cell, two of the most common degradation mechanisms in gas diffusion electrodes (GDEs) are surface reconstruction of the catalyst and the leaching or detachment of active catalyst materials from the gas diffusion layer (GDL). Generally, most catalysts possess specific compositions and structures designed to achieve excellent activity and selectivity for the target reactions. However, during electrolysis, surface polarization of the catalyst and its subsequent interactions with reactants, intermediate species, and the conductive substrate can lead to structural transformations of the catalyst. In the field of CO_2_RR, Huang et al. studied copper nanocubes of different sizes and revealed a unique and prevalent degradation mechanism for Cu-based catalysts during CO_2_RR [34]. By tracking the morphological evolution of the catalyst during electrolysis, they found that potential-driven nanoparticle aggregation was the primary degradation pathway. Additionally, PDE strategies may cause the migration of copper particles between the electrocatalyst and the GDL, leading to their reattachment on the GDL and the formation of new active sites in the GDE. This results in a decline in CO_2_RR activity and the occurrence of HER side reactions.

**Fig. 7:**
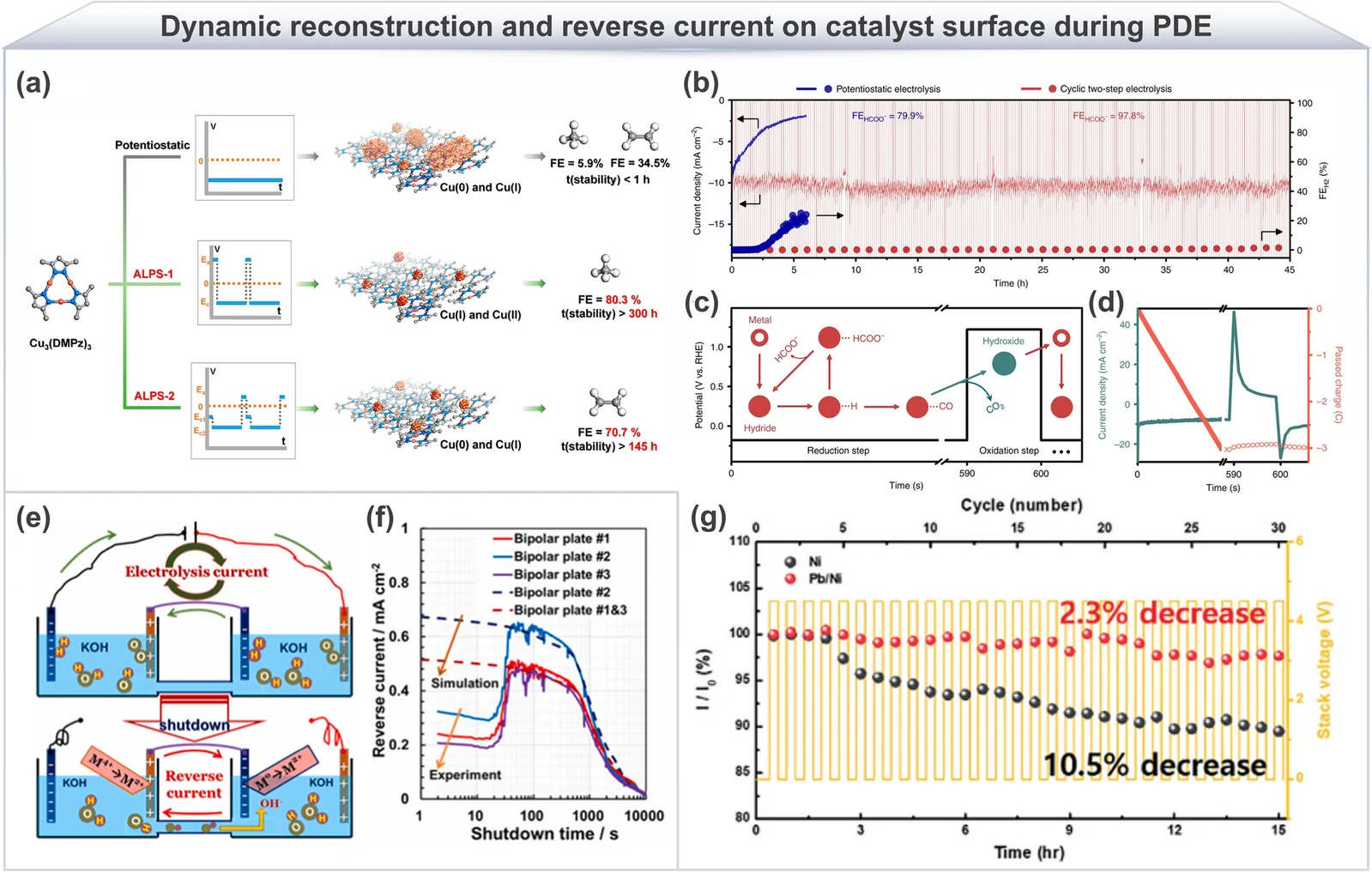
Dynamic reconstruction and reverse current on the catalyst surface during PDE. **a** Performance of the catalyst under ALPS-1, ALPS-2, and CE conditions. Reproduced with permission from Ref. [64]. Copyright 2023, American Chemical Society. **b** Comparison of current density and product selectivity between PDE and CE. **c** Potential program and reaction strategy for PDE (*E*_c_ = -0.18 V vs. RHE, *t*_c_ = 590 s; *E*_a_ = 1.22 V vs. RHE, *t*_a_ = 10 s). **d** Current density-time curve and net charge transfer during PDE. Reproduced with permission from Ref. [65]. Copyright 2019, Springer Nature. **e** Schematic of reverse current after AWE shutdown. **f** Relationship between the reverse current on each bipolar plate at the anode side and the shutdown time, depicted by experimental data (solid line) and simulation results (dashed line), under conditions of 30 °C temperature, a current density of 0.6 A cm^−2^, and an operating duration of 60 min. Reproduced with permission from Ref. [68]. Copyright 2022, Elsevier. **g** Current density curve relative to startup/shutdown (SU/SD) cycles (*E*_on_ = 4.5 V, *t*_on_ = 10 min, *E*_off_ = 0 V, *t*_off_ = 20 min). Reproduced with permission from Ref. [71]. Copyright 2024, Wiley–VCH

It is evident that PDE can effectively inhibit surface reconstruction and ensure the FE and electrolysis stability of the product. Specifically, for Cu-based catalysts, although they exhibit high activity and selectivity in CO_2_RR, their structural stability is relatively poor. For Cu-based catalysts, their high selectivity and stability depend on their composition and morphology. Therefore, PDE has become a unique strategy for regulating CO_2_RR performance, particularly for Cu-based catalysts. For example, Zhang et al. used an asymmetric low-frequency pulsed method (ALPS) to significantly improve the stability and selectivity of the Cu_3_(DMPz)_3_ catalyst in CO_2_RR [64]. As shown in Fig. 7a, under conventional CE conditions, Cu_3_(DMPz)_3_ exhibited poor CO_2_RR performance, with a FE of 34.5% for C_2_H_4_ and 5.9% for CH_4_, and low stability within less than 1 h. Subsequently, they optimized two different ALPS methods for CH_4_ and C_2_H_4_, respectively. The result was high-selectivity catalytic products, with CH_4_ FE = 80.3% (above 76.6% after 24 h) and C_2_H_4_ FE = 70.7% (above 66.8% after 24 h). The improved CO_2_RR performance was attributed to the ability of the ALPS method to significantly control the size, crystallography, and oxidation state of Cu-based nanoclusters. For Pd-based catalysts, due to their strong binding with CO, during CO_2_RR producing HCOO^−^ or HCOOH, the reaction with the simultaneously generated CO blocks the active sites of the catalyst, thus promoting catalyst degradation. Notably, PDE can remove surface CO and form a very thin oxide-derived layer on the catalyst surface, which helps slow down the catalyst degradation and simultaneously suppress side reactions [16]. Therefore, using the PDE strategy during CO_2_RR production of HCOO^−^ or HCOOH helps achieve higher FE and longer stability, resulting in more selective CO_2_ conversion products. For example, Lee et al. employed a two-step electrolysis method by alternating reduction and oxidation potentials, achieving 100% current density stability and 97.8% HCOO^−^ production selectivity for at least 45 h [65] (Fig. 7b). This is likely because the additional oxidation step can specifically control the adsorption of the CO intermediate on the electrode, allowing the Pd-based catalyst to maintain stability for more than 45 h (Fig. 7c, d). Additionally, the study found that PDE helps avoid structural changes that might be induced by the catalyst, further maintaining high stability and selectivity of the reaction.

In addition, the operation of electrolyzers using intermittent renewable energy for hydrogen production may involve frequent daily shutdowns. During shutdown and discharge of the electrolyzer, the electrodes and electrode coatings (catalysts) are more susceptible to corrosion. Electrode corrosion under shutdown conditions, often referred to as reverse current corrosion, occurs due to the discharge of the EDL, leading to polarity reversal of the electrodes when the electrolyzer current reverses. This effect has an unavoidable impact on industrial renewable energy hydrogen production via electrolysis. Specifically, when the catalyst compositions of the anode and cathode differ, and particularly when the cathode catalyst does not exhibit strong oxidation resistance, it is susceptible to oxidative dissolution and deactivation caused by the generated reverse current. Importantly, the specific effects of reverse current on catalysts remain unclear, and effective mitigation measures need further investigation.

When studying the effects of electrolyzer shutdown and reverse current phenomena on electrode performance, discharge characteristics are a key focus. Janjua et al. investigated the effect of open-circuit conditions on electrode stability and found that bipolar electrolyzers discharge faster and more deeply than monopolar electrolyzers [66]. Moreover, the voltage of individual electrolyzer can be reduced more quickly by short-circuiting a single cell with a resistor. Further research focused on how the choice of electrocatalysts affects reverse current phenomena and the matching of materials. Oda et al. studied the discharge capacities of various transition metal-based electrocatalysts to elucidate the determining factors of electrocatalysts for reverse current resistance and durability. Experimental results showed that OER catalysts must be properly matched with HER catalysts on the opposite side of the bipolar plate to avoid the decomposition of one of the catalysts [67]. Certain materials and material combinations that are susceptible to reverse current effects include cobalt molybdate cathodes, nickel molybdate cathodes, unmodified nickel cathodes, nickel–cobalt mixed oxides as anodes, lanthanum strontium cobalt perovskites as anodes, and nickel–cobalt spinel materials. Additionally, the effects of different operating conditions on reverse current phenomena have been thoroughly explored. Haleem et al. studied the reverse current phenomenon in a 4-cell alkaline water electrolyzer (AWE) under shutdown conditions by varying the CE current load [68] (Fig. 7e). As shown in Fig. 7f, the reverse current consistently peaked at the central bipolar plate, and the discharge potential region of the hydrogen electrode was longer than that of the oxygen electrode. Furthermore, it was found that high operating temperatures (80 °C) increased the reverse current, thereby accelerating unfavorable redox reactions on the electrocatalyst during shutdown. Interestingly, during shutdown, the CE operating current density had no significant effect on the reverse current phenomenon. Therefore, rapid cooling of the electrolyte after electrolyzer shutdown or stopping the circulation of the electrolyte can enhance the long-term durability of electrocatalysts.

In the practical application of large-scale renewable energy hydrogen production, electrolyzer systems use external manifold systems to divert current during shutdown and stabilize electrode lifespan. External manifold systems consist mainly of large pipelines installed at the electrolyte inlet and outlet, with grooves on the sidewalls to prevent the formation of continuous liquid films. This design ensures that the returning electrolyte and gases flow in from the top and move toward the lower or side outlets along the manifold. During electrolyzer shutdown, this system provides current protection, preventing significant corrosion of nickel on the cathode side. However, compared to built-in manifolds formed in the gasket region, external manifold systems are more costly and structurally complex [69]. Therefore, in high-pressure AWE/PEMWE systems, external manifold designs may be excluded due to cost limitations. To illustrate the dynamic changes in reverse current under variable load conditions and avoid overdesigning electrolyzer systems, Jupudi et al. developed a shunt/reverse current prediction model for bipolar electrolyzers [70]. This model further demonstrated how the number of single cells in the electrolyzer stack, the size of the inlet and outlet collection manifolds, and the reverse current under dynamic and intermittent loads affect energy efficiency and the risk of electrode corrosion. In addition to system-level considerations, catalyst-level strategies are also theoretically viable. Jung et al. designed a Pb-modified cathodic Ni catalyst (Pb/Ni) from the perspective of catalyst material regulation to enhance the durability of electrocatalysts in AWE systems under load fluctuations and reverse current conditions during renewable energy shutdowns [71]. As shown in Fig. 7g, the Pb/Ni catalyst exhibited excellent reverse current tolerance under repeated shutdown conditions, achieving a reverse current stability factor (RCSF_η_) of 6.35 and a reverse current accommodation factor (RCAF_η_) of 14.11 mA cm^−2^, more than five times that of bare nickel. Over 30 shutdown/startup (SU/SD) cycles, the AWE stack using the Pb/Ni catalyst experienced minimal current loss, while the bare nickel stack showed a 10.5% current loss.

Notably, owing to the excellent “plug-and-play” compatibility of PDE, regulation can be implemented solely on the power supply side, enabling its efficient integration into mainstream high-performance catalyst and membrane electrode material systems and further enhancing reaction activity and operational stability. Wang et al. loaded earth-abundant, non-precious metal Mo onto a Co_9_S_8_ substrate to prepare an advanced OER catalyst, Co_9_S_8_ modified with single-atom Mo (Mo-Co_9_S_8_@C), which exhibited outstanding bifunctional HER and OER activity under acidic, alkaline, and neutral conditions [72]. By precisely tuning the active sites and constructing hydrophilic structure, this catalyst achieved highly efficient overall water splitting across a wide pH range. In addition, for membrane electrode materials, they developed a Zr-regulated Co–Co dual-atom site catalyst (Co–Co DASs/ZCC), which demonstrated OER activity and acid stability in PEMWE systems far exceeding those of traditional noble metals [73]. Such highly active non-precious metal catalysts provide an ideal experimental basis for PDE, which has the potential to further stimulate interfacial reaction kinetics, accelerate bubble detachment, enrich reactants and adsorbed intermediates, lower reaction overpotential, mitigate long-term degradation of electrodes and membranes, and improve electrolysis durability. However, the complex dynamic environment arising from frequent voltage and current changes in PDE, such as electric field perturbations, pH fluctuations, and bubble impacts, requires that key device materials, including catalysts and membrane electrodes, possess higher structural stability and dynamic adaptability. Catalysts, in particular, should exhibit rapid electronic responsiveness and good structural reversibility to withstand active site reconstruction under high-frequency electric fields. Otherwise, irreversible structural transformations, metal dissolution, agglomeration, or passivation may occur, leading to performance degradation. Membrane electrodes should combine excellent mechanical strength with high ion conductivity to endure pH, temperature, and stress fluctuations induced by periodic electric fields, thereby preventing catalyst detachment from interfaces as well as structural damage and deterioration of ion transport performance in ion exchange membranes [74, 75].

In short, the load conditions of the electrolytic cell before the periodic power supply is stopped have little effect on the discharge of the cathode and anode. During HER and OER, the electrode surfaces are almost completely covered by the respective reactions, and further increases in load do not lead to additional charge accumulation. Furthermore, after prolonged periods of cyclic startup/shutdown operation with power input, the cathode may form related hydrides, and the anode may develop new oxide structures. At this point, the discharge behavior may change, and variations in the discharge curve and behavior can provide critical insights into degradation mechanisms and the state of the electrodes.

### Electrode Flooding

The phenomenon of electrode flooding typically refers to the accumulation of excessive liquid water in the flow channels or porous electrodes, which cannot be expelled. This results in most of the pore volume in the GDL being occupied by liquid water, preventing the reactant gases from entering the catalyst layer. Electrode flooding typically occurs in the GDL structure of flow cell. Although hydrophobic materials such as polytetrafluoroethylene (PTFE) are commonly used as the GDL base, the hydrophobic chemical structure gradually degrades over time, leading to water saturation and performance degradation. Degradation of the GDL causes water penetration and flooding of the GDE, hindering the escape of gas from the catalyst surface to the three-phase interface. This insufficient escape isolates the active sites on the electrode surface from the active ions, reducing the rate of reactions and increasing the interfacial resistance between the electrode and the electrolyte. This further results in higher overpotentials, lower energy efficiency, and mass transfer losses, which further affect the HER. Additionally, the protons generated at the anode GDE may also be negatively affected by the flooded GDE during their migration through the PEM to the cathode. This phenomenon is most likely to occur under high voltage and high electrolyte concentration conditions.

**Fig. 8:**
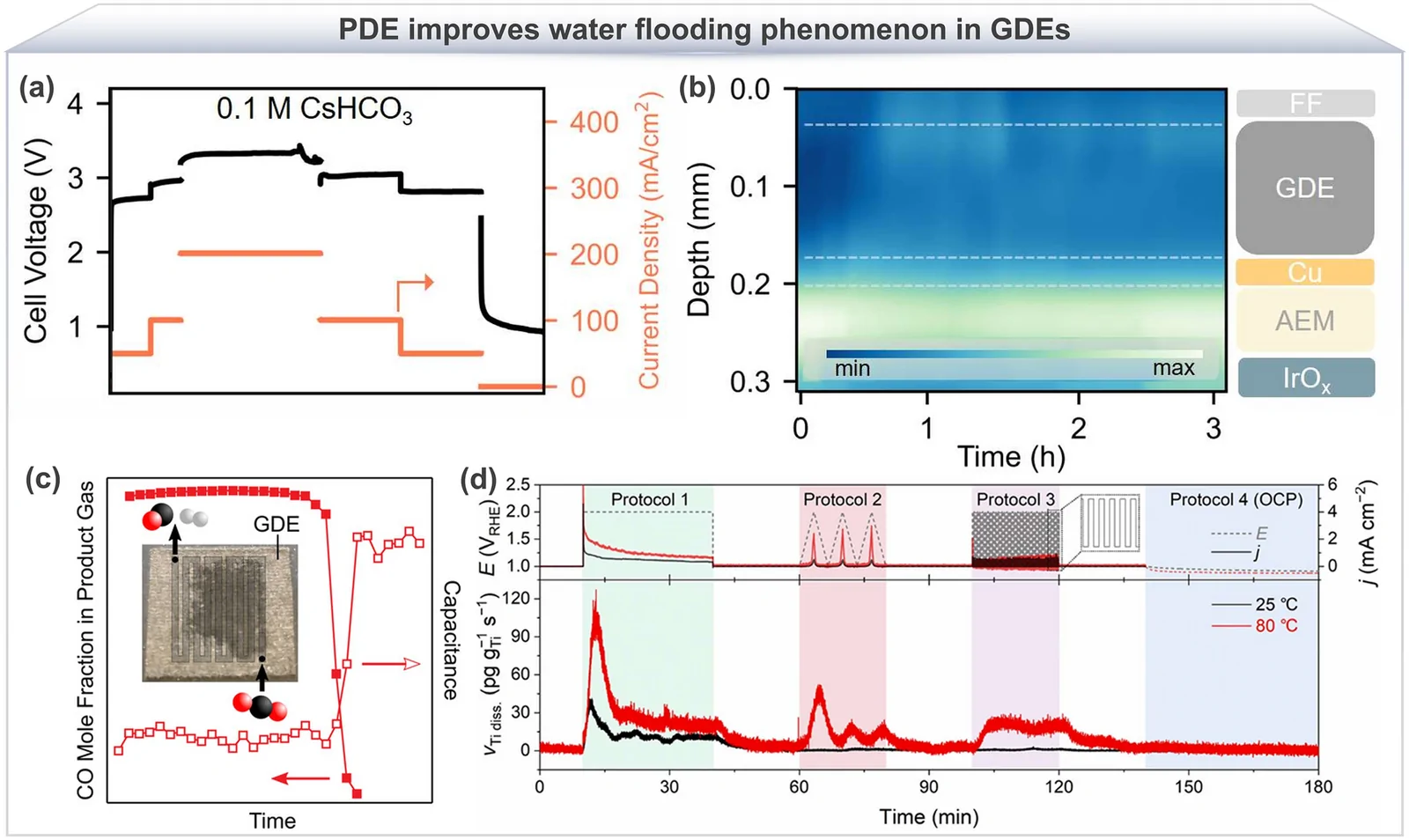
PDE improves the water flooding phenomenon in GDEs. The evolution of the H_2_O signal inside the MEA during CO_2_ electrolysis in 0.1 M CsHCO_3_ electrolyte, **a** the operating current density and cell voltage, and **b** the transition from dark to light color showing the gradual increase in H_2_O content. Reproduced with permission from Ref. [76]. Copyright 2024, Elsevier. **c** The inverse relationship between CO selectivity and EDL capacitance, the latter being a characterization of the electrode flooding area. Reproduced with permission from Ref. [78]. Copyright 2019, Wiley–VCH. **d** Real-time dissolution curve of Ti-PTL at different potentials in a saturated oxygen 0.1 M HClO_4_ solution at 25 °C and 80 °C. Reproduced with permission from Ref. [79]. Copyright 2024, Royal Society of Chemistry

To further explore the potential applications of PDE in GDE design, relevant research has gradually shifted focus toward the mechanisms of water migration and electrode flooding. Joensen et al. investigated the mechanism of GDE flooding in situ by applying step current and recovering to the OCV state (Fig. 8a), revealing the effect of voltage changes on water migration [76]. They made an important observation: during the current application process, the combined effects of the electric field, ion migration, concentration diffusion, and electroosmotic drag lead to the movement of water and the flooding phenomenon in the GDE (Fig. 8b). This study effectively explains the dynamic behavior of water in electrochemical processes, especially how electroosmotic drag and diffusion effects affect the distribution of water at the electrode interface under high current density. Building on existing work, future research could further expand the complexity of experimental conditions to more comprehensively verify the effect of water migration on GDE performance. Furthermore, these findings will provide important theoretical support and practical guidance for optimizing GDE design and PDE operating strategies. Additionally, some researchers have found that periodic PDE can help regulate the dynamic distribution of water during the electrolysis process, preventing excessive liquid water accumulation in the GDL and thus avoiding excessive flooding. Xu et al. used a PDE strategy, experimentally reducing the applied voltage periodically for short times in a flow cell. Their results showed that the pulsed periodic electrolysis method helps avoid saturation and prevents GDL flooding. They conducted a 157 h operation on Cu catalysts for C_2+_ products, maintaining 80% product selectivity and a partial current density of 138 mA cm^−2^, with no flooding recorded [77]. Therefore, PDE not only provides an effective means to address electrode flooding but also opens up new development directions for improving electrolysis efficiency and extending electrode lifespan. Future research should focus on exploring the specific impact of typical PDE parameters on electrolysis performance to further optimize its application effectiveness. The GDE flooding phenomenon may be attributed to the formation of carbonate precipitates blocking the cathode flow field. Leonard et al. characterized the extent of electrode flooding by periodically restoring electrode activity by switching the cell voltage to the OCV, while observing the relationship between carbon monoxide selectivity and the EDL capacitance [78]. As shown in Fig. 8c, after operating at current density of 50 mA cm^−2^ for 1 h and switching the cell voltage to OCV for a long period, they found that the gas at the outlet was not submerged by the electrolyte. Cho et al. combined PEMWE with online inductively coupled plasma mass spectrometry (ICP-MS) to monitor the in situ dissolution rate of Ti in O_2_-saturated 0.1 M HClO_4_ as a function of potential/time [79]. Experiments were conducted at 25 and 80 °C. When a constant potential of 2 V vs. RHE was applied, Ti PTL exhibited a significant titanium dissolution rate of about 100 pg g_Ti_^−1^ s^−1^. Within 500 s of holding the potential, the dissolution rate of titanium rapidly decreased and stabilized at approximately 25 pg g_Ti_^−1^ s^−1^. In contrast, when a 0.1 Hz pulsed potential was applied, the dissolution of Ti was significantly lower than that under CE conditions (Fig. 8d).

In short, the phenomenon of electrode flooding significantly affects GDE and electrolysis performance, especially under high current density and concentrated electrolyte conditions. Flooding causes the pores in the GDL to be occupied by liquid water, hindering the entry of reactive gases into the catalytic layer, increasing interface resistance, and reducing the reaction rate. PDE helps regulate the distribution of water by periodically adjusting the voltage, preventing excessive flooding of the GDE, thereby improving electrode stability and reducing energy loss. Future research should further explore the application of PDE under actual complex conditions, identify optimal pulsed parameter combinations, and combine advanced electrode materials and device designs to enhance the stability and efficiency of electrolysis systems.

### Impurity Deposition

In fields such as pollutant degradation, CO_2_RR, and direct seawater electrolysis, aqueous electrolytes with complex compositions are widely used. However, once the impurity concentration in the solution reaches the ppm level, it can sufficiently contaminate the catalyst active sites and trigger electrochemical deposition on the electrode surface. During the electrolysis process, the deposition of these impurities on the catalyst surface may exacerbate side reactions. In localized electrolysis environments with high salt concentrations, this effect can become more exacerbated. The additional deposition of substances on the catalyst surface is typically referred to as catalyst poisoning, which leads to significant changes in the structure, morphology, and active surface area [80]. This can result in altered catalytic activity and reduced product selectivity.

**Fig. 9:**
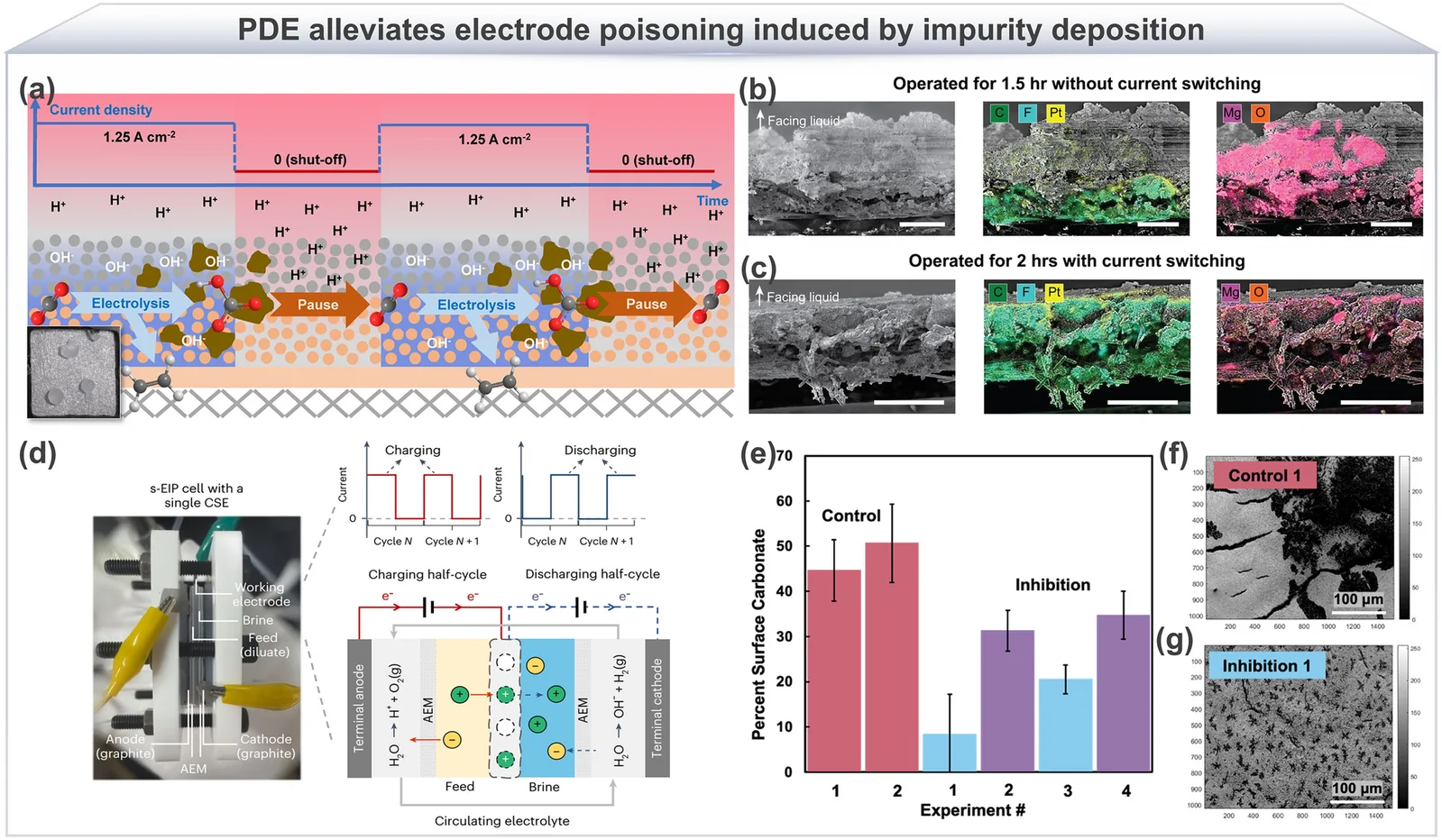
PDE alleviates electrode poisoning induced by impurity deposition. **a** Schematic diagram of the mechanism for alleviating salt formation by PDE. The inset shows the salt crystals deposited on the backside of the Cu GDE after 6 h of constant CO_2_ electrolysis at -1.25 A cm^−2^. The red and blue regions represent acidic and alkaline environments, respectively. Reproduced with permission from Ref. [82]. Copyright 2023, Elsevier. SEM and EDS cross-sectional images of the GDE after 1.5 h of operation in actual seawater, **b** without current switching and **c** with current switching, with a scale of 200 μm [49]. Copyright 2024, National Academy of Sciences. **d** Photograph (left) and schematic diagram (lower right) of the EIP system, along with the current waveform of a single-electrode EIP system during charging and discharging. Reproduced with permission from Ref. [83]. Copyright 2024, Springer Nature. **e** Comparison of the surface carbonate percentages in control and PDE inhibition experiments. Red represents the comparison group, while blue and purple show the effects of different inhibition methods. **f** SEM image of the cathode in the comparison experiment. **g** SEM image of the cathode in PDE, showing reduced surface carbonate deposition. Reproduced with permission from Ref. [85]. Copyright 2022, American Chemical Society

PDE can periodically remove impurities from the catalyst surface, reducing impurity poisoning without the need for regular electrode cleaning, disassembling the electrolyzer, or using chemical solutions for activation. Yano et al. applied a PDE strategy of anode and cathode polarization to a copper electrode for CO_2_RR. The results showed that the anode polarization intervals inhibited the deposition of poisoning species on the electrode, and the FE of CH_4_ and C_2_H_6_ only slightly decreased after 1 h [81]. This could be due to the oxidation of the copper electrode to copper oxide during the anode potential step, with the poisoning species also being further oxidized, thus effectively regenerating the catalyst active sites. Building on the advantages of PDE in CO_2_RR, Xu et al. further studied the effects of PDE on salt formation and interface acidity. During CO_2_RR in an acidic medium flow cell to produce C_2_H_4_, they found that in the CE process, H^+^ was consumed at a fast rate, causing the interface to become alkaline, which is the main reason for salt formation [82]. During the PDE intervals, protons in the acidic electrolyte diffuse back to the interface, while due to the concentration gradient generated during CE, carbonate ions tend to move away from the electrode surface (Fig. 9a). The restoration of H^+^ concentration at the interface reacts with carbonate ions, thus slowing down salt formation and locally regenerating CO_2_, leading to efficient C_2_H_4_ production, with partial current density (*j*$\mathrm{C}_{2}\mathrm{H}_{4}$_C_2H4) exceeding 470 mA cm^−2^ and FE$\mathrm{C}_{2}\mathrm{H}_{4}$ reaching 40%. Additionally, the effect of salt clogging in electrochemical reactions on system performance has also attracted researchers’ attention. Guan et al. designed an electrochemical coupling of HER and hydrogen oxidation reaction (HOR) to improve CO_2_ capture in seawater alkalinity systems [49]. In conventional CE, metal ions in seawater behave inertly under electrochemical reaction conditions, and as alkalinity increases, salt precipitates (Fig. 9b). To address this, by switching the current direction every 30 min between two chambers, the GDE lifespan was effectively extended, preventing salt clogging (Fig. 9c). Inspired by the ion shuttle phenomenon caused by circuit switching, Xu et al. further explored the application of an electrochemical ion pumping (EIP) system [83]. This system applies pulsed currents (10 A m^−2^) in the circuit for ion transport resistance and capacitive adsorption/desorption related to the desalination process (Fig. 9d). They found that the system showed significant performance improvement in desalination of freshwater, achieving nearly complete desalination of 100 mM NaCl with a current efficiency of about 80%. Furthermore, regarding the issue of carbonate deposition in electrolysis processes, Cofell et al. conducted related studies. In a flow alkaline CO_2_ electrolyzer, they found that GDEs experienced material performance degradation due to surface carbonate deposition. These deposits, after operating at currents of −50 to −200 mA cm^−2^ for 6 h, blocked the catalyst layer surface or microporous layer and carbon fiber substrate of the electrode, thus reducing CO_2_RR performance [84]. Therefore, Cofell et al. in subsequent research explored a specific potential cycle (regeneration potential −1.60 V for 30 s; operating potential −2.75 V for 60 s) to inhibit carbonate formation [85]. By interrupting the high concentration of OH^−^ in the catalyst layer, carbonate generation was inhibited (Fig. 9e). It was found that during 6 h of cycling operation, carbonate deposition was significantly reduced, and compared to the CE experiment, the carbonate deposition on the catalyst layer surface was reduced by 51% (Fig. 9g compared to Fig. 9f, where the carbonate dark spots were reduced).

In practical applications, further exploration of the optimal operating potential and regeneration potential range is needed to adapt to different electrolyte types and reaction requirements, thus extending its application scope. However, while short-term experiments (6 h) indicate that this strategy effectively reduces carbonate deposition, for long-term operation and commercialization, how to maintain the sustained effectiveness of this inhibition and whether there will be other side effects (such as corrosion or degradation of electrode materials) remains a concern. Therefore, the PDE cycling strategy can be combined with other optimization measures (such as optimizing the structure design of GDEs, improving catalyst materials, etc.) to enhance overall performance. By combining advanced materials and novel electrode designs, carbonate deposition can be further reduced, and the efficiency of CO_2_RR can be improved.

## Mechanism and Research Progress of PDE Enhanced WE

The regulatory mechanisms of PDE in systems such as CO_2_RR are universal and also play a key role in WE for hydrogen production. Specifically, the understanding of how PDE regulates intermediate adsorption/desorption, local pH, and electrode stability in microenvironments and degradation processes provides important theoretical foundations and insights for further analyzing its role in mitigating bubble coverage, concentration polarization, and EDL capacitance loss during WE. Currently, many electrocatalytic hydrogen production reactions generally face issues such as slow kinetics, high-energy consumption, and low hydrogen production rates. The root cause of these problems lies in the spatiotemporal mismatch between the electron transfer rate at the electrode/solution interface, the electrochemical reaction rate, and the gas diffusion rate. To address these challenges, PDE hydrogen production has attracted the attention of many researchers due to its controllability advantages. Notably, PDE can be directly applied to existing conventional WE systems (such as alkaline electrolysis and PEM) without requiring changes to the electrolyzer structure or electrode materials, and it can significantly improve reaction efficiency. PDE is essentially a performance optimization strategy based on existing systems, aiming to reduce energy consumption and enhance hydrogen production efficiency without altering the structure of electrolytic system.

### Development History

**Fig. 10:**
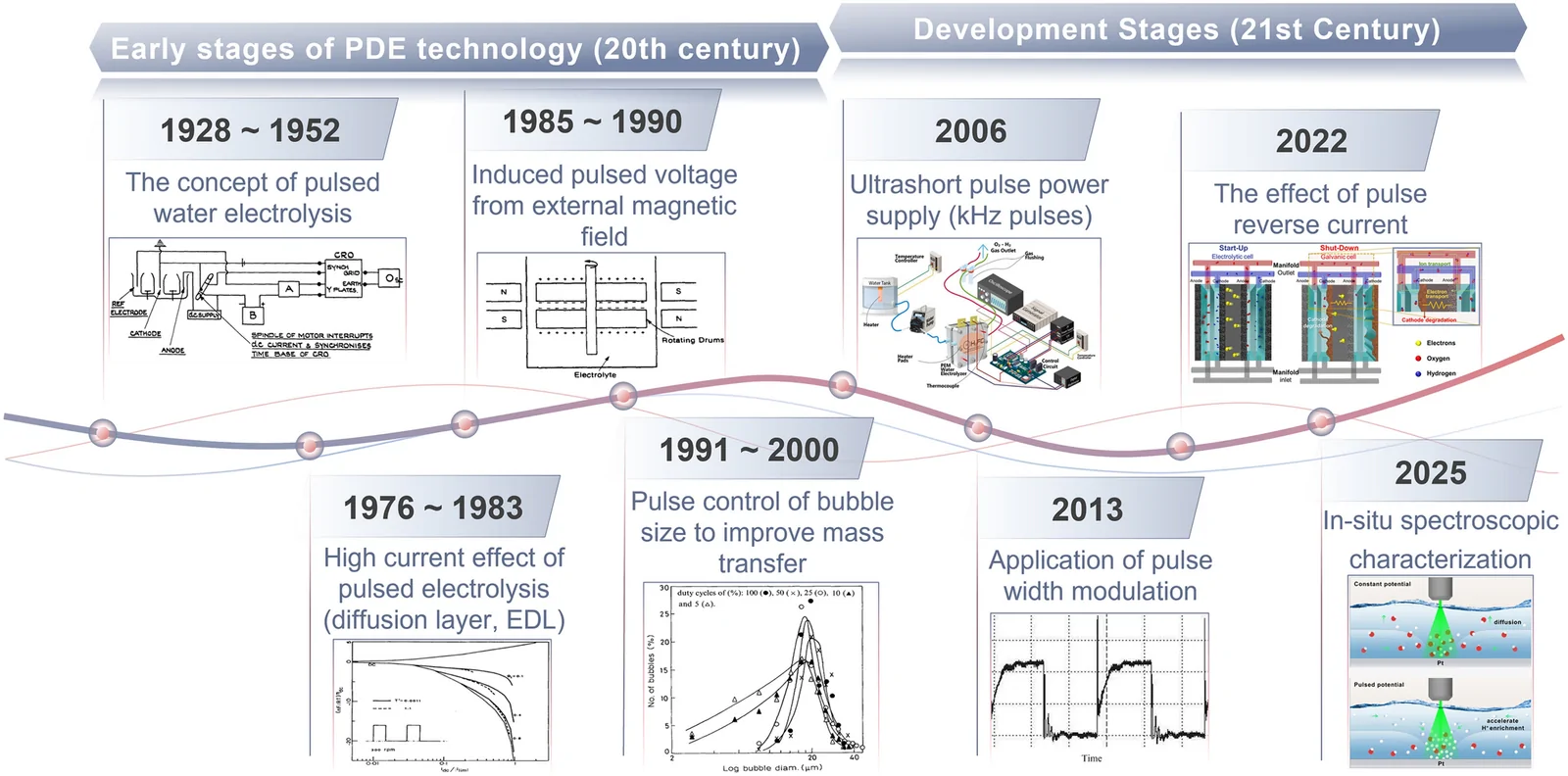
Primary development timeline of pulsed WE technology. The inset: the concept of pulsed water electrolysis. Reproduced with permission from Ref. [88]. Copyright 1952, AIP Publishing. High current effect of pulsed electrolysis. Reproduced with permission from Ref. [97]. Copyright 1983, IOP Publishing. Induced pulsed voltage from external magnetic field. Reproduced with permission from Ref. [98]. Copyright 1985, Elsevier. Pulse control of bubble size to improve mass transfer. Reproduced with permission from Ref. [93]. Copyright 1991, Springer Nature. Ultrashort pulse power supply. Reproduced with permission from Ref. [7]. Copyright 2024, Elsevier. Application of pulsed width modulation. Reproduced with permission from Ref. [99]. Copyright 2013, Elsevier. The effect of pulse reverse current. Reproduced with permission from Ref. [100]. Copyright 2022, American Chemical Society. In situ spectroscopic characterization. Reproduced with permission from Ref. [55]. Copyright 2025, Elsevier

The concept of PDE was first proposed by Bowden and Rideal in 1928, primarily for short-term electrochemical measurements. Subsequently, Butler and Armstrong expanded its application range and experimentally validated its significant advantages in kinetic measurements in 1933 [86]. However, due to limitations in laboratory equipment and variations in operational parameters, early PDE faced issues with reproducibility. It was not until the development of dropping mercury electrodes (DMEs) that PDE achieved a breakthrough. DMEs continuously refreshed the mercury drop to regenerate the electrode surface, significantly reducing the effect of surface heterogeneity on experimental results, thereby greatly improving reproducibility and reliability [87]. Against the background of improved pulsed electrochemical measurement techniques, Bockris et al. first proposed the concept of pulsed WE in 1952 to study the kinetics of WE reactions [88]. Through transient methods, they observed that during PDE, the current exhibited transient spikes and continued to flow momentarily after the potential was turned off, indicating that pulsed electric fields might significantly influence the hydrogen generation process. With further research, Tseung et al. conducted a series of experiments in the 1970s, revealing the effects of PDE on electrode surface conditions. In their studies in 1976 and 1979, they designed pulsed voltage experiments, showing that PDE significantly increased the current density compared to CE [89, 90]. For instance, on PTFE-bonded Pt electrodes, applying a 50 mV overpotential resulted in a current density of up to 0.5 A cm^−2^ within 0.1 ms. During this period, the industrial application of PDE began to attract attention. Puippe et al. introduced it into the electroplating industry in 1980. They discovered that the intermittent effects of pulsed currents might be limited by the charging and discharging rates of the EDL at the electrode/solution interface [91]. Moreover, although they proposed a mechanism of current decay caused by capacitive damping effects during pulsed currents, their explanations of diffusion processes and non-Faradaic processes were incomplete. To further optimize the effects of pulsed WE, Ghorogchian and Bockris developed a monopolar device for WE under pulsed potential in 1985 [92]. By using a magnetically induced rotating disk to generate triangular pulsed voltages, their experiments showed that when the pulsed width was 600 μs and the average voltage was 2.6 V, the pulsed current intensity could reach twice that of DC. In 1990s, the potential of PDE in electrochemical applications was further explored. In 1991, Khosla et al. investigated the effect of pulsed current on controlling bubble size during HER [93]. Using a 1 cm^2^ Pt electrode and a 1 M Na_2_SO_4_ solution (pH = 10), they adjusted the pulsed duty cycle from 5% to 100%. The results showed that lower duty cycles significantly reduced bubble size. Meanwhile, in 1993, Shaaban et al. introduced pulsed currents into 3-D electrode system composed of dispersed spherical ultramicroelectrodes [94]. By varying the pulsed frequency (10 ~ 40 kHz) and duty cycle (10%-80%), they found that the current density of the 3-D electrode was 100 to 1000 times higher than that of a 2-D electrode under the same geometric area and cell voltage. As research progressed, Hitz and Lasi proposed a method for studying HER kinetics based on pulsed currents in 2000 [95]. Using porous Ni electrodes and disk electrodes, they conducted experiments and discovered through mathematical modeling that pulsed currents could help estimate kinetic parameters and EDL capacitance. Entering the twenty-first century, Shimizu et al., building on previous studies, proposed a new method for hydrogen production through WE using ultra-short pulsed power supplies, offering new directions for the application of PDE [96]. These studies demonstrate that PDE has significant advantages in improving electrolysis efficiency, optimizing bubble behavior, and enhancing electrode performance. The primary development timeline of pulsed WE technology is shown in Fig. 10, highlighting its broad applications and development potential in various fields.

### Enhancement Mechanism

In theoretical WE, the voltage required to split water into hydrogen and oxygen is 1.23 V. However, due to the formation of the EDL, this voltage typically increases to 1.45 V [101]. Notably, a key issue in WE is whether PDE can enhance process efficiency compared to CE operation. PDE effectively reduces the formation of the EDL, thereby lowering the thermoneutral voltage, reducing the required energy input, and improving overall energy efficiency. The advantages of pulsed WE have garnered widespread attention in academia. For instance, Demir et al. used transistors to generate voltage pulses and demonstrated that PDE helps remove bubbles from the electrode surface, enhancing electrolysis efficiency by increasing the effective electrode surface area [102]. PDE not only provides higher energy efficiency and lower losses but also effectively decouples the two half-reactions involved in hydrogen generation. Moreover, PDE offers significant safety advantages, particularly at low current densities, by effectively preventing gas mixing [103].

In terms of mass transfer advantages, PDE exhibits significant benefits. The mass transfer process is often the primary limiting factor in electrochemical reactions. When current passes through the electrode, the electrochemical reaction occurs at the electrode/solution interface. Over time, a diffusion layer forms on the electrode surface, gradually hindering the rate at which substances from the solution enter the electrode/solution interface layer, thereby limiting the reaction rate [51]. Under conventional CE conditions, the diffusion layer continuously thickens and stabilizes at a certain thickness, eventually becoming the rate-limiting bottleneck. In the PDE strategy, the electrolysis process alternates between “ON” and “OFF” states, effectively controlling the duration of current flow. During the current-on phase, the diffusion layer cannot thicken rapidly, and the current-off phase allows the diffusion layer to dissipate. This enables reactants in the solution to enter the electrode/solution interface more easily, while products at the interface can diffuse back into the bulk solution, restoring uniformity in concentration [55, 104]. With the resumption of the current-on phase, the electrochemical reaction continues. This periodic process accelerates ion diffusion, reduces the anodic overpotential, and mitigates concentration polarization caused by deviations of the electrode potential from the equilibrium potential, ultimately achieving energy-saving effects [105].

Despite its numerous advantages, pulsed WE also has certain limitations in practical applications. During experiments, if the cell structure is suboptimal, polarity reversal may occur during the power-off phase, leading to reaction reversal and cathode corrosion. In electrolysis, the cathode serves as the site for the HER, responsible for producing the desired hydrogen gas. If the catalytic active sites are lost, the reaction rate and efficiency of electrolysis will be significantly reduced, thereby weakening the overall effectiveness of the PDE. Additionally, energy losses caused by internal resistance can increase the required current density, which in turn raises the voltage demand. This indicates that higher overpotentials must be overcome to sustain the conversion of current density, further adding to the challenges of applying PDE in practical.

#### Diffusion Layer

When transient pulsed voltage is applied, two phenomena may occur: first, rapid electron transfer between the electrode and the water molecules adsorbed on its surface, resulting in the generation of hydrogen and oxygen. Second, spontaneous charge accumulation on the electrode surface leads to the formation of EDL and diffusion layer. This phenomenon can typically be described by the Gouy-Chapman-Stern (GCS) model, which incorporates the Helmholtz plane to account for the finite size of ions. These two phenomena may occur independently or simultaneously, depending on the experimental conditions [106]. Their behavior can vary under CE or PDE conditions. As the duration of the pulse increases, water near the electrode is gradually consumed, leading to the formation of a diffusion layer and transitioning into a diffusion-controlled kinetic phase, which contributes to the decrease in current over time. Meanwhile, once the EDL is fully established, the transient current caused by the charging effect on the electrode surface significantly decreases [107]. The former is typically a gradual process, while the latter occurs rapidly. These two phenomena jointly affect the current response to transient potential, creating a notable difference compared to steady-state measurements. This interplay is a typical characteristic of pulsed WE.

**Fig. 11:**
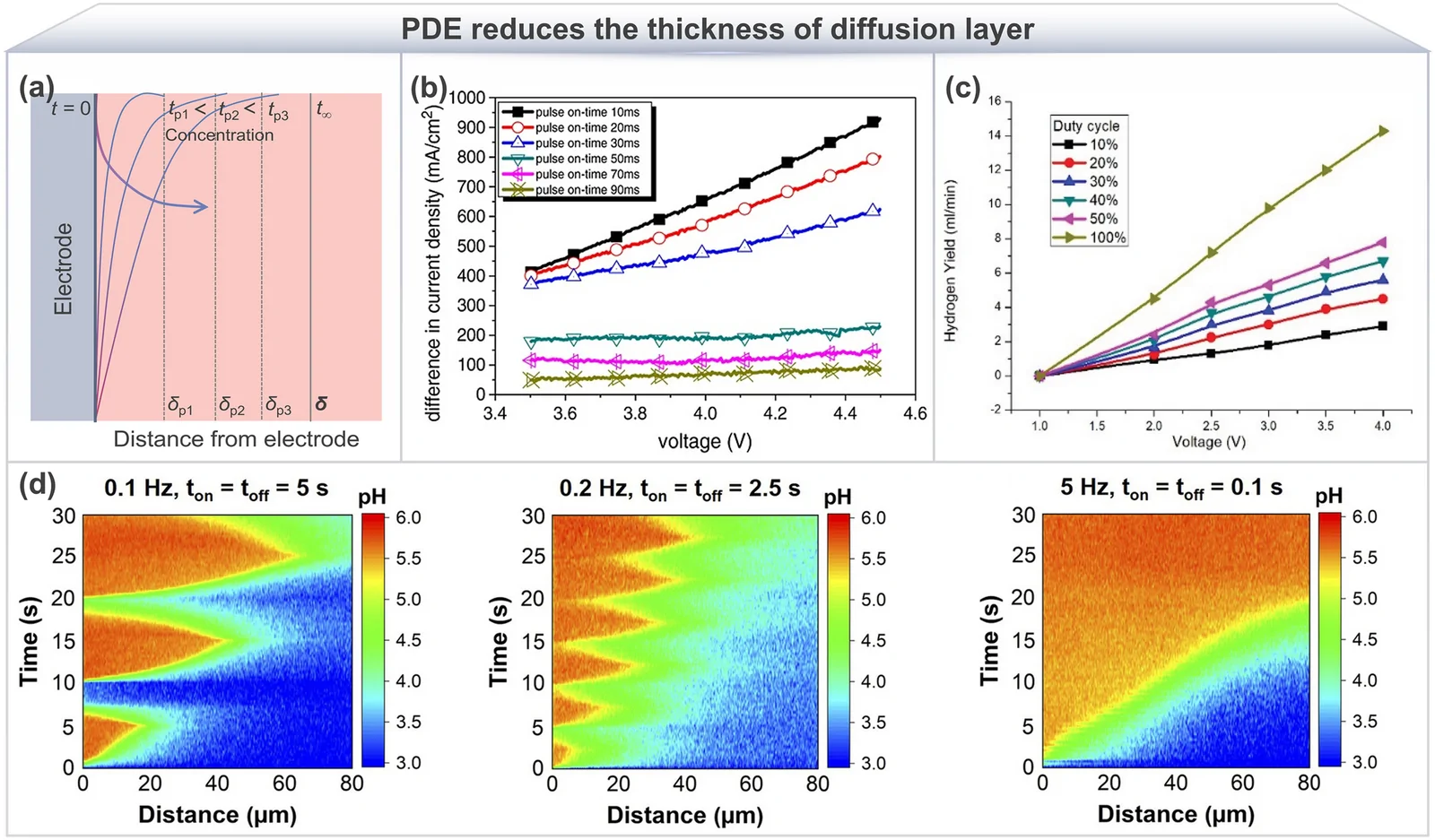
PDE reduces the thickness of the diffusion layer. **a** Schematic of the diffusion layer, where *δ*_p_ is the pulsed dynamic diffusion layer, and *δ* is the nernst diffusion layer. Adapted with permission from Ref. [108]. Copyright 2018, Elsevier. **b** The current density differences for individual voltage pulses with varying on-time durations. Reproduced with permission from Ref. [109]. Copyright 2013, Wiley–VCH. **c** Hydrogen production rate in a dual electrolysis cell at different duty cycles [110]. Copyright 2019. **d** Variation in pH distribution at the interface under 2 V pulsed voltage with different frequencies (initial [HCrO_4_^−^] = 0.2 mM, pH = 3). Reproduced with permission from Ref. [51]. Copyright 2022, Wiley–VCH

The effect of frequency on pulsed voltage and CE powered can be explained using the concentration diffusion layer model. In this model, the concentration distribution is a function of time and individual pulses, as shown in Fig. 11a. The concentration distribution consists of a stationary layer (*δ*_s_) and a pulsed dynamic diffusion layer (*δ*_p_) that extends from the electrode surface into the bulk solution. The thickness of *δ*_p_ is proportional to the pulsed duration, i.e., shorter pulsed durations result in thinner diffusion layers. Under pulsed current, before the concentration layer reaches a steady state, the current is interrupted, allowing reactive ions to diffuse back to the electrode surface and restore the surface concentration to its initial level before the next pulse. As the pulsed frequency changes, the concentration of reactants in the electrode region also varies accordingly [108]. This indicates that during the pulsed duration (*t*_on_), PDE facilitates the accelerated diffusion and consumption of reactive ions, leading to a higher current density compared to CE. Meanwhile, during the pulsed interval (*t*_off_), reactive ions accumulate at the electrode surface, effectively mitigating the polarization effect at the electrode interface.

In CE conditions, the current is applied continuously, which helps the *δ*_p_ to reach the steady-state nernst diffusion layer (*δ*). However, this also leads to ion diffusion in the electrolyte becoming the main limiting factor at higher current densities, thereby increasing the voltage requirement. In PDE, the current is applied in interrupted square wave form, and this interruption process limits the *δ*_p_ from reaching steady state [96]. Due to the pulsed current interruptions, the diffusion layer is thinner, allowing reactive ions to quickly diffuse back to the electrode surface and restore the surface concentration to its initial value before the next current cycle. As PDE is gradually applied, Lin et al. revealed the significant effects of pulsed time and frequency on electrolysis efficiency and current density. They found that as pulsed time shortened, the current density increased significantly, while an increase in pulsed frequency helped improve electrolysis efficiency [109]. Additionally, under low-duty cycle conditions, not only was the rate of increase in current density higher, but power consumption was also significantly reduced (Fig. 11b). Compared to CE, the diffusion layer thickness under pulsed voltage was noticeably thinner. This thinning effect allowed bubbles to detach from the electrode surface more quickly, optimizing the gas release process. Subsequently, Hourng et al. further investigated the application of pulsed voltage in dual-cell (acid–base) electrolysis and conducted an in-depth study of its kinetics and efficiency. They found that as the duty cycle decreased, the current density significantly increased under the same applied voltage, leading to an improvement in energy efficiency [110]. However, although lower duty cycles resulted in thinner diffusion layers, the hydrogen production rate was relatively lower (Fig. 11c), indicating a need to balance efficiency and yield. To further reveal the microscopic effects of pulsed voltage on the electrode/solution interface dynamics, Xin et al. adopted an innovative method combining microfabricated electrolysis cells and laser scanning confocal microscopy. Through high spatial resolution observations, they found that under CE conditions, the electrode/solution interface formed a concentration gradient due to reactant consumption and product accumulation. In contrast, under PDE, the diffusion layer was continuously refreshed by promoting ion diffusion at the interface [51]. The study showed that optimizing pulsed frequency was crucial for reaction efficiency. For example, at 0.1 Hz, the diffusion layer thickness was significantly smaller than at 0.2 and 5 Hz (Fig. 11d). This indicates that in practical applications, pulsed frequency and duty cycle should be adjusted according to the electrochemical reaction process to achieve optimal performance.

#### EDL

**Fig. 12:**
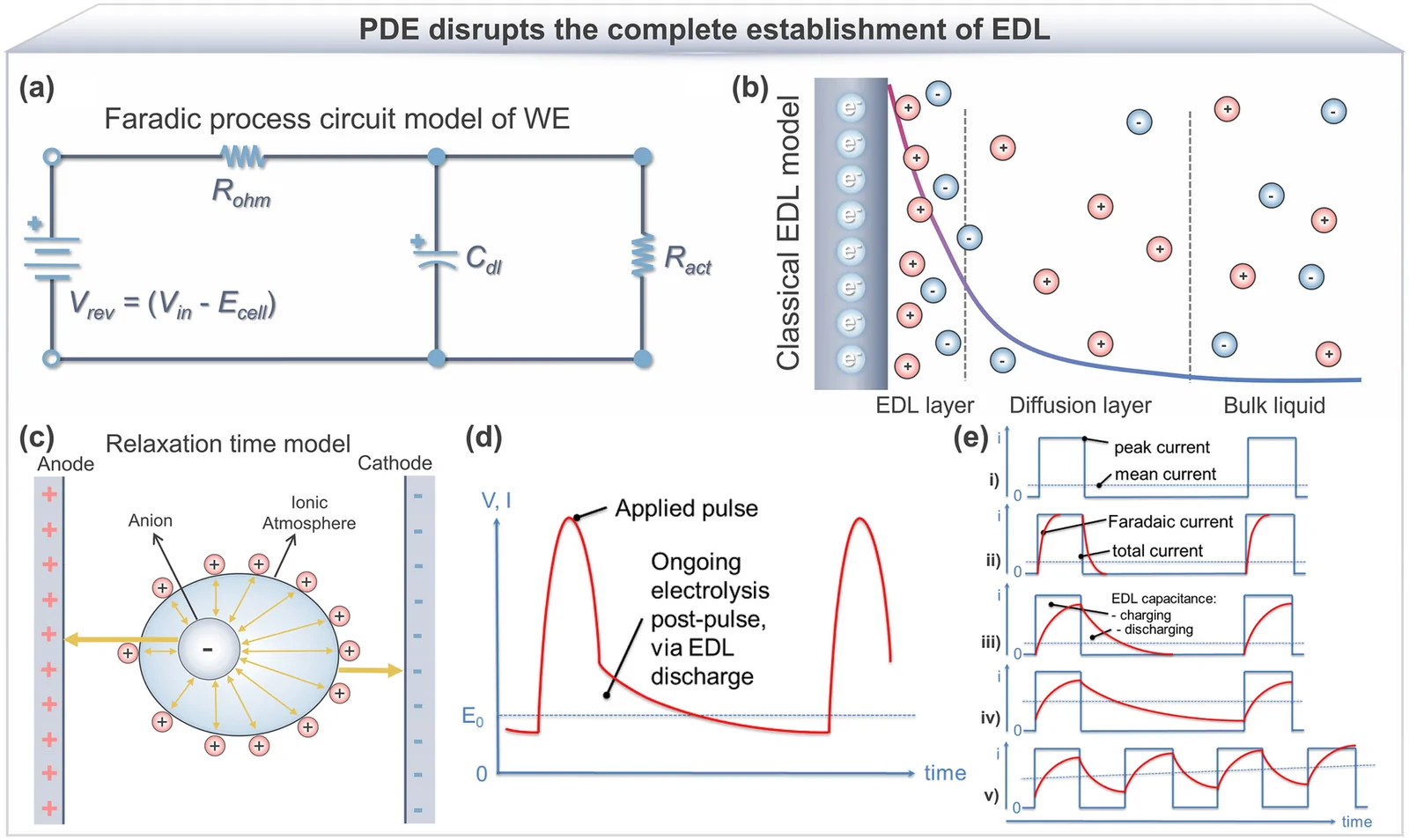
PDE disrupts the complete establishment of EDL. **a** Faradic process circuit model of WE. **b** Classical EDL model. **c** Relaxation time model. **d** Persistent current after PDE completion. **e** Pulsed charging current representing the capacitive load of the EDL. Reproduced with permission from Ref. [106]. Copyright 2016, Elsevier

The generation of charge typically originates from the ionization of substances on the electrocatalyst surface, the adsorption of ions from the solution onto the material surface, and the dissolution of ions from within the material into the solution. This results in the attraction and repulsion between the generated charges, leading to the formation of the EDL on the surface of the electrode material. The EDL consists of counterions adsorbed on the surface of electrode and the interactions between them [111, 112]. The polarization between the surface charge of the electrode and the counterions causes the electrode to exhibit a positive and negative electrode structure similar to a capacitor, which is mainly exhibited in the non-Faradaic process. Meanwhile, the Faradaic process manifests as the load (*R*_act_) of the electrolysis cell being in parallel with the EDL capacitor, and the mass transfer resistance of reactant ions is represented by *R*_ohm_ [113, 114], as shown in Fig. 12a. When a voltage is applied to the electrolyzer, the EDL theoretically forms immediately, resulting in a capacitive effect, which causes the electrolyzer to act as a capacitor to some extent for the non-Faradaic process (Fig. 12b). In this case, DC must provide additional overvoltage to compensate for the energy loss in the “capacitor”, thereby raising the required voltage to the so-called thermoneutral voltage, which indicates that the system does not produce waste heat [115, 116]. One of the goals of PDE is to overcome this issue. Theoretically, when the pulse in pulsed width modulation (PWM) is turned on, the electrolyzer stores capacitance, and when the duty cycle ends, the capacitance is released, allowing current to flow continuously and effectively reducing the formation of EDL.

The improvement in PDE efficiency is attributed to the fact that the EDL and diffusion layer do not have sufficient time to completely form. Some studies suggest that after voltage is applied, ions are immediately attracted to one side of the electrode, while ions surrounding these ions require some time to realign. This process is referred to as the relaxation time [117], as shown in Fig. 12c. As ions move, the surrounding ions have not yet followed, leading to the formation of an asymmetric electric field, which slows down the ion movement and consumes part of the energy provided by the electric field. It is speculated that the relaxation time is in the order of hundreds of nanoseconds. Since the pulsed width in high-frequency pulses is typically shorter than the relaxation time, the surrounding ions of the ions fail to rearrange in time, and therefore, the electric field energy is not excessively consumed. This is one reason for the increased efficiency of high-frequency PDE [18, 118]. Thus, to further improve efficiency, higher pulsed frequencies and shorter pulsed cycles can be explored in future research. Applying high-frequency PDE on a nanosecond time scale significantly reduces the input power compared to conventional CE, achieving a 96.8% energy saving. This phenomenon may be due to the current response time induced by high-frequency PDE being shorter than the relaxation time [119]. The current response curve after applying the pulsed voltage is shown in Fig. 12d, which displays the EDL capacitance charging effect [106]. During the pulsed off period, the stored charge self-discharges through the resistance, causing the electrolysis reaction to continue [120]. To evaluate whether the pulsed voltage induces significant changes, an effective approach is to compare the electrolysis amount caused by this effect with reported energy efficiency. In studies on the optimization of pulsed voltage and duty cycle, Poláčik et al. proposed a method to optimize PDE by adjusting the duty cycle to reduce the EDL impact [121]. They calculated the time required to reduce the EDL to zero using theoretical equations and the Sand equation, which helps improve PDE efficiency. By properly controlling parameters such as the duty cycle, not only can the formation of the EDL be effectively regulated, but the EDL effect can also be partially eliminated, thus improving electrolysis efficiency. In another study, Ereli et al. conducted PDE enhanced PEMWE experiments at 80 °C with pulsed frequencies ranging from 1 to 20 kHz. They found that at high-frequency pulsed voltage (20 kHz), the PDE exhibited the greatest advantage, reducing energy consumption to 8.82525 J mL^−1^ H_2_, compared to 13.446 J mL^−1^ H_2_ at a conventional CE of 1.5 V, representing a reduction of about 35%. Moreover, PDE can effectively reduce corrosion on the electrode or membrane surface, saving energy and extending the lifespan of the equipment [7]. These improvements are closely related to the positive disruptive effects of PDE on the EDL, diffusion layer, and relaxation time. In studies on pulsed current, Puippe et al. used constant current source to provide a constant current square wave curve to further explore the effect of pulsed current on the EDL [91]. As shown in Fig. 12e, the pulsed current exhibits different damping effects under different electrical conditions, ranging from no significant damping to “overdamped” states. This damping effect is closely related to the electrolysis cell's resistance–capacitance (RC) characteristics and has an important impact on the time constant (*T*) of the current cycle. Further investigation of the relationship between the diffusion layer and pulsed width provides important references for understanding the PDE process. Shimizu et al. argued that the thickness of the EDL or diffusion layer must be greater than the diffusion length during the pulsed application. They estimated the maximum pulsed width, suggesting that the pulsed duration should be 1/10 of the critical duration [96]. Through their experiments, it was found that PDE helps avoid the losses caused by diffusion limitations, while improving the efficiency of the electrolysis reaction. By increasing the system input power, despite the increase in frequency, the efficiency was improved, providing important insights for optimizing the PDE process.

In summary, PDE can effectively optimize electrolysis efficiency and reduce the effect of EDL by adjusting pulsed width, duty cycle, and frequency. By applying PDE, energy loss due to diffusion limitations in the electrolysis process can be avoided, especially under high-frequency PDE conditions, where energy consumption is significantly reduced. Additionally, PDE can mitigate corrosion on the electrode surface, extend the device lifespan, and further enhance its application value. In the future, increasing pulsed frequency and shortening pulsed cycles could further improve electrolysis efficiency in shorter period and provide new technological pathways for energy savings and device longevity.

#### Bubbles

Bubbles play a crucial role in electrochemical energy conversion and system performance during the electrolysis process. Their generation and lifecycle usually begin with the decomposition of the electrolyte at the electrode surface, initially forming nanoscale bubbles that rapidly expand into microscale bubbles [122]. The growth of small bubbles is generally limited by their own molecular radial diffusion, while the enlargement of larger bubbles depends more on the generation rate of gas at the substrate. Once a bubble grows to a certain size, it detaches from the electrode surface, carrying away reaction products and being replaced by fresh electrolyte [123]. Under CE conditions, the diffusion rate of substances in the electrolyte is limited by the diffusion coefficient, especially when bubbles cover the electrode surface, often causing significant mass transfer losses. To address this issue, adjusting the pulsed voltage waveform of the power supply has proven to be an effective method. The application of pulsed voltage can mitigate the mass transfer limitations in the electrolyte, reducing losses caused by diffusion [102]. This mechanism is similar to the dielectric relaxation effect of the EDL dynamics at the electrode/electrolyte interface [124]. By applying pulsed voltage, bubble release can be accelerated, improving the relaxation process on the electrode surface, thereby enhancing the reaction rate at the electrode and optimizing the overall electrolysis efficiency.

The cathodic reaction (2H_(ads)_^+^ + 2e^−^ = H_2(g)_) also faces significant mass transfer limitations during WE. According to the HER mechanism, H^+^ gain electrons on the electrode surface to form adsorbed hydrogen atoms *H (Volmer step). Subsequently, *H forms H_2_ molecules through two possible pathways: one involves the combination of two *H atoms to form H_2_ (Tafel step), and the other involves the reaction of *H with H^+^ ions from the electrolyte to generate H_2_ (Heyrovsky step) [125–127]. However, H_2_ bubbles can only detach and release from the cathode surface when they reach a specific critical size, which is affected by the water recirculation rate and the temperature of the electrolytic cell. It is important to note that the bubble generation and release process may not always be the rate-determining step [128]. The incomplete detachment of bubbles formed on the electrode surface is a primary cause of additional ohmic losses [129]. The presence of bubbles not only disrupts the current distribution in the electrolyzer but also isolates the active sites on the electrode surface from active ions in the electrolyte, thereby reducing electrolysis efficiency [130]. To address this issue, bubbles on the electrode surface can be effectively removed by liquid circulation or by enhancing convection through the application of magnetic fields [131]. Moreover, the ohmic resistance of the electrolyzer is limited by the void fraction of the electrolyte between the electrodes. When the electrolyte is forcibly circulated, the residence time of bubbles in the inter-electrode gap is reduced, thereby effectively lowering ohmic resistance [132]. Therefore, solution circulation not only helps to remove bubbles but also significantly reduces additional ohmic losses and improves electrolytic efficiency.

Under conventional CE conditions, the formation of the ion diffusion layer usually occurs near the electrode surface. This process is limited by ion diffusion, resulting in a larger overpotential. Recent studies have demonstrated that using PDE method with interrupted power effectively disrupts the ion diffusion layer near the electrode surface, thereby preventing the formation of the EDL and its associated capacitive losses [106]. This change helps reduce concentration overpotential, promotes bubble detachment from the electrode surface, and restores the electrochemical active area of the electrode [133]. Additionally, PDE enhances the mass transfer of oxygen and hydrogen bubbles, creating a “pumping effect”. This effect effectively mitigates the corrosive effect of oxygen bubbles on the anode, thereby improving the anode's corrosion resistance. The application of PDE provides relaxation for bubbles on the electrode surface, facilitating the diffusion of dissolved gases [134]. By applying pulsed voltage, PDE not only generates a “pumping effect” within the electrolyzer but also effectively refreshes and relaxes the electrode surface. The results show that PDE significantly reduces the mass transfer losses on the electrode surface, thereby reducing the energy consumption for producing 1 mol of H_2_ in the electrolyzer by 20%–25% [102].

**Fig. 13:**
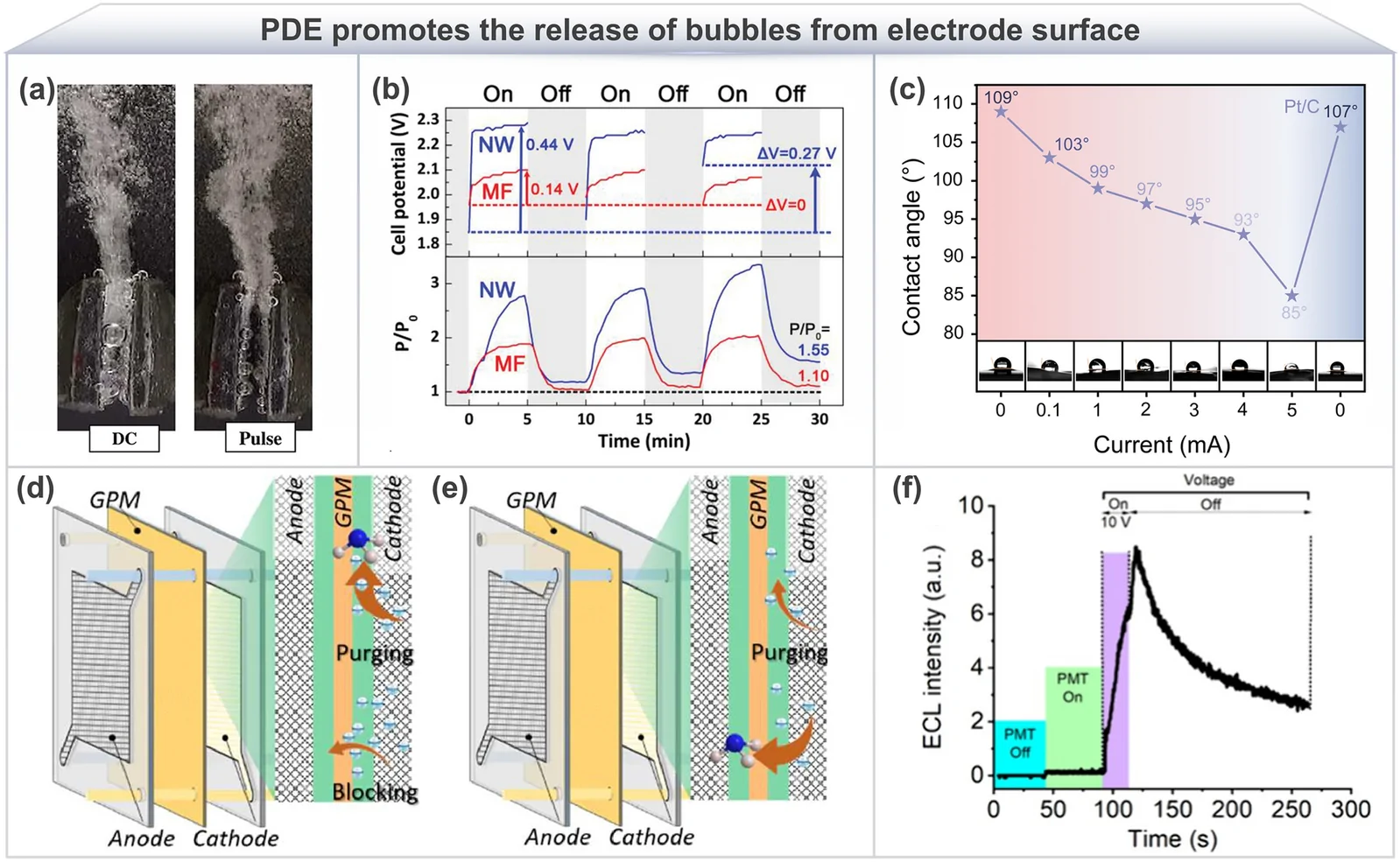
PDE promotes the release of bubbles from electrode surface. **a** Simultaneous generation of oxygen and hydrogen on different nickel electrodes in KOH solution (electrode distance 2 mm, *E*_on_ = 4.0 V, *E*_off_ = 0 V). Reproduced with permission from Ref. [109]. Copyright 2013, Wiley–VCH. **b** Changes in cell voltage and pressure drop (P/P_0_) during PDE on Ni MF and Ni-Cu NW felt electrodes (pulsed current density is 500 mA cm^−2^, 5 min ON + 5 min OFF). Reproduced with permission from Ref. [135]. Copyright 2020, Wiley–VCH. **c** Changes in the contact angle of reaction bubbles released from the cathode Pt/C side under step current. Reproduced with permission from Ref. [55]. Copyright 2025, Elsevier. Schematic of bubble diffusion and mass transfer under **d** CE and **e** PDE strategy. Reproduced with permission from Ref. [46]. Copyright 2024, Elsevier. **f** Light intensity recorded using a photomultiplier tube (PMT), with 10 V voltage applied for 10 s, followed by voltage turned off. Reproduced with permission from Ref. [136]. Copyright 2024, American Chemical Society

The behavior of bubble generation and detachment during hydrogen production has a significant effect on electrolysis efficiency. The application of periodic pulsed potentials in PDE has brought remarkable optimization to this process. Lin et al. suggested that applying periodic pulsed potentials induces instantaneous increases in current, which accelerates bubble detachment from the electrode surface, reduces electrochemical polarization in the diffusion layer, and further improves hydrogen production efficiency [109]. Experimental photos verified this observation (Fig. 13a), showing that the diffusion layer thickness under pulsed potentials is significantly smaller than that under CE, and the bubbles detach and rise from the electrode surface more quickly. In further exploring the performance optimization of PDE, Yang et al. investigated the effects of PDE on bubble removal and electrolysis performance in porous electrodes. They applied a pulsed current of 500 mA cm^−2^ to a porous nickel electrode for 5 min at a time, followed by a 5-min interval without current, in a repeated cycle [135]. As shown in Fig. 13b, low-frequency pulses effectively improved bubble removal efficiency, thereby increasing the active surface area of the electrode. During the *E*_off_ period, time is provided for bubble removal and for recovering the surface area blocked by bubbles. However, if bubbles are confined within the electrode, the pressure drop across the electrode increases, as bubbles reduce the liquid flow pathways. In our previous work, we further expanded on these findings. Using in situ contact angle measurements under an applied electric field, we demonstrated that PDE facilitates the escape of product bubbles from the electrode surface [55] (Fig. 13c). Unlike the macrobubble removal discussed by Yang et al., our work focused on the dynamic behavior of microbubbles. Due to the relaxation effect induced by periodic pulsed intermittent voltage, the growth dynamics of microbubbles on the MEA surface were weakened. Consequently, the average size of bubbles on the electrode surface under PDE was smaller, with a denser distribution. The residence time of these small bubbles on the electrode surface was significantly shortened, further improving electrolysis efficiency. Beyond hydrogen production, the application of PDE strategies has also shown significant potential in bubble-related processes in wastewater treatment. He et al. found that during ammonia recovery from wastewater such as urine, hydrogen bubbles generated under CE conditions could reduce the effective contact area between ammonia and the hydrophobic membrane, thereby hindering ammonia transport. Additionally, the continuous generation of hydrogen bubbles exerted a flushing effect on ammonia, rapidly releasing it from the liquid phase into the gas phase [46] (Fig. 13d). To address this issue, they introduced the PDE strategy, using intermittent power supply to suppress the continuous generation of hydrogen bubbles, thereby reducing ammonia nitrogen loss (Fig. 13e). Under optimized experimental conditions, they achieved nearly 100% ammonia recovery efficiency, demonstrating the broad applicability of PDE. The interaction between bubble behavior and local surface microstructures has also attracted the attention of researchers. Nami-Ana et al. studied the behavior of microbubbles at interfaces resembling hydrophobic/hydrophilic boundaries, revealing the chemical reaction characteristics between microbubbles and microstructured interfaces [136]. These findings align with the advantages of PDE. Upon applying voltage, sticky bubbles generated on the surface could stably adhere for extended periods, while the light intensity gradually decreased over time after the voltage was switched off (Fig. 13f). This phenomenon further validates the relaxation effect during the *E*_off_ period, which promotes bubble escape by reducing the growth dynamics of microbubbles.

In summary, PDE, through the regulation of periodic voltage or current, not only optimizes bubble generation and detachment behavior while reducing diffusion layer thickness but also significantly enhances electrolytic performance across various applications. However, attention should be given to issues such as localized bubble confinement and electrode corrosion resistance.

### Operating Mode

Currently, there are two main types of pulsed operations: pulsed voltage and pulsed current. Pulsed voltage operation drives the electrolysis reaction by applying periodic voltage fluctuations. Variations in amplitude and frequency can affect the electric field distribution on the electrode surface, thereby regulating ion migration and reaction kinetics. Pulsed voltage offers a simple control method and is typically used in scenarios requiring rapid voltage adjustments to optimize reaction conditions. In contrast, pulsed current operation adjusts the electrolysis process by periodically varying the intensity and duration of the current, allowing precise control of current density and reaction rates. Pulsed current is particularly suitable for regulating the local current density of electrochemical reactions, optimizing electrolysis efficiency, and reducing electrode polarization effects. Both pulsed methods are widely applied in WE, metal electrodeposition, and other electrochemical reactions. They promote efficient electrolysis in different ways, reduce energy consumption to some extent, and enhance the long-term stability of the system. In practical applications, selecting the appropriate pulsed method requires optimization based on the characteristics of the electrolysis system and the requirements of the target product.

#### Pulsed Voltage

During pulsed WE, the voltage typically varies from the baseline potential (*V*_B_), representing the reference or steady-state value, to the peak potential (*V*_E_), representing the peak or final value. The pulsed period is the sum of *t*_on_ and *t*_off_, where the duty cycle is defined as the ratio of *t*_on_ to the pulsed period T, and the frequency is the reciprocal of the pulsed period. The pulsed amplitude is the difference between the baseline potential and the terminal potential. The resulting current consists of the Faradaic current (*I*_F_, when the peak current falls within the potential range for WE) and the non-Faradaic current (*I*_NF_, which primarily accounts for the charging and discharging of the EDL). By adjusting the operating parameters of the pulsed shape, including waveform, amplitude, and duty cycle, multi-factor variable WE can be achieved. The duty cycle can be defined using PWM [137]. Moreover, providing an appropriate *V*_B_ during electrolysis can sustain hydrogen production while preventing interruptions in ion mass transfer within the electrolyte, thereby further reducing concentration polarization. Common PDE experimental parameters are expressed using ton and toff, where *D* is the pulsed duty cycle and *f* is the frequency (Eq. 1). The definition of pulsed voltage (*V*_Pulse_) is shown in Eq. 2:

1 $$D = \frac{{T_{on} }}{{T_{on} + T_{off} }} = T_{on} \cdot f$$

2 $$V_{Pulse} = V_{E} \cdot D + V_{B} \cdot \left( {1 - D} \right)$$

**Fig. 14:**
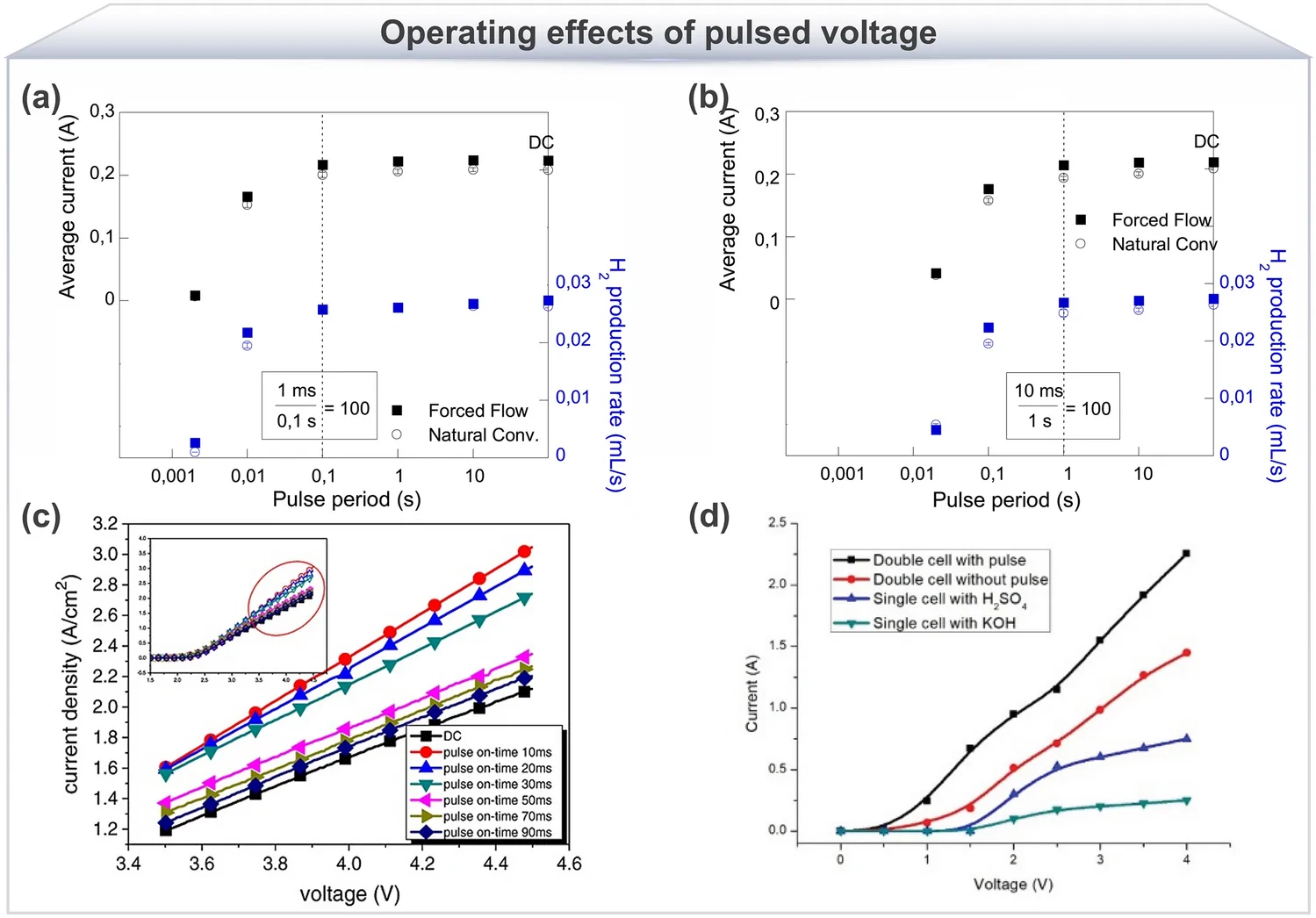
Operating effects of pulsed voltage. Off-time of **a** 1 ms and **b** 10 ms under natural convection and forced flow, showing the relationship between average current and hydrogen production rate with pulsed voltage period. Reproduced with permission from Ref. [138]. Copyright 2021, Elsevier. **c** *I*-*V* curves under different pulsed on-times and CE conditions. Reproduced with permission from Ref. [109]. Copyright 2013, Wiley–VCH. **d** *I*-*V* curves of single-electrode and dual-electrode cells with and without pulsing [110]. Copyright 2019

In further exploring the effect of pulsed voltage on the WE process, many researchers have conducted in-depth experiments and analyses from different perspectives. Rocha et al. investigated the influence of pulsed voltage on 3D Ni electrodes in AWE. The purpose of applying pulsed voltage was to promote bubble removal, as bubbles that are trapped in large-pore open structures can reduce the effective surface area of the electrodes and increase the ohmic resistance [138]. Using a potentiostat to apply square wave pulses, they analyzed the current change under a 50% duty cycle for the first pulsed voltage. The results showed that when the pulsed period was 100 ms, the baseline voltage was 0 V, and the peak voltage ranged from 3.5 to 4.5 V, the on-state current increased by 10%-14%. Further research indicated that within duty cycle range of 50%-99.9%, with fixed pulsed off-times of 1 ms (Fig. 14a) and 10 ms (Fig. 14b), pulsed voltage could approach the CE hydrogen production rate, with FE as high as 98%. Lower duty cycles enhanced the forced flow effect. Moreover, a synergistic effect existed between the 3D Ni electrodes and pulsed voltage, with higher on-state and off-state currents providing better performance for short pulses. When the pulsed width was 1 ms, the average current was more than twice as high as that with a 100 ms pulse, indicating that pulsed parameters significantly regulate electrolysis performance. Based on the above research, Demir et al. further utilized transistors to generate voltage pulses and validated and supplemented the advantages of pulsed voltage in improving WE performance [102]. They emphasized that pulsed voltage not only enhances electrolysis performance by increasing the active surface area of the electrodes but also reduces anode corrosion and improves bubble mass transfer efficiency, effectively reducing the energy consumption of WE. Their experiments showed that when the pulsed frequency reached 1200 kHz, the electrolysis energy consumption could be reduced by 20%-25%. Correspondingly, Lin and Hourng conducted experiments to explore the specific effects of different pulsed parameters on the WE process, further enriching research in this field [109]. The use of pulsed voltage rapidly increased the current (Fig. 14c), which not only accelerated the detachment of bubbles from the electrode surface but also increased the mass transfer rate in the electrolyte, effectively reducing the electrochemical polarization of the diffusion layer, thus improving hydrogen production efficiency. Setting the pulsed duty cycle to 10% and the ton to 10 ms, the current density increased by 680 mA cm^−2^ compared to CE. Meanwhile, the electrolysis energy consumption was reduced by 88% compared to CE. The shorter the pulsed time, the larger the current density, and as the pulsed frequency increased, the electrolysis efficiency significantly improved. These results indicate that pulsed voltage has significant advantages in improving electrolysis performance, especially in terms of increasing hydrogen production and reducing energy consumption. Five years later, Hourng et al., building on previous studies, attempted to apply pulsed voltage to a dual-electrode (acid–base) WE system to further explore its potential advantages. They innovatively used PEM and dual-electrolyte system, discovering that when dual-electrode and dual-electrolyte system was employed, the electrolysis voltage could be reduced to 0.7 V, compared to 1.23 V for a single electrolyzer. In addition, the higher the baseline voltage of the pulse, the lower the on-state current, and the higher the H_2_ production rate [110] (Fig. 14d). In summary, pulsed voltage increases the current during the on-state, but the average current decreases, leading to a decline in hydrogen production rate. This phenomenon is attributed to the fact that during part of the hydrogen production process (i.e., the off-time), the voltage is zero, which affects the current transport and hydrogen production. To explore the microscopic mechanism of pulsed voltage in WE, Vanags et al. designed a new type of pulsed generator power supply to apply induced voltage during electrolysis, combining micro-sensors and mass spectrometers to conduct quantitative and qualitative analysis of the gases. They also investigated the solvation relaxation mechanism of electrons emitted from the cathode in the electrolyte. Their study revealed the capacitor-like behavior of the electrolyzer under short pulsed voltage and the separation phenomenon between the EDL charging process and the Faradaic reaction [139]. This suggests that the EDL capacitive effect induced by short, high-voltage pulses is independent of the electrolyte concentration, while the dynamics of the subsequent longer discharge process are influenced by the electrolyte concentration.

The pulsed voltage operating mode not only enables the efficient detachment of bubbles and a significant increase in electrode active surface area, but also effectively reduces energy consumption and mass transfer losses during the electrolysis process. Additionally, by integrating a dual-electrode system and innovative power supply design, the application of pulsed voltage has expanded from optimizing macroscopic performance to analyzing microscopic mechanisms, providing new insights into the separation of EDL charging and Faradaic reactions in the electrolyte. However, there is still a need to develop efficient pulsed power supplies suitable for industrial-scale applications to meet the demands of large-scale hydrogen production.

#### Pulsed Current

When applying pulsed current (i.e., intermittent current), ions near the electrode participate in the reaction, forming a concentration gradient at the electrode/electrolyte interface. During the pulsed intervals, the diffusion process replenishes the ions consumed by the reaction. This mechanism effectively reduces polarization phenomena, achieving higher current efficiency at a fixed potential and thereby enhancing hydrogen production efficiency [109]. Therefore, further research can investigate how the response of pulsed current in WE stacks affects hydrogen production under pulsed voltage and analyze the differences compared to conventional constant current electrolysis. Kim et al. described the differences between constant current and pulsed current using Eq. (3) and defined the growth rate of pulsed current with Eq. (4) [113]. From a theoretical perspective, they further explored the contribution of pulsed current to hydrogen production efficiency, providing new insights into its potential applications.

3 $$\Delta I \to \left\{ {\begin{array}{*{20}c} {\Delta I_{E} = I_{pulseH} - I_{CE} } \\ {\Delta I_{B} = I_{CE } - I_{pulseL} } \\ {\Delta I_{avg} = \frac{{\Delta I_{E} + \Delta I_{B} }}{2}} \\ \end{array} } \right.$$

4 $$\eta_{pulseI} = \left( {\frac{{\Delta I_{avg} }}{{I_{CE} }}} \right)\%$$

where *I*_pulseH_ is the high peak value of the pulsed current, *I*_pulseL_ is the low peak value of the pulsed current, and *η*_pulseI_ is the increase rate of the pulsed current density.

**Fig. 15:**
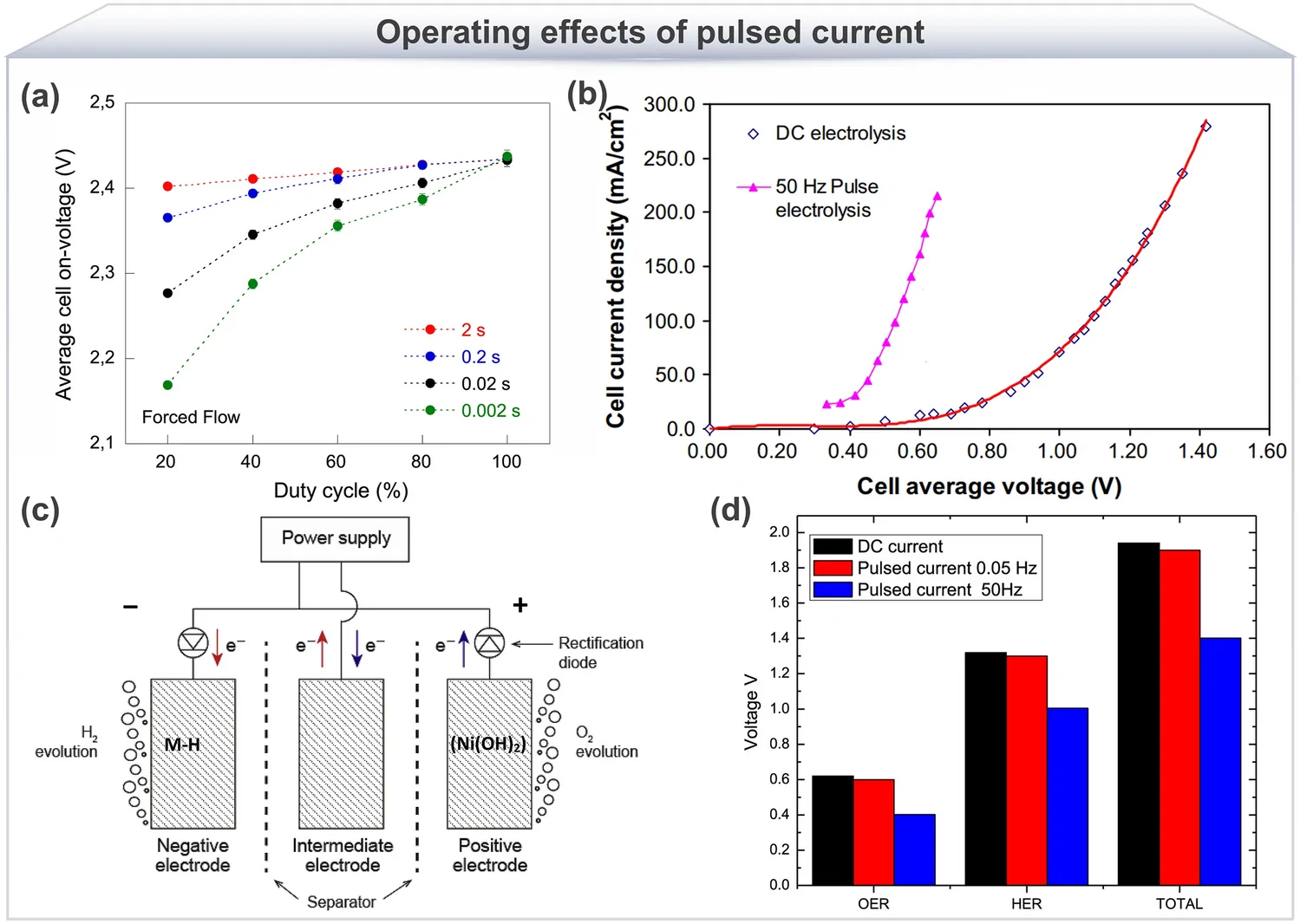
Operating effects of pulsed current. **a** Relationship between the average conduction voltage of pulsed current and duty cycle under different pulsed periods (peak current 0.3 A, forced flow). Reproduced with permission from Ref. [138]. Copyright 2021, Elsevier. **b** Relationship between cell voltage and current density for CE and 50 Hz PDE (cell temperature 75 °C, 2.0 M (NH_4_)_2_SO_3_). Reproduced with permission from Ref. [128]. Copyright 2013, Elsevier. **c** Schematic of the WE cyclic pulsed current supply, where the MnO_2_ intermediate electrode forms a closed circuit for HER and OER. **d** Comparison of pulsed current electrolysis at 0.05 Hz and 50 Hz with conventional CE at 0.05 A cm^−2^. Reproduced with permission from Ref. [108]. Copyright 2018, Elsevier

In the aforementioned studies, PDE has been proven to significantly enhance electrolysis efficiency, especially in optimizing pulsed current parameters such as duty cycle and pulsed period. Based on this, Rocha et al. further investigated the role of pulsed current with a 50% duty cycle in AWE using 3-D Ni electrodes, aiming to distinguish the specific effects of pulsed width and duty cycle when both pulsed voltage and current are applied [138]. They found that when 50% duty cycle was applied and a pulsed current of 20 ms was increased from 0 to 0.1 A cm^−2^, the cell voltage decreased by 45 mV. Additionally, they noted that the statistical effect of pulsed current is particularly significant when the duty cycle is below 50%, especially under conditions of short pulsed periods and low-duty cycles (10%–20%), where electrolyzer performance improved. Under 20% duty cycle and 2 ms pulsed period, the overpotential of the electrolyzer decreased by 260 mV (~ 28%), while under 50% duty cycle, the overpotential only decreased by 17% (Fig. 15a). Therefore, pulsed currents with short pulsed periods (on the millisecond scale) and low-duty cycles (10% to 20%) may represent a highly promising strategy for improving the performance of AWE electrolyzers. Based on this, the profound effects of PDE specific characteristics on electrode surface properties have garnered extensive attention from researchers. Hristova et al. conducted an in-depth analysis of the effects of PDE characteristics (duty cycle and frequency) on the electrochemical properties of AISI 316 stainless steel electrode materials. Experimental results showed that the formation of a Cr(OH)_3_ deposit layer on the cathode surface under PDE conditions. While this layer did not substantially hinder the transport of reactive ions, it did lead to a reduction in electronic conductivity. Meanwhile, PDE altered the Cr content and defect density in the anodic surface film, thereby affecting both electronic and ionic conductivity [140]. This study revealed the complexity of electrode surface modification induced by PDE processes and further highlighted that these modifications are closely related to changes in pulsed duty cycle and frequency, with the number of cathodic crystals increasing as pulsed duty cycle and frequency increase. The development of PDE has further expanded its application scope, particularly in the electrolysis of (NH_4_)_2_SO_3_ aqueous solutions. Due to the high solubility of (NH_4_)_2_SO_3_ and (NH_4_)_2_SO_4_, Huang suggested that high-concentration solutions (> 2.0 M) can be used for electrolysis. In this context, the mass transfer rate of SO_3_^2−^ or SO_4_^2−^ ions at the anode surface becomes the rate-controlling step of the electrolysis process [128]. However, PDE, by intermittently applying cell voltage, effectively controls the electrolyzer current to match the mass transfer rate, thereby improving electrolysis efficiency. Results showed that when the average pulsed cell voltage was 0.60 V (with a peak cell voltage of 1.20 V), the electrolyzer current reached 180 mA cm^−2^. Under CE, achieving the same current density required a cell voltage of approximately 1.26 V, indicating that PDE could save 50% of the energy. Moreover, at cell voltage of 1.16 V, when the pulsed frequency increased from 10 to 50 Hz, the current density rose from 50 to 130 mA cm^−2^ (Fig. 15b), further demonstrating the advantages of PDE in enhancing electrolysis efficiency. Based on these studies, Vincent et al. further validated the specific role of pulsed current in improving electrolysis efficiency, particularly in the hydrogen production cycles of water splitting using 3D-MnO_2_ electrodes (Fig. 15c). By decoupling the hydrogen evolution cycle (HEC) and oxygen evolution cycle (OEC) of AWE, they found that increasing the pulsed frequency significantly improved the performance of both HEC and OEC (Fig. 15d) [108]. This is because the short action time of high-frequency pulses prevents the diffusion layer thickness from reaching a steady state, thereby ensuring the rapid recovery of reactant concentrations on the electrode surface, ultimately improving electrolysis efficiency. Results showed that under conditions of 25 °C, a current density of 0.2 A cm^−2^, and pulsed frequency of 500 Hz, the cell voltage required for PDE was only 1.69 V, significantly lower than the voltage required for conventional AWE, further demonstrating the energy efficiency advantages of PDE.

In short, PDE not only enhances the electrochemical performance of electrode surfaces but also significantly improves the efficiency of WE and reduces energy consumption, demonstrating great potential, particularly in optimizing electrolysis conditions such as pulsed frequency and duty cycle.

### Influencing Factors

#### Frequency

In electrochemical processes, the electrical behavior and characteristics of the process significantly affect efficiency. Understanding the electrode/interface current behavior from an electrical engineering perspective is crucial for the development of PDE, particularly for reducing the overall system impedance. During experiments, Kim et al. performed electrical modeling of PDE based on real-time data and derived the frequency limitation range for applied pulsed frequencies, which is governed by the RC time constant law. They found that as the frequency of the electric field increases, the polarization effect of the electrode gradually diminishes, thereby increasing hydrogen production. However, the frequency cannot be increased indefinitely, as its upper limit is constrained by the electrode/interface response time, particularly determined by the slowest response time of current within the EDL or diffusion process. The charging effect of the EDL limits the accumulation of charge, and as a result, the increase in frequency is also influenced by the capacitor time constant. Therefore, when the frequency is too high, the capacitive effect cannot further enhance reaction efficiency and may even lead to a decrease in system performance. Based on frequency limitations, PDE hydrogen production experiments were conducted in PEM cells, and the results showed that at frequencies below 20 Hz, hydrogen production increased by as much as 5.79% (operating parameters 8.9 V/23 A) [113]. Their findings provide valuable insights for further optimizing PDE systems, but some limitations still exist. While the effect of the electric field frequency on polarization effects is well understood, the influence of capacitive effects on current response and hydrogen production rates still requires further validation through additional experimental data. Therefore, future research should further explore the coupling relationship between the EDL process and diffusion effects under different operating conditions, especially under high-frequency pulses, and optimize the combination of pulsed frequency and electric field strength to achieve more efficient hydrogen production.

In the field of PDE for hydrogen production, an increasing amount of research focuses on the effect of pulsed parameters on electrolysis efficiency and hydrogen production. For example, Al-Hasnawi et al. studied the effects of pulsed waveform, amplitude, and frequency on WE and experimentally determined the optimal pulsed frequency and waveform to optimize electrolysis energy efficiency. In their study, the pulsed frequency ranged from 2 Hz to 1 MHz, with amplitudes ranging from 1.5 to 3 V, and the waveforms included square, sine, and triangular waves [141]. The results showed that at specific frequencies, square waves were the only waveform that exhibited significant hydrogen production and the highest hydrogen production efficiency. However, this optimal frequency was derived from their experimental conditions, and adjustments would be necessary when applied to other system studies. As the theoretical and experimental research on PDE in WE continues to deepen, most studies have shown that compared to conventional CE, PDE can significantly improve energy efficiency, reduce energy consumption, and promote more hydrogen generation, bringing new technological breakthroughs to the field of WE. Radigues et al. studied the enhanced effect of combining 3D electrodes with pulsed voltage and forced electrolyte flow in AWE [133]. In their experiments, the peak voltage of the electrolyzer did not surge from 1.2 to 3.0 V, but increased stepwise from 1.2 to 3.0 V in 100 mV increments with a duty cycle of 50%. By applying different frequencies (2.5, 25, and 250 Hz) and pulsed widths (2, 20, and 200 ms) of pulsed voltage, they investigated the effects of these parameters on the performance of the electrolyzer. The experimental results demonstrated that using a 2 ms high-frequency square wave pulsed voltage significantly improved hydrogen production efficiency. Furthermore, there was a synergistic effect between the 3D electrode, forced flow, and pulsed voltage, which further enhanced electrolysis performance. However, in contrast to the aforementioned viewpoint, Dobó et al. presented a different perspective on the effects of high-frequency pulses. They explored the effect of high-frequency pulses on electrolyzer performance by increasing the frequency on a logarithmic scale. They found that, compared to CE, PDE could, under certain conditions, reduce the overall efficiency of the electrolyzer [142]. It is worth noting that their research mainly focused on the logarithmic scale range and did not fully test the effects of high-frequency pulses (above 10 Hz). This suggests that high-frequency pulses may improve electrolyzer efficiency, but their experimental results did not cover this range. This finding indicates that different electrolyzer system configurations may have significantly different pulsed parameter requirements.

Although PDE has shown significant advantages in enhancing hydrogen production efficiency, optimizing the key parameters of pulses remains crucial for improving electrolysis performance. Additionally, the configuration requirements of different systems may lead to variations in the effectiveness of PDE. Therefore, future research should delve deeper into exploring the optimal parameter combinations of PDE under various conditions, particularly the coupling relationship between the EDL process and diffusion effects under high-frequency pulses, thereby providing more comprehensive guidance for optimizing WE hydrogen production technology.

#### Duty Cycle

The adjustment of pulsed duty cycle can replace the pulsed interval time by setting control parameters, thereby improving the efficiency of the electrolysis process. Selecting an appropriate pulsed duty cycle can effectively regulate how bubbles freely escape from the electrode/solution interface during electrolysis, preventing excessive bubble accumulation and the formation of additional bubbles while ensuring that the electrode surface can reach a refreshed state before the next pulsed cycle begins. However, some studies suggest that merely extending the pulsed interval time does not significantly improve electrolysis efficiency and may even lead to decrease in efficiency. The optimal pulsed interval time should be sufficient for most bubbles on the electrode surface to detach. Meanwhile, the minimum pulsed interval time is usually limited by bubble size and coverage, with the applied current being a critical factor affecting this parameter [143].

**Fig. 16:**
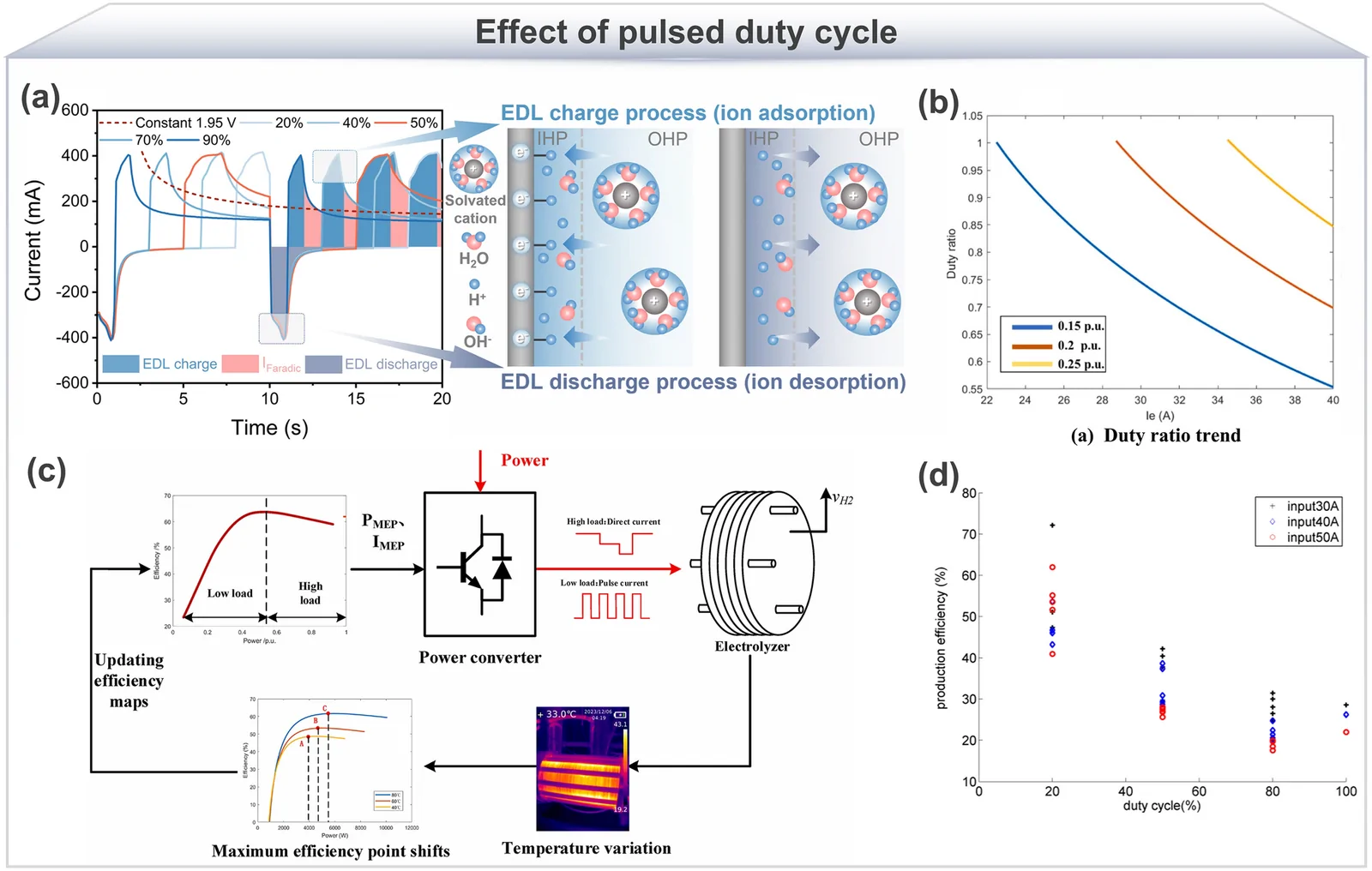
Effect of pulsed duty cycle. **a** Hydrogen production current response curves and ion adsorption/desorption of non-Faradaic processes under different duty cycles in PEMWE. Reproduced with permission from Ref. [55]. Copyright 2025, Elsevier. **b** Variation trends of duty cycle with pulsed current amplitude under different average input power levels. Reproduced with permission from Ref. [144]. Copyright 2024, Elsevier. **c** Strategy for determining the optimal duty cycle of voltage in AWE at different temperatures. Reproduced with permission from Ref. [145]. Copyright 2024, Elsevier. **d** Effect of duty cycle on production efficiency [143]. Copyright 2016, EDP Sciences

In our previous work, the use of PDE successfully overcame the issue of proton mass transfer limitation caused by deteriorated mass transport in PEMWE. It was also found that adjustments to the pulsed duty cycle have a significant effect on PEMWE reactions. Specifically, the ion adsorption and desorption behavior at the electrode/solution interface, triggered by EDL charging and discharging, may significantly affect the charge transfer rate between OH^−^ and H^+^ on the electrode surface and in the electrolyte [55] (Fig. 16a). This process could become the rate-limiting step in electrolysis. By regulating the pulsed duty cycle, not only was electrocatalytic activity dynamically reconstructed, but the ion diffusion and mass transport processes were also optimized, achieving a balanced match between the two and effectively enhancing the hydrogen production rate. When exploring the effect of pulsed voltage on AWE energy efficiency, Demir et al. investigated the changes in energy consumption during WE under different duty cycles and frequencies using a specially designed square wave pulsed voltage circuit [102]. They found that, in CE mode, a current density of up to 3.7 A cm^−2^ could be achieved at 10% and 15% KOH concentrations, while in PDE mode, the current density at the same concentration increased to approximately 4.04 A cm^−2^. With pulsed potential of 6 V, duty cycle of 50%, and frequency of 1200 kHz, energy consumption was reduced by 20% to 25%. Furthermore, with duty cycle of 10%, the optimal frequency was around 140–200 kHz, while at 50% duty cycle, the optimal frequency was 380–400 kHz. To further investigate the role of pulsed current in improving AWE efficiency, Cheng et al. proposed a method to address low efficiency under low loads through pulsed current electrolysis. Pulsed current effectively reduced energy losses caused by parasitic currents, significantly improving hydrogen production efficiency [144]. As shown in Fig. 16b, the pulsed current duty cycle plays a critical role in efficiency improvement, particularly in the relationship between the duty cycle, EDL charging time, and hydrogen production time. By reducing the pulsed duty cycle and increasing the pulsed amplitude, the EDL charging time is shortened, and hydrogen production time is extended, effectively enhancing electrolysis efficiency. In the same year, Cheng et al. further proposed a multi-mode adaptive control strategy (Fig. 16c) to address efficiency drops caused by temperature fluctuations in the electrolyzer due to power fluctuations [145]. This strategy flexibly selects CE or PDE based on input power and electrolyzer temperature to optimize hydrogen production efficiency in AWE systems. Specifically, under high load conditions, the electrolyzer operates with CE, while under low-load conditions, it uses pulse current and intermittently operates at maximum efficiency at low frequencies. Experimental results showed that in a 10 kW AWE system, the efficiency of the electrolyzer increased from 21.54 to 42.73% at 15% rated load and 80 °C. Furthermore, this method expanded the operating range of the electrolyzer, increasing the range from 30% to 100% at minimum efficiency of 55% to 21%–100%. The core of this method lies in optimizing the design and control technology of converters in electrical engineering to regulate the pulsed electric field and identify the optimal pulsed duty cycle to address the aforementioned issues. However, during the process of identifying the optimal pulsed duty cycle, reverse corrosion currents may occur, potentially having adverse effects on the lifespan of the electrocatalysts and membranes in the electrolyzer. Therefore, future research should focus on balancing hydrogen production efficiency with electrolyzer durability, particularly in pulsed parameter control, ensuring that optimized strategies can effectively enhance efficiency while extending the service life of equipment. To further study suitable pulsed interval cycles that allow bubbles to rise freely without generating additional bubbles, Vilasmongkolchai et al. combined mathematical modeling and acoustic emission technology to analyze bubble rise velocities [143]. Lower frequencies and longer pulsed interval cycles were found to promote free bubble rise, but longer interval cycles led to reduced hydrogen production efficiency (Fig. 16d). Similarly, when the pulsed duty cycle increased, hydrogen production efficiency also decreased. Therefore, by controlling a low-frequency and low-duty-cycle pulse activation mode, hydrogen production efficiency during the electrolysis process can be significantly improved.

From the perspective of pulsed duty cycle, appropriately adjusting the pulsed amplitude and frequency, particularly by precisely controlling the pulsed width (i.e., duty cycle), may be key to improving electrolysis efficiency. Higher frequencies and lower peak voltages could help reduce energy losses during the electrolysis process while enhancing current density and bubble release efficiency. Optimizing the pulsed width may significantly affect the dynamic behavior of the electrolysis process, especially in terms of charge transfer, bubble generation, and product formation within the electrolyzer. Therefore, future research should focus on the interrelationships between pulsed duty cycle, frequency, and pulsed width, exploring how precise regulation of these parameters can further enhance electrolysis efficiency and understanding their long-term effect on the electrolysis process.

#### Amplitude

Considering the effect of molecular dynamics, the application of pulsed voltage may enhance WE efficiency by altering the dynamic behavior of water molecules. Part of the reason PDE improves WE efficiency may due to its regulation of the internal structure and intermolecular interactions of water molecules. By gaining a deeper understanding of the molecular dynamics of water, it is possible to better select and apply pulsed voltage amplitudes to optimize the electrolysis process. For example, applying a pulsed electric field can adjust the ratio of ortho- to para-isomers in water, thereby reducing the energy required to dissociate water molecules. Moreover, the strength of hydrogen bonds within and between water molecules plays a crucial role in the energy conversion during electrolysis [146–148]. Generally, hydrogen bonds between water and air or between water molecules are weaker, whereas those between water and surfaces are stronger [149, 150]. These differences can significantly affect the efficiency of the electrolysis process. At the molecular level, understanding the role of pulsed amplitude reveals that pulsed voltage may excite vibrational modes within water molecules, altering the hydrogen bond structure. This change could destabilize hydrogen bonds, making water molecules easier to dissociate and thereby reducing the energy required for electrolysis. Additionally, the modulation of water molecule dynamics by pulsed voltage amplitude could influence the conductivity of water, contributing to reduced energy consumption of the system [9, 151]. Future studies should focus on further exploring the effect of pulsed voltage amplitude on the molecular dynamics of water, particularly the changes in the hydrogen bond network under different amplitudes and how these changes affect electrolysis efficiency. Simultaneously, research should aim to develop more precise control methods to meet energy efficiency demands and further enhance the overall efficiency of WE.

**Fig. 17:**
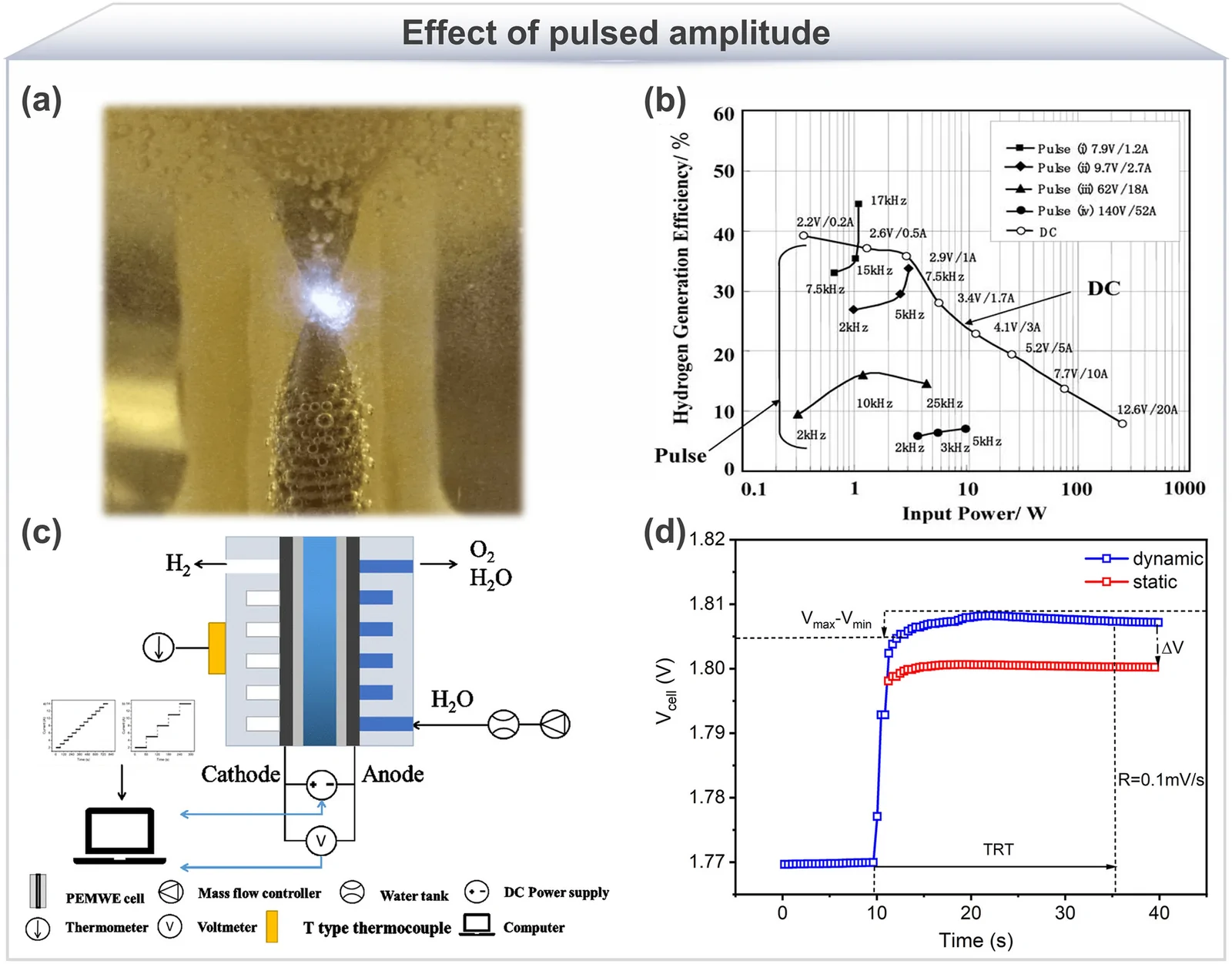
Effect of pulsed amplitude. **a** Plasma phenomenon formed in the electrolyzer due to pulsed voltage amplitude [152]. Copyright 2016, Open access. **b** Relationship between hydrogen production efficiency and input power under PDE and CE conditions. Reproduced with permission from Ref. [96]. Copyright 2005, Springer Nature. **c** Schematic diagram of the experimental measurement setup for pulsed response voltage amplitude. **d** Typical response behavior of PEMWE device. Reproduced with permission from Ref. [153]. Copyright 2023, Elsevier

The effect of pulsed voltage amplitude on the molecular dynamics of water can break through the conductivity limitations of water, leading to plasma electrolysis. Albornoz et al. explored the effects of power converter parameters and electrolyzer configurations on the electrolysis process from an electrical perspective. They claimed that when the power supply voltage is 10 V, the frequency is approximately 500 kHz, and the electrode spacing is 0.1 mm, the induced pulsed voltage amplitude can reach 430 V, resulting in arc discharge phenomena near the electrodes [152] (Fig. 17a). This process promotes the ionization of the gas medium, and as the electrode spacing decreases, plasma formation is successfully achieved. Specifically, when the pulsed voltage amplitude exceeds about 500 V (for electrode spacings less than 0.1 mm), the arc discharge becomes more pronounced, and plasma formation becomes more feasible as the electrode spacing further increases. From a molecular dynamics perspective, the high pulsed voltage amplitude activates the electrons and ions in water molecules, intensifying the ionization phenomenon and promoting plasma formation, thus enhancing electrolysis efficiency. However, the optimization methods for plasma electrolysis efficiency based on different electrode configurations and frequencies were not further discussed.

Additionally, Shimizu et al. proposed a novel electrolysis method for hydrogen production based on an ultra-short pulsed power source, composed of static inductive thyristors (SIThy) and inductive energy storage (IES) circuits. In experiments using 1 M KOH electrolyte and Pt electrodes with an electrode spacing of 3 cm, ultra-short pulses with a width of 300 ns, frequency of 2–25 kHz, and peak voltage of 1.9–140 V were applied. The experimental results showed that the hydrogen production rate significantly decreased under CE, whereas during the 300 ns pulsed width electrolysis, the hydrogen production rate did not decrease when the pulsed peak voltage was 62 or 140 V and even increased at 1.9 and 9.7 V. Particularly, when the peak voltage was 7.9 V and the frequency was 17 kHz, the hydrogen production rate surpassed that of CE [96] (Fig. 17b). They attributed this phenomenon to the ultra-short pulsed duration (300 ns), which is much shorter than the time required for the diffusion layer to form (~ 3 μs). Under these conditions, the electrolysis process mainly proceeds through electron transfer, avoiding the formation of the diffusion layer, allowing electrons to combine more quickly with H^+^, thus improving the hydrogen production rate. Furthermore, the pulsed duration is shorter than the time required for the formation of the EDL (on the order of μs ~ ms), thus avoiding current losses generated during the EDL charging and discharging process. To further enhance electrolysis efficiency, Gong et al. conducted a systematic characterization study on the voltage response over time for a single PEMWE device under different pulsed currents (Fig. 17c). They used three comprehensive performance parameters—total response time (TRT), voltage stability (*V*_max_–*V*_min_), and voltage difference under PDE and CE conditions (Δ*V* = *V*_dynamic_–*V*_static_) to determine the response rate, stability, and energy consumption of the PEMWE device [153] (Fig. 17d). Experimental results showed that the voltage response was optimal at low currents and small step current amplitudes (Δ*I* = 1 A), with low-energy consumption, short response time, and good response stability. Increasing the step current amplitude reduced the voltage stability of the electrolyzer. The internal resistance of the PEMWE device changes over time, and pulsed operation has a significant effect on resistance. The joule heat generated positively affects the response behavior, but increasing the step current amplitude (Δ*I* = 3 A) or conducting square wave durability tests would impair the response behavior of the PEMWE device.

In short, during the application of PDE, disturbances caused by higher voltage or current pulsed amplitudes may lead to a transition in the electrochemical system's response characteristics from linear to nonlinear, a phenomenon similar to the changes observed in EIS technology [13]. The application of different pulsed amplitudes affects the control process of reaction kinetics, particularly the charge transfer coefficient. For reactions with a higher charge transfer coefficient, periodic PDE conditions generally perform significantly better than CE conditions, thereby improving the FE of the products. This regulatory effect helps optimize the electrolysis process, enhancing the reaction rate and electrolysis efficiency.

### Key Limiting Factors

In the pulsed WE process, the performance of the stepwise reactions can vary significantly with changes in time scale (such as reaction rate) and spatial scale (such as reaction location). One key challenge is how to coordinate the rates of the various stepwise reactions to optimize electrolysis efficiency. Liu et al. showed that during pulsed WE, the rapid variation in current can significantly affect the overall system performance due to inductive reactance losses [19]. Therefore, optimizing pulsed voltage parameters and reducing inductive losses have become important approaches to improving electrolysis efficiency. However, despite the good performance of PDE in experiments, the effect of oxidation/reduction cycling in the PDE process on other components of the electrolyzer, such as membranes and electrodes, has not been thoroughly explored. Specifically, the potential new degradation mechanisms of electrodes during oxidation cycles remain an area of preliminary research [154]. Therefore, future research should focus on the complex reaction mechanisms in PDE, particularly on catalyst reconstruction, dynamic structure capture, and changes in the microenvironment under reaction conditions. These studies will require the use of more in situ techniques and operational technologies to more precisely reveal the actual reaction mechanisms in PDE.

To better understand the reaction mechanisms of PDE, it is necessary to combine multiple in situ/visualization techniques and systematically track the micro-nanoscale ionic behaviors at the electrode/solution interface under PDE conditions. Recent dynamic evolution visualization studies have significantly enhanced our understanding of the PDE enhancement mechanism. However, to further reveal the mechanisms of PDE, more micro-nanoscale visualization techniques are needed, especially to push the measurements toward higher current densities and industrial-scale system architectures. Currently, most PDE studies are conducted under relatively low current densities and model electrochemical electrolyzer setups. Therefore, future research should be expanded to focus on higher current densities and system configurations that are closer to industrial applications.

Additionally, there are practical challenges in the operation of pulsed WE. Due to differences in transient measurement parameters and equipment between different laboratories, the consistency of experimental results is often low. Moreover, when using the gas collection method, issues such as pressure drop can occur when the pulsed frequency exceeds 0.5 Hz, especially for hydrogen collection tubes with a diameter of 2 mm, which can be affected by atmospheric pressure differences. The *E*_off_ phase may also bring negative effects, as the current polarity reversal during this phase could accelerate the corrosion of the electrolysis system, increasing internal losses in the electrolyzer. Finally, the universality of pulsed frequency and duty cycle also cannot be ignored.

In terms of optimizing PDE, in addition to precise control over the choice of pulsed frequency and duty cycle, exploring more complex operational strategies could yield better performance. For instance, using more than two set voltages or employing more complex waveforms, such as sine waves, may further enhance reaction efficiency. Different types of catalysts respond differently to pulsed frequency and duty cycle, thus optimized pulsed parameters need to be developed for different catalyst systems. Although some experimental results have been achieved, there is still significant potential to be explored to maximize the application potential of PDE. It is worth noting that while pulsed WE technology has great development potential, there are also some cost challenges associated with its application. Particularly for high-frequency pulsed electric field systems that require high power, the equipment costs will increase significantly with the increasing power demand. Maintaining the pulsed waveform is also an issue, especially when using square wave pulses, where this problem becomes more prominent as the power increases. However, with advancements in electrical component technology, the cost of equipment may gradually decrease, eventually making the economic feasibility of pulsed electric fields in industrial-scale applications achievable [155].

In summary, although pulsed WE technology faces several key challenges, its potential in reaction kinetics, catalyst reconstruction, micro-reaction mechanisms, and electrolysis efficiency remains enormous. Future research should focus on how to optimize PDE parameters under different catalysts and reaction conditions, explore new experimental techniques to reveal the fundamental mechanisms of PDE, and address cost and equipment challenges in industrial applications.

## Renewable Energy-Driven WE

In renewable energy-driven hydrogen systems, one of the key applications of PDE is to transform the fluctuations in power output into opportunities for improving hydrogen production efficiency. By dynamically regulating the current and voltage in a controlled manner, PDE can adapt to irregular power fluctuations caused by factors such as day-night cycles or cloud shading in off-grid photovoltaic (PV) systems, thereby ensuring stable electrolysis operation and consistent hydrogen yield. Under real sunlight conditions, further integration with a pulsed generator to control the frequency and duty cycle of the response voltage allows optimization of energy use during power fluctuations, minimizing unnecessary energy consumption, and maintaining high electrolysis efficiency even when PV output is insufficient, thus enhancing overall system performance.

### Key Technologies for Fluctuating Power

Efficient renewable energy-driven WE must overcome a series of technical challenges arising from the fluctuating power supply. These challenges include the narrow load adjustment range and slow response rate of electrolyzers, the risk of hydrogen–oxygen mixing in low-load regions, and the accelerated aging of core components due to frequent fluctuations. During long-term operation, it is crucial to effectively coordinate the input and output of the fluctuating renewable energy-driven electrolysis system [156, 157]. Specifically, in the process of green hydrogen production, how to regulate current to keep power fluctuations within a reasonable range and how to assess the adaptability of the electrolyzer stack and system to the green energy grid environment are key issues that need to be addressed. In the future, we must overcome these technical challenges to break through the bottlenecks in electrolysis-based hydrogen production and achieve a sustainable energy transition.

**Fig. 18:**
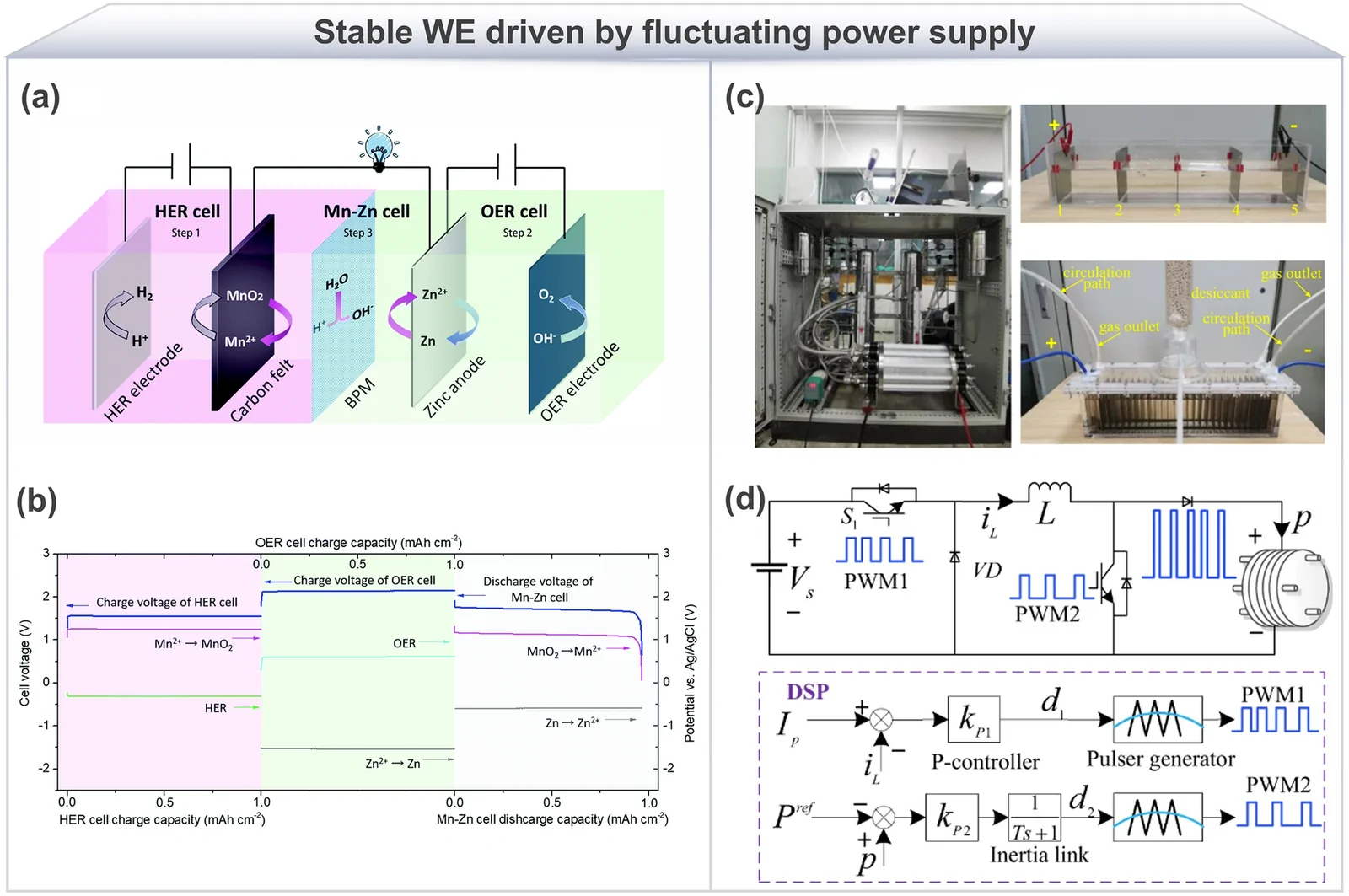
Stable WE driven by fluctuating power supply. **a** Working principle of the integrated system of bifunctional WE and Mn-Zn battery. **b** HER and OER battery charging potential curves and Mn-Zn battery discharge potential curve [159]. Copyright 2021, Royal Society of Chemistry. **c** Photographs of the 10 kW commercial AWE (left) and the 3 kW laboratory-scale test electrolyzer (right). **d** Structure of the multi-mode self-optimizing electrolyzer converter and its control method under low-load conditions. Reproduced with permission from Ref. [160]. Copyright 2023, Springer Nature

Under high fluctuating power input, WE systems face a conflict with the rapid response requirements of wind and solar power sources, resulting in long-term instability in the operation of wind-solar-powered electrolysis systems. To achieve stable process integration and control between fluctuating power sources and electrolysis systems, two main approaches can be considered. On the one hand, from the perspective of wind-solar fluctuating power sources, the primary objective should be to meet user demands while maximizing the consumption of wind and solar energy. Valenciaga et al. used a supervisory control system to determine the system's operating mode based on the energy balance between wind power generation and total demand [158]. They employed a higher-order sliding mode design, allowing a low-level controller to maintain smooth operation even when subjected to external input power disturbances. On the other hand, for the electrolysis system itself, it is essential to achieve stable operation under high fluctuating power input. Through in-depth exploration of different electrolytic media and electrolytic equipment, Huang et al. proposed an innovative decoupling integrated system combining bipolar WE and Mn-Zn batteries. This system introduced MnO_2_/Mn^2+^ as an auxiliary medium to effectively optimize the hydrogen production process and improve system stability [159] (Fig. 18a). This design enables the electrolysis system to adapt to fluctuating energy supply, enhancing its reliability and efficiency under unstable energy conditions. As shown in Fig. 18b, the introduction of redox media provides necessary support for the electrolysis system, ensuring stable operation, particularly under fluctuating conditions. At the same time, Xia et al. addressed the issue of poor, inefficient, and inconsistent performance of AWE systems at low loads due to fluctuating renewable energy sources (REs) in large-scale hydrogen production (Fig. 18c). They proposed an optimal pulsed voltage duty cycle strategy, improving AWE efficiency and consistency through converter design and control technologies [160] (Fig. 18d). Experimental results showed that compared to conventional CE, the electrolysis efficiency of 2 Nm^3^ h^−1^ commercial AWE increased from 29.27% to 53.21% at rated load of 15%. Additionally, under conditions where system efficiency exceeded 50%, the operating range of the electrolyzer was expanded from 30% to 100% of the rated load to 10%–100%.

Another key technology in the field of renewable energy-driven hydrogen production via electrolysis is the development of stacks and electrolysis systems that can adapt to fluctuating energy supply conditions, ensuring their long-term stable operation [161, 162]. The development of long-life stacks focuses on optimizing the stack structure, catalyst materials, ion exchange membranes, and the micro-nanostructure and materials of the electrodes, covering both macro and microlevel optimizations. In contrast, the development of long-life electrolysis systems mainly concentrates on optimizing configuration methods and implementing effective process control technologies to extend the service life of equipment. It is worth noting that while previous studies have shown that PDE is beneficial for WE, the transient changes in electrolyzer parameters under actual wind and solar fluctuating power inputs could create localized stresses that significantly impact the system's lifespan.

Specifically, fluctuating power inputs can cause damage to catalyst materials and structures, making the search for higher-quality electrocatalyst materials a fundamental aspect of the long-term development and in-depth research of renewable energy-driven WE for hydrogen production. Additionally, fluctuating energy supply inputs reduce the performance and lifespan of electrolyzer. Frequent fluctuations in wind power can lead to frequent starts and stops of the electrolyzer, reducing hydrogen production and causing unnecessary losses in power electronic components. Even the most promising PEM electrolysis technology, under fluctuating input currents, can experience differential pressure fluctuations across the ion exchange membrane, causing membrane vibrations and damage, which ultimately reduces the operational lifespan of the electrolyzer.

### Solar and Wind Power Fluctuating Driven WE System

Currently, one of the factors limiting the large-scale application of WE is the high cost of hydrogen production. Integrating WE with renewable energy is key to reducing hydrogen production costs. However, the low conversion efficiency of renewable energy into electricity significantly hinders its use in WE. Additionally, some intermittent and seasonal renewable energies, including solar and wind power, are easily affected by external environmental factors, negatively impacting the power grid [163]. On shorter time scales, wind power exhibits random fluctuations on the order of minutes to hours, while PV generation follows basic diurnal cycle characteristics [164]. To improve the reaction efficiency of the electrolysis system and the yield of target products, researchers should innovate in the supply methods of reactants (unsaturated supply to avoid side reactions) and electrolytic methods (intermittent electrolysis). Exploring new renewable energy and enabling localized energy utilization are expected to promote the widespread development of WE.

#### Solar Hydrogen Production System

**Fig. 19:**
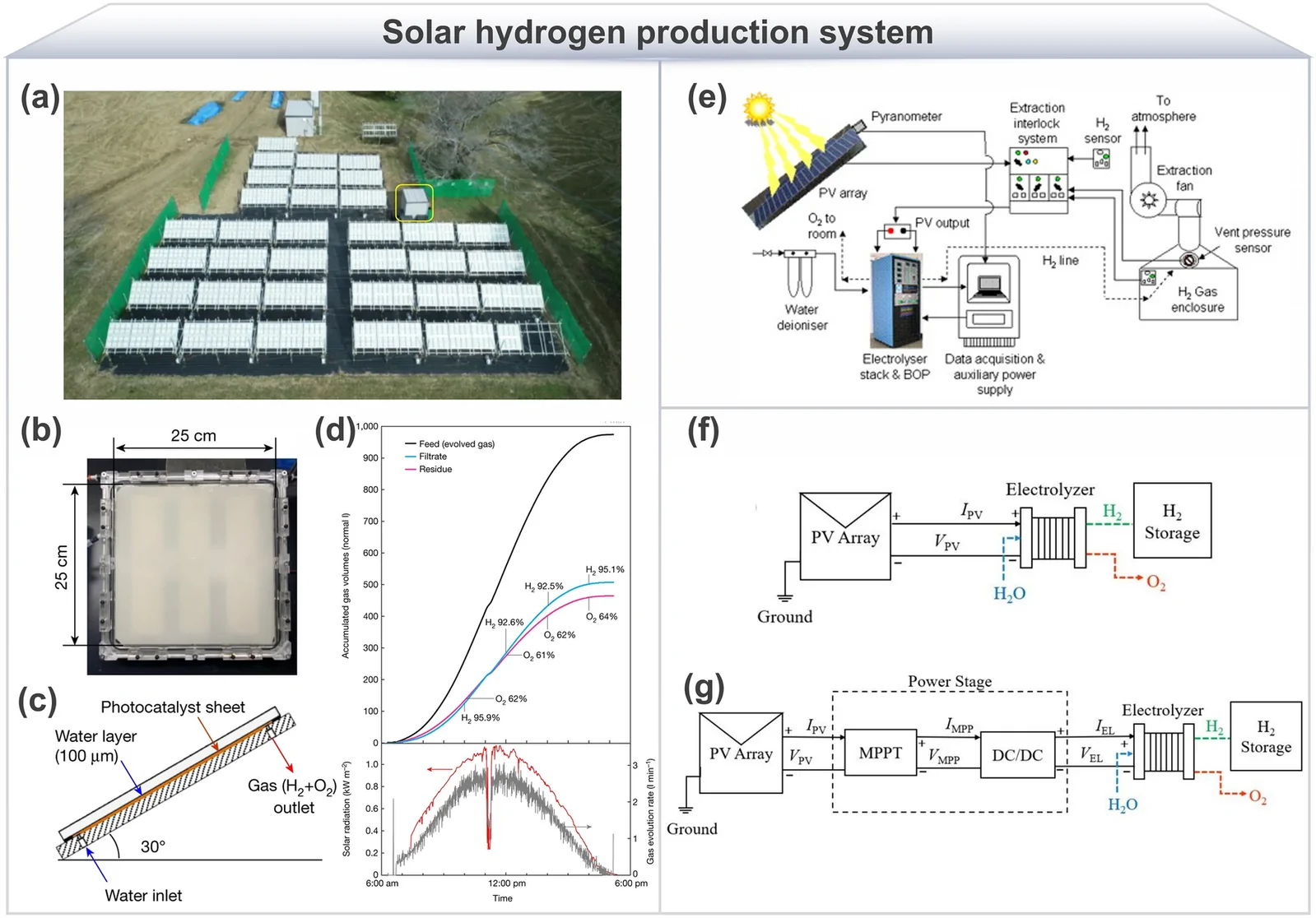
Solar hydrogen production system. **a** Top view of a 100 m^2^ solar hydrogen production system, including 1600 panel reactor units and small gas separation facilities (indicated by the yellow box). **b** Photograph and **c** structure (side view) of the panel reactor device (625 cm^2^). **d** Top: cumulative amounts of wet hydrogen, oxygen gas, filtrate, and residual gas, with the ratios of hydrogen and oxygen in the filtrate and residual gas (excluding water vapor) marked on the curves. Bottom: variation in solar irradiance (in red) and the rate of gas generation in the water splitting panel reactor (in gray). Reproduced with permission from Ref. [169]. Copyright 2021, Springer Nature. **e** Schematic diagram of the direct coupling of the PV array with PEM cell. Reproduced with permission from Ref. [170]. Copyright 2009, Elsevier. **f** Direct coupling and **g** indirect coupling configurations and their main components schematic. Reproduced with permission from Ref. [173]. Copyright 2024, Elsevier

In various green energy-driven WE systems, there are two main approaches to solar driven hydrogen production: PV indirect coupling hydrogen production and PV direct coupling hydrogen production systems. The commonly used method for PV-DC indirect coupling hydrogen production is maximum power density tracking, such as adjusting the duty cycle with PWM to track the maximum power point and implementing robust control to regulate the converter output current [165, 166]. Although PV direct coupling hydrogen production simplifies the complexity of the hydrogen production system, the voltage and current waveforms from the PV batteries directly affect the electrolyzer, posing challenges to the long-term safe and stable operation of the electrolyzer. The hydrogen production efficiency of this technology is still limited by factors such as cost and efficiency [167]. Currently, the efficiency of industrial AWE systems and mainstream solar panels is approximately 70% and 18%, respectively, with a hydrogen cost of around $10 kg^−1^ [168]. Nishiyama et al. achieved a maximum solar hydrogen production efficiency of 0.76% through PV direct coupling, based on a modified SrTiO_3_:Al particle catalyst, by scaling up a 1 m^2^ array plate reactor system to a 100 m^2^ array [169]. This was accomplished with safe and sustained hydrogen production, though the hydrogen production efficiency is not high, and the overall system is negative in energy. They also demonstrated the feasibility of large-scale industrial solar catalytic water splitting, gas collection, and separation (Fig. 19a-d). In terms of directly coupling PEM cell with a matching solar PV source, Clarke et al. conducted experiments and found that, when the initial input current was 50 A, the electrolyzer efficiency was approximately 91%. However, after the stack ran for hundreds of hours, undergoing multiple forced and non-forced shutdowns, as well as frequent start-stop switches, the electrolyzer efficiency dropped to about 75% [170] (Fig. 19e). Therefore, to mitigate excessive power fluctuations, the input current and voltage must be limited to a certain range. Additionally, the efficiency of hydrogen mixing systems coupled with solar power is crucial. Paul et al. found that the hydrogen production efficiency of solar coupled mixed system can be as high as 95%, based on a 3-h test conducted only during peak solar radiation [171]. This suggests that 95% of the power generated by the PV panels can be supplied to the electrolyzer, and when the output of the PV system matches the input requirements of the electrolyzer, the system can achieve up to 95% efficiency. However, the coupling efficiency of commercially available solar coupled hydrogen production systems is typically below 12%, with some systems even as low as 2.3% [155, 172]. Such low efficiency in solar hydrogen systems significantly increases the overall electricity production cost, thereby limiting the application of such systems.

Another approach is to couple the PV panels with the electrolyzer through voltage regulator to provide constant voltage to the electrolyzer. Valle et al. conducted a comparative analysis of direct and indirect electrical coupling configurations between PV systems and electrolyzers. The results showed that under typical meteorological conditions, the hydrogen production in the indirect coupling method was 37.5% higher than that in the optimized direct coupling method [173] (Fig. 19f, g). This difference increased the overall solar-to-hydrogen efficiency from 5.0% in direct coupling to 6.9%. However, some researchers have pointed out that while indirect coupling has advantages in adapting to variable solar radiation and environmental temperature, it also increases system costs and the complexity of DC-DC connections, leading to higher energy losses during the energy transfer process [174]. Therefore, although indirect coupling has advantages in energy management strategies and utilizing excess energy, it does not have a clear advantage over direct coupling in optimization models that minimize energy losses or maximize hydrogen production. To improve the cost-effectiveness of indirect coupling, future research may need to explore optimized designs to reduce energy transfer losses while maximizing system adaptability and efficiency.

In general, the low efficiency of solar hydrogen hybrid systems is not only due to the efficiency losses of the PV panels and the electrolyzer itself but also involves the energy consumption of electronic control and temperature regulation systems. However, the main efficiency losses still stem from the insufficient coupling efficiency between the PV panels and the electrolyzer. Although directly coupling the PV system with the electrolyzer is currently considered the most cost-effective solution, this method still faces challenges, particularly the instability of solar radiation intensity and the strict power input requirements of the electrolyzer. When the output power of the PV system exceeds or falls below the tolerable range of the electrolyzer, the electrolyzer stops working, which affects the overall system stability and hydrogen production efficiency [175, 176]. Therefore, it is necessary to develop low-cost and efficient systems for more effective coupling between PV panels and the electrolyzer. This not only requires the PV system to adapt to changes in solar radiation intensity but also necessitates the optimization of electrolyzer design to ensure efficient and stable operation over wider power fluctuation range. More importantly, a system needs to be designed that can dynamically adjust the matching between the output of the PV panels and the power demand of the electrolyzer, ensuring efficient hydrogen production even under unstable solar radiation conditions. This requires researchers to delve deeper into fields such as power electronics, intelligent control technology, and energy storage systems to enhance the overall efficiency and reliability of the system.

#### Wind Power Hydrogen Production System

**Fig. 20:**
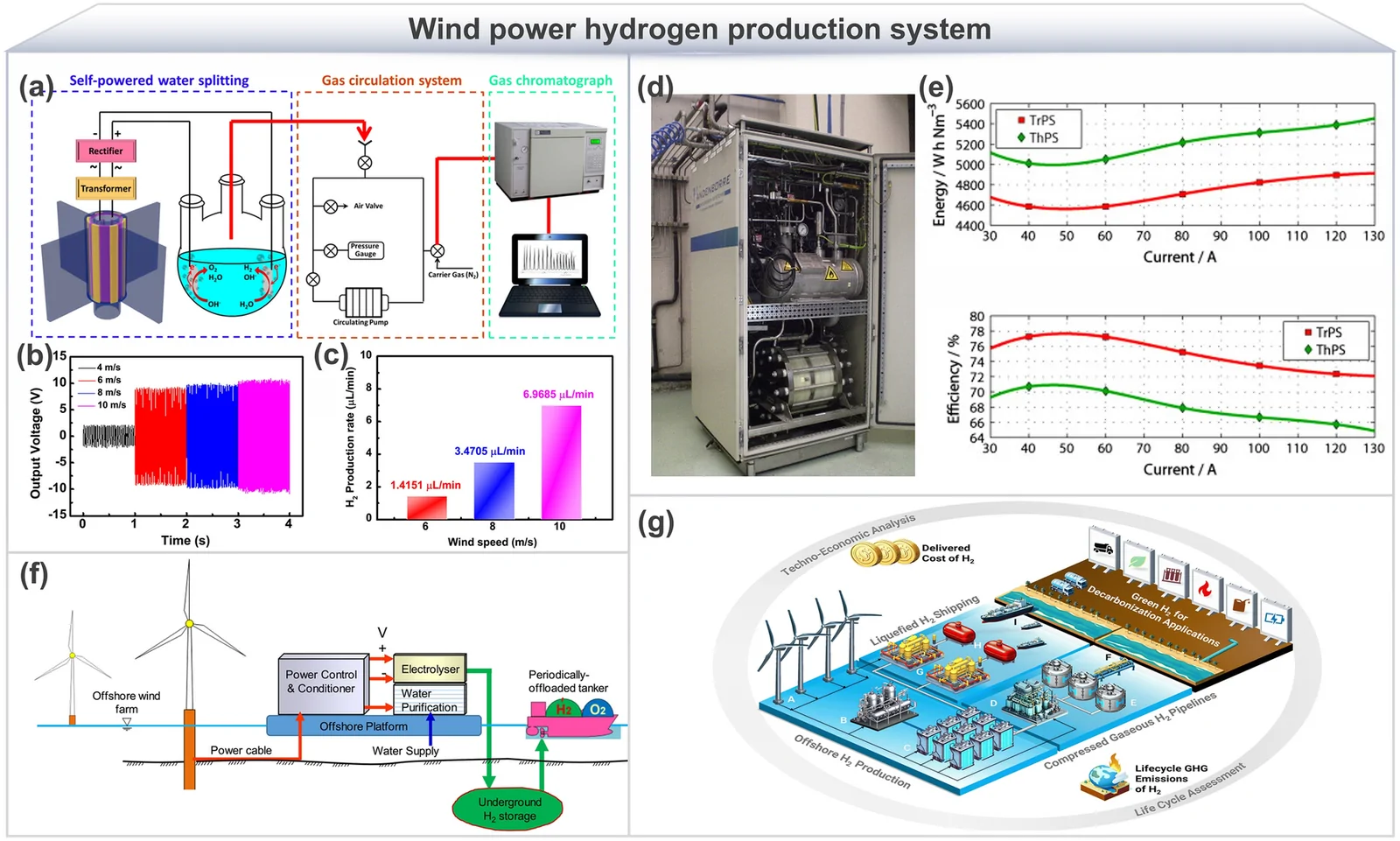
Wind power hydrogen production system. **a** Schematic of the CRF-TENG wind power collector driving self-powered WE system. **b** Output voltage and **c** hydrogen production rate of the CRF-TENG wind power collector at different wind speeds. Reproduced with permission from Ref. [177]. Copyright 2018, Elsevier. **d** Photograph of a commercial AWE. **e** Electrolyzer stack energy consumption and efficiency (operating conditions 20 bar and 65 °C). Reproduced with permission from Ref. [178]. Copyright 2009, Elsevier. **f** Schematic of hydrogen production, underground storage, and periodic tanker transportation from offshore wind farm. Reproduced with permission from Ref. [180]. Copyright 2021, Elsevier. **g** Schematic of offshore wind power hydrogen production system. Reproduced with permission from Ref. [181]. Copyright 2024, Royal Society of Chemistry

Wind power applications in WE for hydrogen production have shown significant potential, especially compared to solar, as wind power is not restricted by time and can continuously supply power even without sunlight, providing a reliable energy source. This makes it one of the ideal energy sources for hydrogen production. Here, Ren et al. successfully achieved environmental wind power collection using coaxial rotating independent triboelectric nanogenerator (CRF-TENG) wind power harvester and utilized the generated electrical energy to perform water splitting for hydrogen production without relying on external power sources (Fig. 20a). As shown in Fig. 20b, c, when the wind speed was 10 m s^−1^, the hydrogen generation rate reached 6.9685 μL min^−1^ in 1 M KOH solution [177]. Although this method demonstrates feasibility and high efficiency, some challenges remain. Currently, the efficiency and output power of nanogenerators are relatively low, and practical applications may require optimized designs and improved wind power capture efficiency to enhance their application value. Future research could further explore how to enhance wind power capture capabilities, increase hydrogen generation rates, and optimize the stability and sustainability of the system, thereby promoting the widespread application of this technology. At present, there is a lack of tools to analyze the efficiency and operation of electrolyzers under wind power sources. To address these issues, Ursúa et al. proposed a novel electrolyzer power simulator for electrical and electronic equipment, simulating wind and photovoltaic generators powering the electrolyzer based on two representative power supplies (Thyristor Power Supply, ThPS, and Transistor Power Supply, TrPS) (Fig. 20d), and analyzed their performance under renewable energy supply to assess and compare the effects of the power supply on any commercial AWE. Using ThPS, the minimum energy consumption was 4995 W·h N·m^−3^, with a corresponding maximum efficiency of 70.9%. However, using TrPS, the energy consumption was 4560 W·h N·m^−3^, and the efficiency was 77.6%. Compared to ThPS, the energy efficiency of the electrolyzer stack improved by 9.2% to 10% when using TrPS [178] (Fig. 20e). Currently, there are few reports on the electrical perspective of electrolysis. Electrical characteristics of the system, such as voltage, current, and current density, are typically used to validate theories or serve as evidence for comparing different electrochemical methods. However, the applied power is an important aspect of the electrolysis process, and more research can be conducted in this field in the future.

In the process of using wind power for WE to produce hydrogen, in addition to the technical feasibility and efficiency, cost and environmental impact are also important factors that need attention. In recent years, an increasing number of studies have begun to assess the environmental impact and economic feasibility of different hydrogen production methods. Acar et al.’s research indicates that although the cost of wind and solar power-based hydrogen production is higher than traditional hydrogen production methods, it can effectively suppress global acidification trends and have a positive impact on mitigating climate change [179]. By optimizing the configuration of wind power curtailment capacity for hydrogen production, Dinh et al. proposed using interval estimation methods to establish a wind power annual curtailment power statistical model, with the goal of maximizing economic benefits. They applied interval optimization theory to determine the optimal capacity configuration range for the hydrogen production system. By establishing a multi-attribute decision model, they determined the optimal electrolyzer configuration for the hydrogen production system [180] (Fig. 20f). Offshore wind power not only has significant renewable energy potential but can also greatly promote the production of green hydrogen, thus effectively advancing the decarbonization process. Balaji et al. used optimization frameworks and life cycle assessment methods to analyze the economics and environmental impact of offshore green hydrogen production (Fig. 20g). The study results show that, under an optimistic scenario, deploying 0.96 TW of offshore wind power capacity could meet 75% of the hydrogen consumption demand in the United States. The utilization rate of offshore wind resources would increase from the current 1% to over 22% of the technical resource potential. They predicted that the delivery cost of offshore hydrogen production would range from $2.50 to $7.00 kg^−1^, and the life cycle greenhouse gas emissions of offshore hydrogen production would be lower than the coastal benchmark (4 kg CO_2_e kg^−1^ H_2_) [181]. Although the initial cost is higher, with technological advancements and large-scale deployment, the hydrogen production cost is expected to decrease significantly, further enhancing its competitiveness. However, challenges such as offshore wind power infrastructure construction, maintenance, and technological difficulties related to land connections still need to be overcome. Therefore, future research should further optimize the integration of offshore wind power with hydrogen production to promote sustainable development.

In conclusion, the application of wind power in WE provides a stable and sustainable energy source for green hydrogen production, with significant economic potential and environmental benefits, particularly in driving the green transition. To further improve the system’s overall energy efficiency and extend the lifespan of the equipment, future research could optimize the electrolyzer’s workload by combining advanced artificial intelligence prediction techniques with fast response control systems. This synergistic optimization approach would not only significantly enhance overall efficiency but also ensure that the electrolyzer operates under optimal conditions, thus promoting further improvements in the economic viability and sustainability of wind based hydrogen production technology.

## Conclusions and Perspectives

Renewable energy-driven PDE stands as a critical solution for integrating renewable energy generation, offering an effective strategy to combat environmental pollution and energy shortages resulting from an overreliance on fossil fuels. Overall, PDE can regulate various parameters such as current/voltage, frequency, and duty cycle to alter the diffusion process of reactants, affecting species transfer, adsorption, and electrocatalytic reactions at the electrode/solution interface. This not only enhances the target reaction through local microenvironment regulation but may also significantly improve the physical phase environment of electrocatalysts, extending their lifespan. Additionally, from the long-term perspective of electrocatalysis development, current supply methods could be developed into real-time dynamic regulation based on changes in electrolyte phase and electrode/solution interface microenvironments. This could further match the needs of electrochemical reactions and species mass transfer rates, enabling deep regulation of interface material transfer and energy conversion processes, and thereby building more efficient and low-energy consumption systems.

**Fig. 21:**
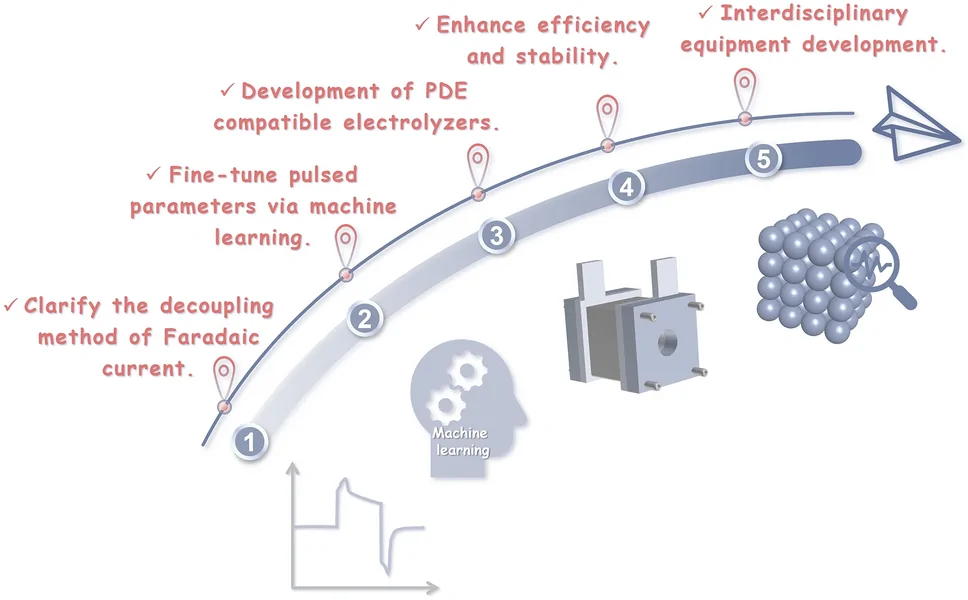
Future directions for advancing PDE systems

Despite notable strides in fields such as CO_2_RR, electro-oxidation, and electrosynthesis, research on mass and energy transfer mechanisms and WE for hydrogen production using PDE is still in its early stages. To better understand the mechanisms of PDE in mass and energy transfer and enhanced hydrogen production, this review delves into the effects of PDE on factors such as intermediate adsorption/desorption, disturbance of the EDL, local pH, and extension of the lifespan of electrolysis system. The review also summarizes the enhancement mechanisms of PDE in hydrogen production and the key influencing factors. Additionally, the review focuses on the key technologies for WE hydrogen production under fluctuating power inputs. However, despite the promising prospects provided by current research for the industrial application of PDE, it also underscores several key issues that merit attention in forthcoming studies (Fig. 21):

1. Clarifying and quantifying the decoupling method of electrolysis time and current contribution. The selection of pulsed waveform, operating time, and current contribution decoupling methods can significantly affect the efficiency and selectivity of the target product. However, there are inconsistencies in comparing pulsed operating time (including pulsed interval time) with constant voltage operating time. In this context, future research should focus on how to accurately define and compare the complete operating time of PDE with the actual electrolysis time under CE, avoiding errors caused by normalization. Moreover, the contribution of Faradaic current and non-Faradaic current (from EDL charging) in pulsed waveforms has yet to be decoupled, and there is a lack of widely recognized theoretical standards or methods. Therefore, establishing a unified theoretical framework and experimental standards will help evaluate the effectiveness of the PDE process more accurately, advancing the commercialization of PDE.
2. Fine-tuning pulsed parameters via machine learning. The selection of pulsed amplitude, frequency, and duty cycle plays a crucial role in the efficiency and selectivity of the reaction. Under different pulsed parameter conditions, the electrochemical reaction dynamics and the transport characteristics of reactants and products change, thus affecting the efficiency and selectivity of reaction. Identifying the optimal combination of pulsed voltage and frequency to maximize WE efficiency is key to significantly improving experimental efficiency. Traditional optimization methods rely heavily on trial-and-error experiments, which is effective but inefficient and labor-intensive. By leveraging machine learning, particularly neural networks, it is possible not only to gain deeper insights into the electrochemical reaction behaviors under different pulsed conditions, but also to combine multi-objective optimization algorithms, response surface models, and feature engineering to establish a systematic mapping between pulsed parameters and reaction performance. This enables precise prediction of the optimal pulsed parameter combinations and advances the theoretical development of pulsed parameter optimization. Thus, it improves experimental efficiency, reduces the need for high-throughput experiments, and provides scientific guidance for parameter selection.
3. Design and development of electrolyzer devices compatible with PDE. The PDE enables dynamic regulation of the electrode interfacial microenvironment, which can not only effectively suppress bubble accumulation and enhance mass transfer efficiency, but also mitigate concentration polarization. As a result, it improves hydrogen production rates while simultaneously reducing energy consumption. However, effectively integrating PDE into existing electrolyzer device designs, especially in electrolyzer stack systems, is a critical challenge for its large-scale application. The periodic changes in pulsed current and voltage can significantly alter the flow state and gas–liquid interface behavior inside the electrolyzer, affecting the reaction efficiency and selectivity. Traditional electrolyzer designs usually do not account for the effect of the frequent fluctuations caused by PDE on the flow field and electrode surface reactions, which may limit the effectiveness of PDE. Therefore, future research should focus on optimizing the flow channel design, electrode configuration, and device structure of electrolyzers to ensure that the flow field and gas–liquid distribution can adapt to pulsed operation requirements. Additionally, developing adaptive electrode materials and innovative electrode structures that meet the specific needs of PDE is crucial for ensuring efficient electrolysis and long-term stable operation. Future studies should aim to closely match electrolyzer designs with PDE modes to improve overall system performance and stability.
4. Enhancing energy efficiency and stability. PDE effectively mitigates electrode surface deactivation and suppresses side reactions by periodically adjusting current and voltage. However, pulsed operation may lead to instantaneous energy fluctuations and increased equipment load, resulting in energy loss and decreased system stability. Therefore, optimizing pulsed conditions to improve energy utilization efficiency and extend system lifespan is a challenge for future research. Specifically, whether PDE can maintain the gains observed in laboratory settings under industrial high-temperature and high-pressure conditions in practical applications needs further experimental verification. Additionally, there is a lack of experimental data on solid oxide electrolysis cells (SOEC), and the application of PDE in this field has not been thoroughly explored. Especially under high current densities and elevated temperatures, ensuring system stability and minimizing energy consumption will be crucial for advancing PDE toward industrial-scale implementation.
5. Cross-disciplinary equipment development and standardization of testing. To ensure the comparability of results across different studies, there is an urgent need to develope high-quality testing equipment and establish unified testing standards. The application of pulsed voltage can alter the adsorption state of molecules around the electrode and the ion migration, affecting the EDL and local pH distribution at the electrode interface. These local changes not only affect the adsorption behavior of the catalyst surface but can also lead to the accumulation or excessive consumption of reaction intermediates. Given the complexity of the transient processes triggered by PDE, developing high-precision testing equipment is essential for in situ real-time characterization of these processes. Combining in situ spectroscopic techniques (such as XAFS, Raman, FTIR, and XPS), in situ electron microscopy techniques (such as high-speed imaging, TEM, LSCM, and STM), and in situ gas analysis techniques (such as DEMS), it is possible to reveal, from multiple dimensions, the dynamic evolution of active site mechanisms, interfacial reaction kinetics, and the transport pathways of reactants/products (including intermediate species) during the PDE process. This will facilitate a deeper understanding of the regulation mechanism of PDE and its structure–activity relationship. Furthermore, properly designing electrolyzer and electrical control systems can help suppress unnecessary voltage fluctuations from renewable energy sources, improving system stability and operational efficiency.

With the continuous advancement of testing and characterization technologies and the gradual improvement of pulsed integration systems, the application prospects of PDE driven by renewable energy are becoming increasingly broad. By addressing the key issues outlined above, especially in areas such as pulsed parameter regulation, electrolyzer device design, energy efficiency optimization, and interdisciplinary research, the industrialization of PDE will be significantly accelerated, making an important contribution to addressing global energy and environmental challenges.
